# Beta-Glucan Supplementation Improves Clinical Outcomes and Preserves Immune Homeostasis in Patients with Advanced Solid Tumors Receiving Chemotherapy: A Prospective Phase II Randomized Three-Arm Clinical Trial

**DOI:** 10.3390/ijms27167189

**Published:** 2026-08-11

**Authors:** Wen-Chi Yang, Ming-Shyan Huang, Yi-Hong Tsai

**Affiliations:** 1 Department of Nursing, Meiho University, PingTung 912, Taiwan; 2 Division of Hematology and Medical Oncology, Department of Internal Medicine, Ping-Tung Veterans General Hospital, Pingtung 900, Taiwan; 3 School of Medicine, College of Medicine, National Sun Yat-Sen University, Kaohsiung 804, Taiwan; 4 Division of Chest, Department of Internal Medicine, E-DA Cancer Hospital, Kaohsiung 824005, Taiwan; 770261@gmail.com; 5 Department of Pharmacy and Master Program, College of Pharmacy and Health Care, Tajen University, Pingtung County 90741, Taiwan; bear@tajen.edu.tw

**Keywords:** β-Glucan, bioactive protein from bovine colostrum, immune profile, CD158 (KIR) NK cells, N/L ratio

## Abstract

Chemotherapy-induced immune dysregulation remains a major challenge in patients with advanced solid tumors. β-Glucan has been reported to possess immunomodulatory properties; however, its longitudinal effects on immune homeostasis during chemotherapy remain unclear. In this prospective, randomized phase II study, patients receiving chemotherapy were assigned to receive β-glucan powder (with L-glutamine and bioactive protein from bovine colostrum), β-glucan capsules (with L-glutamine), or standard care. Clinical outcomes, serial hematologic parameters, longitudinal immune profiling, and an in vitro Jurkat T-cell viability assay were evaluated. Compared with controls, β-glucan supplementation improved the disease control rate, accelerated neutrophil recovery, and maintained a more stable neutrophil-to-lymphocyte ratio during chemotherapy. Longitudinal immune profiling demonstrated greater stability of T-cell, NK-cell, NKT-cell, and KIR (CD158)-expressing immune-cell populations in β-glucan supplementation groups, suggesting attenuation of chemotherapy-induced immune remodeling. Both β-glucan formulations showed comparable immunomodulatory effects, although the expression of CD158b(+) and CD158i(+) NK-cell subsets remained more stable in the powder group during later treatment. In vitro, β-glucan significantly reduced Jurkat T-cell viability, indicating direct biological activity in addition to its clinical immunomodulatory effects. These findings suggest that β-glucan supplementation helps preserve immune homeostasis during chemotherapy and may serve as a promising adjunctive immunonutritional strategy for patients with advanced solid tumors.

## 1. Introduction

With changes in environmental factors and population demographics, cancer remains the leading cause of death in Taiwan. Chemotherapy is one of the main treatment options of advanced/late stage cancer patients. Cytotoxic chemotherapy suppresses the hematopoietic system, impairs host immune defense, and often limits chemotherapy dose intensity of chemotherapy. Among critically ill cancer patients receiving chemotherapy, 22.6% of them develop neutropenia [1]. Cancer patients with febrile neutropenia have a higher risk of mortality than those without febrile neutropenia [2].

β-glucans are one of the most abundant forms of polysaccharides in the cell walls of bacteria and fungi. They have been reported to possess immune-modulating properties, such as anti-inflammatory or antimicrobial abilities [3,4,5]. All β-glucans are glucose polymers linked together by a 1,3 linear β-glycosidic chain core and differ from each other by their length and branching structures: 1,4- or 1,6-glycosidic chains, mainly [6]. Not all β-glucans are able to modulate immune functions. Only 1,3-βlinked backbone with small numbers of 1,6-βlinked side chains are recognized to possess potent immune-modulating effects [7].

Cancer immunity is important in inhibitory carcinogenesis and controlling cellular homeostasis that provides host protection. The immune cells, including natural killer (NK) cells, dendritic cells (DCs), macrophages, polyneuclear cells (including neutrophil, eosinophil and basophil), mast cells and cytotoxic T lymphocytes, directly target tumor cells [8,9,10]. NK cells, DCs, PMN, mast cells, and macrophages are first-line effectors to damaged cells and cancer cells. Natural killer T cells (NKTs) and γδ T cells play roles as both innate and adaptive components, through close interactions with cells of the adaptive immune system, such as CD4^+^ and CD8^+^ T lymphocytes, which possess cytotoxic activity and immunological memory [10]. β-(1,3)-Glucans have been reported to synergistically enhance the antitumor efficacy of antitumor antibodies in murine studies [11]. β-Glucans have been reported to serve as promising adjuvants in cancer therapy, particularly through synergistic effects with chemotherapy and immunotherapy [12]. In our study, we present the influence of β-glucans on leukocytes, neutrophils, NK cells and PD-1 expression in stage IV cancer patients who received cytotoxic chemotherapy treatment.

## 2. Results

### 2.1. Patients’ Characteristics

**Figure 1:**
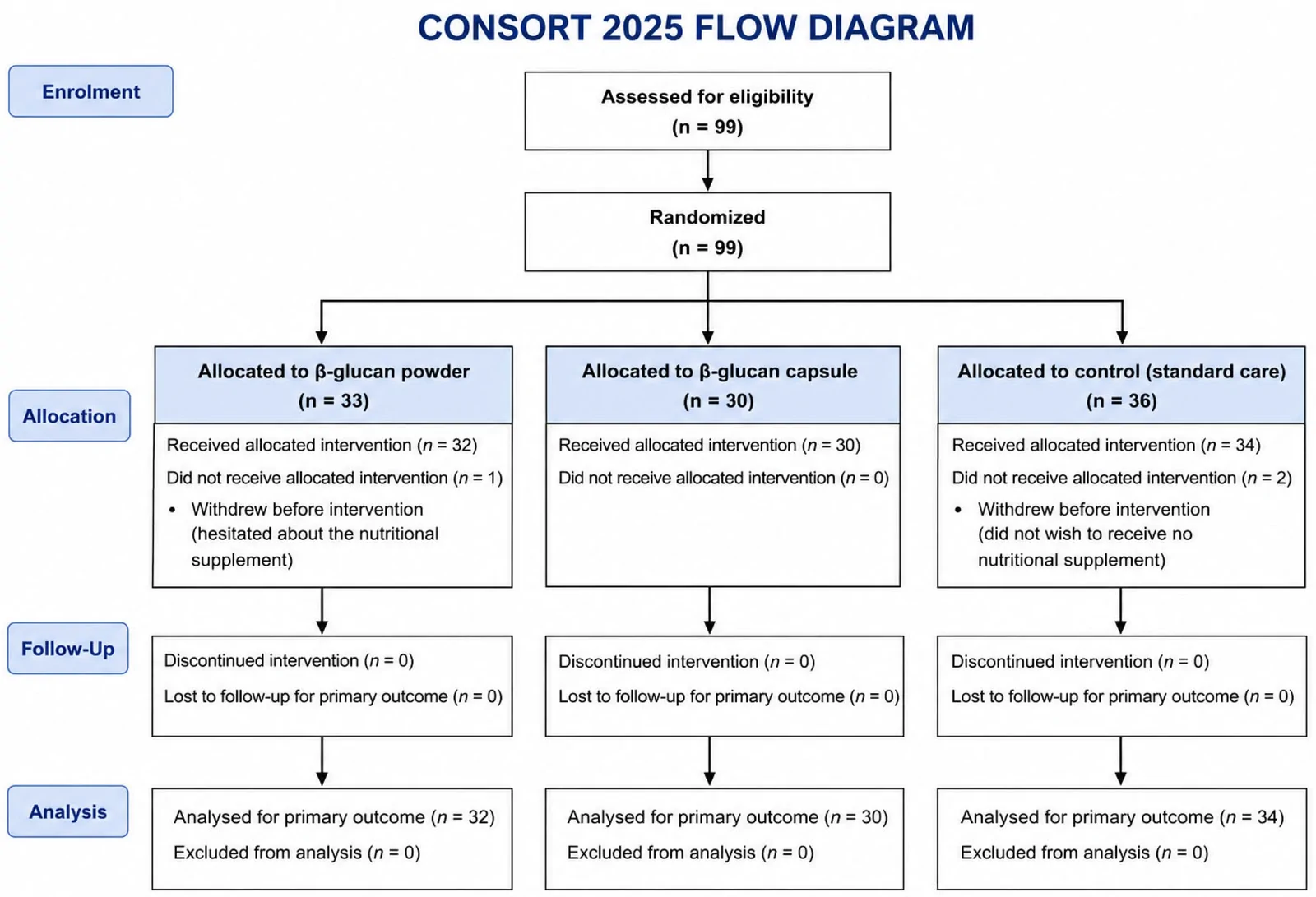
CONSORT diagram of this clinical trial.

We enrolled a total of 99 patients and assigned each to a group using a computer-generated random-number sequence. One patient in group 1 (β-glucan soluble WGP powder) withdrew after signing inform consent due to hesitation in response to the nutritional products. Two patients assigned to the control group (group 3) withdrew because they preferred receiving β-glucan supplementation rather than observation alone. We enrolled 32 patients in group 1, 30 patients in group 2 (β-glucan capsule) and 34 patients in the control group from 4 January 2018 to 30 December 2020, and a follow up until 5 October 2022 in E-Da Hospital (CONSOFT diagram, Figure 1). White blood cells surface markers were analyzed in all patients in the study group and only seven patients in the control group. There were no different in age, cancer type and G-CSF use after starting β-glucan intervention. However, patients taking powder appeared to require less G-CSF compared with those taking a capsule (Table 1). The treatment response was better in the study group (≥SD: group 1 vs. 2 vs. 3= 81.25% vs. 66.66% vs. 52.94%, *p* = 0.039). Although there is no statistically significant difference, the infection rates were lower in the β-glucan intervention group, especially in patients who received β-glucan soluble WGP powder, compared with the observation group.

### 2.2. β-Glucan Supplementation Was Not Associated with Improved Survival

**Figure 2:**
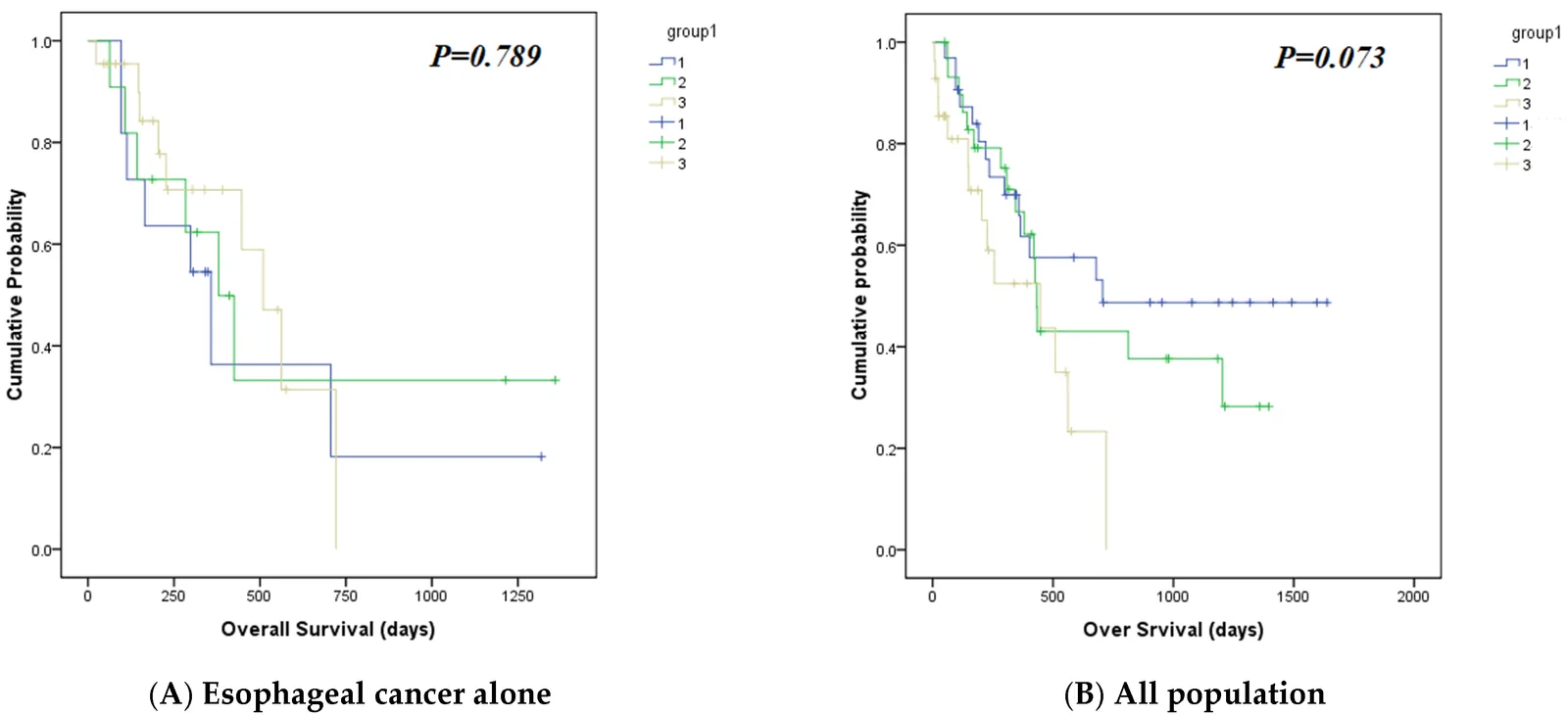
Overall survival in (**A**) esophageal cancer patients and (**B**) all cancer population, between different groups.

Because of the heterogeneous cancer types included in this study, the survival analysis may not accurately reflect the treatment effect. Therefore, we performed a subgroup analysis of patients with esophageal cancer, which represented the largest subgroup in our cohort. No significant difference in overall survival was observed either in patients with esophageal cancer alone or in the overall study population (Figure 2A,B).

### 2.3. β-Glucan Improved Chemotherapy-Related Bone Marrow Suppression

**Figure 3:**
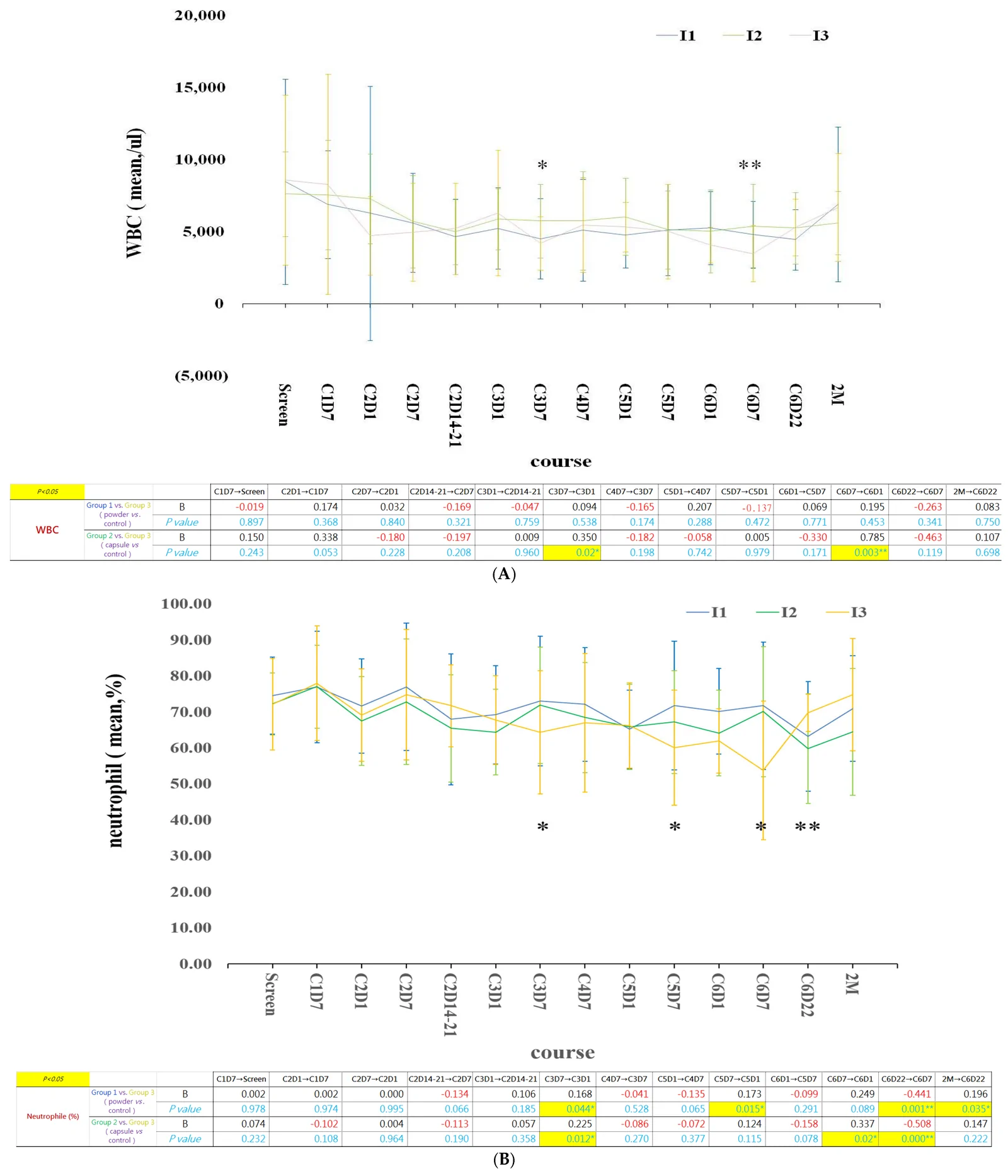

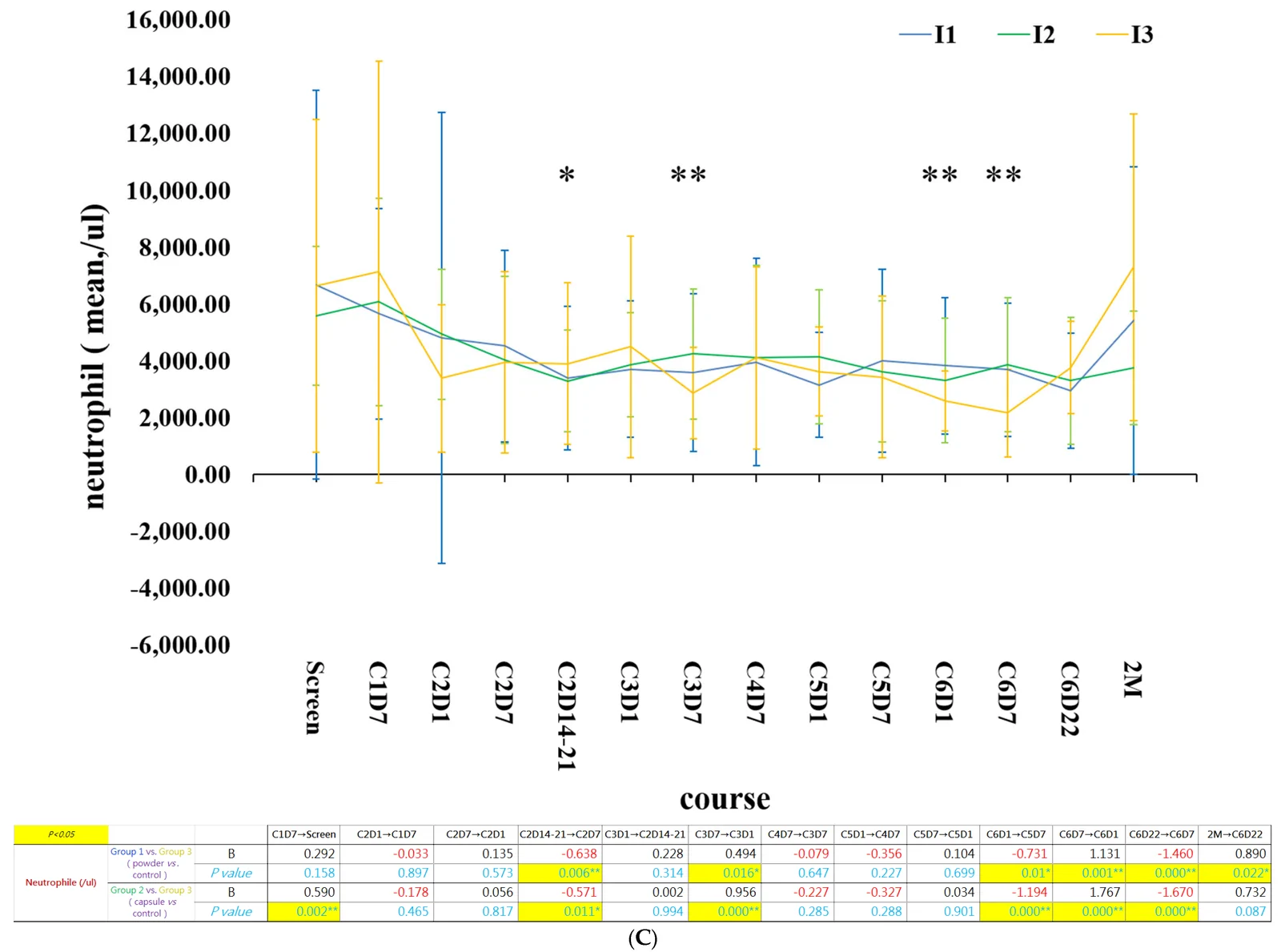
The longitudinal change of (**A**)White blood cell count; (**B**) neutrophil percentage; (**C**) neutrophilcount, after β-glucan intervention. β-glucan intervention since cycle 2 day 7 of chemotherapy (C2D7). Yeast-derived β-glucan supplementation was initiated on day 7 of cycle 2 of systemic chemotherapy. The blue, green and yellow lines represent group 1 (powder), 2 (capsule) and 3 (control). Data are expressed as mean ± SD. * *p* < 0.05; ** *p* < 0.01.

Baseline bone marrow function, including white blood cell (WBC) count, hemoglobin (Hb), platelet (PLT) count, neutrophil count, lymphocyte count, and the neutrophil-to-lymphocyte (N/L) ratio, is presented in Supplementary Figure S1. In the β-glucan intervention groups, peripheral white blood cell and neutrophil counts recovered significantly after 3–4 weeks (one chemotherapy cycle) of supplementation compared with those in the control group (Figure 3). The detailed longitudinal data for WBC, Hb, PLT, neutrophil, lymphocyte, and monocyte counts for each patient are presented in Supplementary Figures S2–S7.

### 2.4. The N/L Ratio Decreased After Two Cycles of β-Glucan Intervention and Became Stable Thereafter

**Figure 4:**
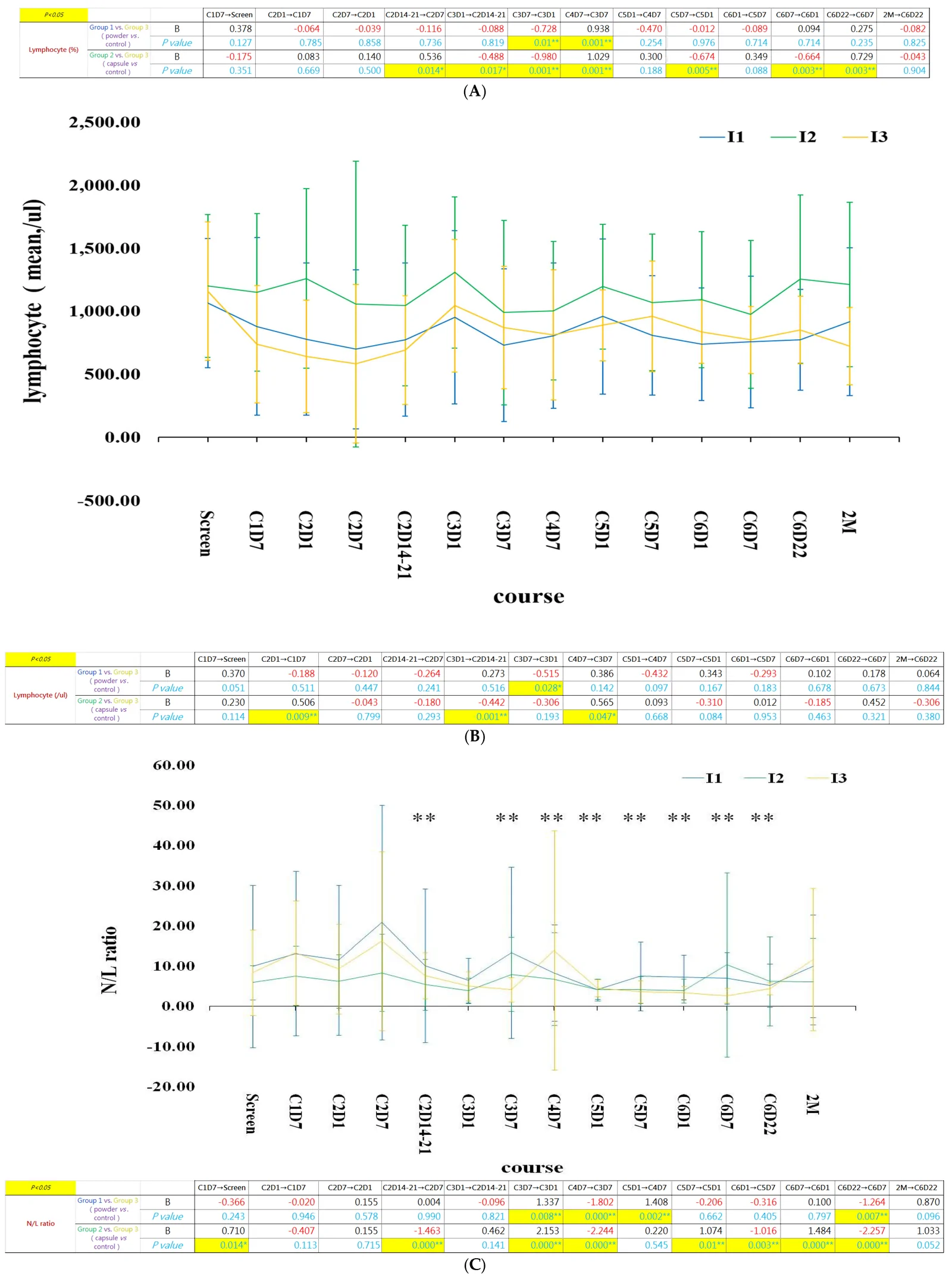
(**A**) Lymphocytes percentage, (**B**) lymphocyte count, and (**C**) N/L in β-glucan intervention groups and control group. More stabilized lymphocytes and N/L ratio were noticed in β-glucan intervention groups. Yeast-derived β-glucan supplementation was initiated on day 7 of cycle 2 of systemic chemotherapy. The blue, green and yellow lines represent group 1 (powder), 2 (capsule) and 3 (control). Data are expressed as mean ± SD. * *p* < 0.05; ** *p* < 0.01.

The neutrophil-to-lymphocyte (N/L) ratio is a well-established indicator of systemic inflammation and poor prognosis in patients with cancer. The N/L ratio decreased after 6–8 weeks of β-glucan supplementation and remained stable after the fifth cycle of treatment in the β-glucan intervention groups (Figure 4). This finding was associated with a greater increase in lymphocytes than in neutrophils. A more stable pattern of lymphocyte percentages was observed in the β-glucan intervention groups. Overall, patients receiving β-glucan powder showed a more stable and lower N/L ratio, which may predict better clinical outcomes.

### 2.5. β-Glucan Powder/Capsule May Stabilize Immune-Cell Trajectories During Chemotherapy, Whereas the Control Group Showed Greater Fluctuation in NK/KIR- and PD-1-Related Subsets

#### 2.5.1. β-Glucan Stabilized Helper and Cytotoxic T-Cell Populations During Chemotherapy

**Figure 5:**
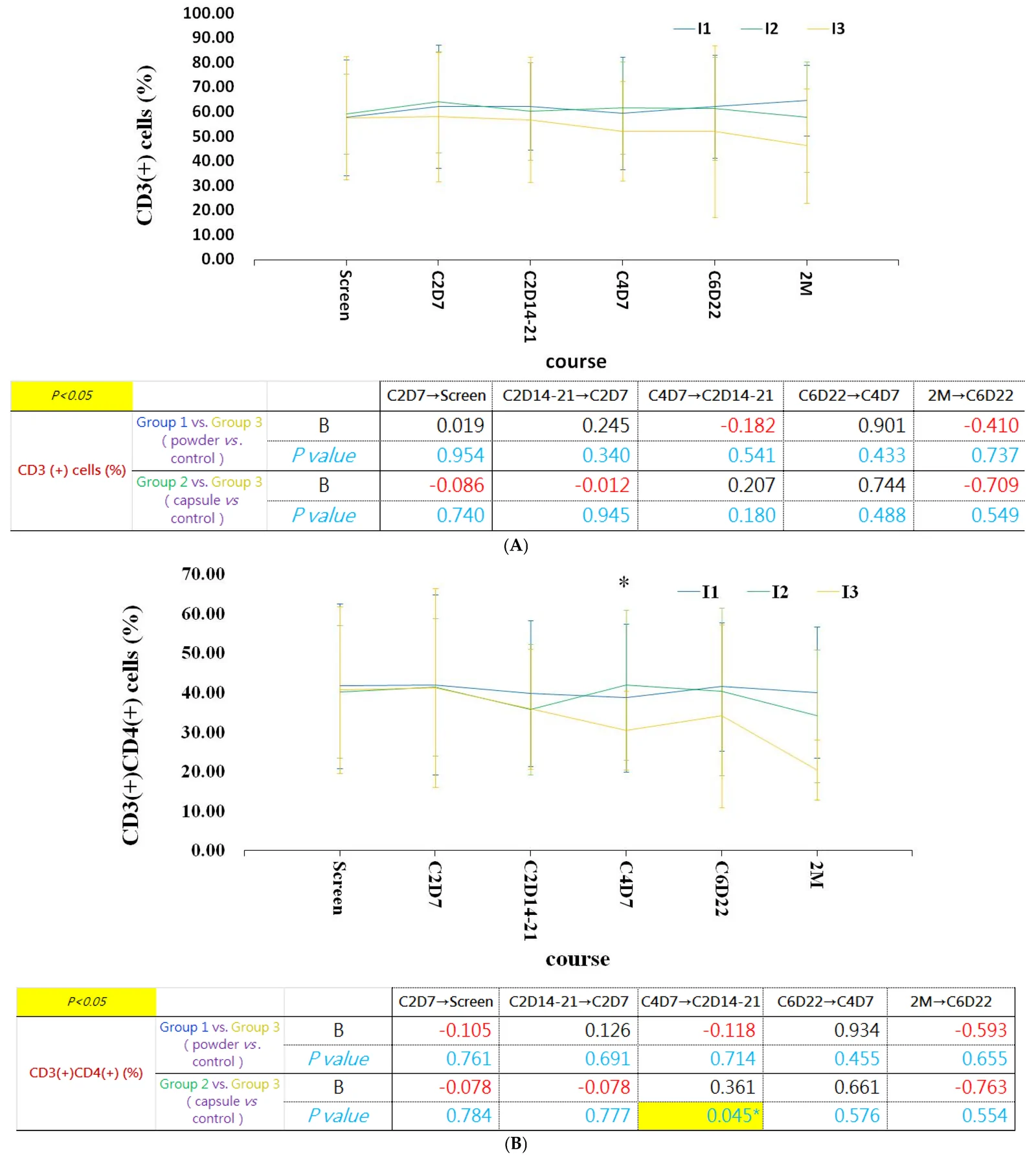

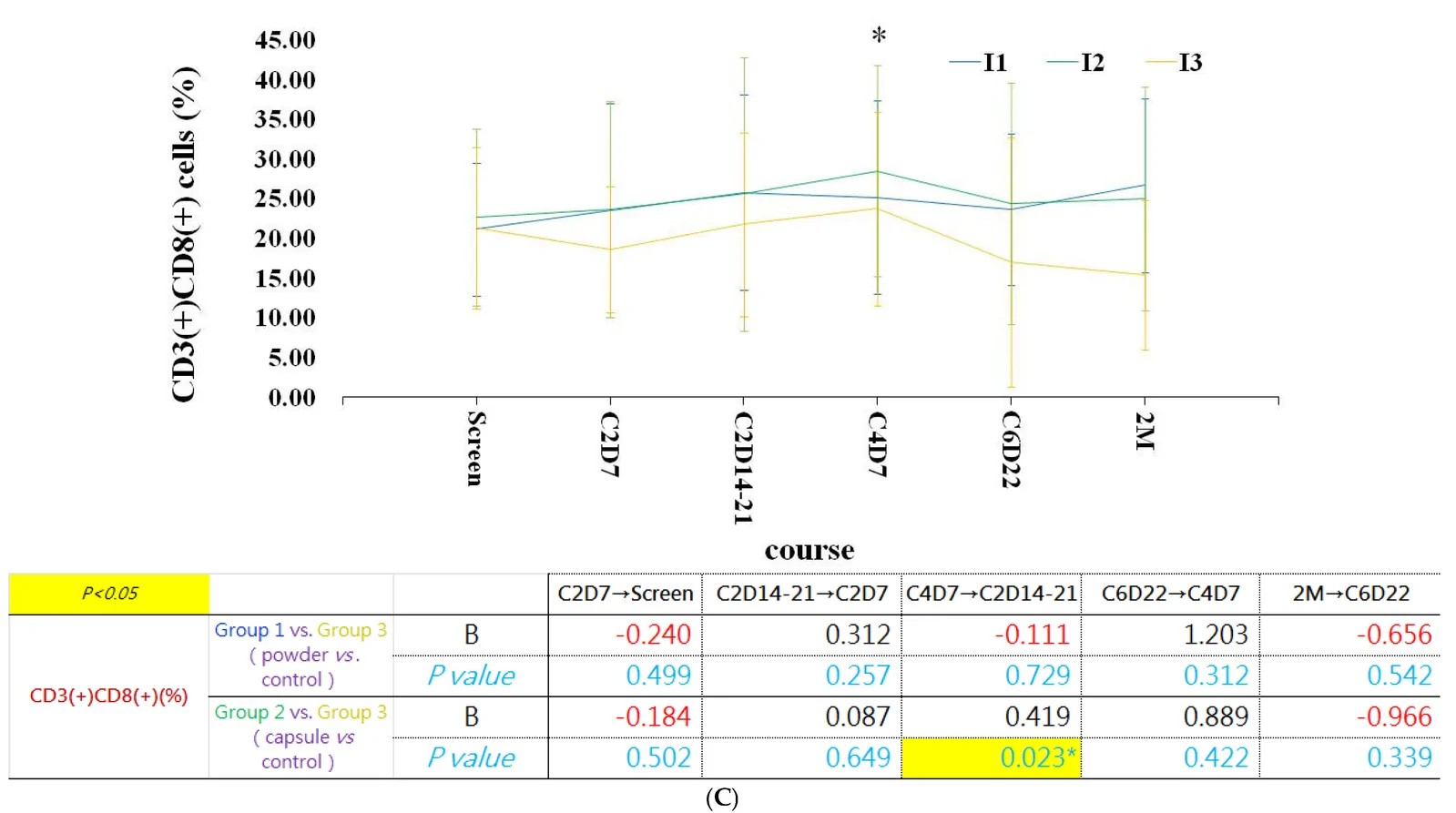
The percentage of T cells between 3 groups. (**A**) CD3 (+) cell expression. (**B**) CD3(+)CD4(+) helper T cells expression. (**C**) CD3(+)CD8(+) killer T cells expression. The percentages of T-cell subsets increased and remained more stable after two cycles of β-glucan supplementation than in the control group. Yeast-derived β-glucan supplementation was initiated on day 7 of cycle 2 of systemic chemotherapy. The blue, green and yellow lines represent group 1 (powder), 2 (capsule) and 3 (control). Data are expressed as mean ± SD. * *p* < 0.05.

The percentages of CD3(+)CD8(+) cytotoxic T cells and CD3(+)CD4(+) helper T cells increased and remained stable after two cycles of β-glucan supplementation during chemotherapy (Figure 5A–C). In contrast, T-cell populations gradually decreased in patients who did not receive β-glucan supplementation during chemotherapy.

#### 2.5.2. β-Glucan Suppressed PD-1 Upregulation During Chemotherapy

**Figure 6:**
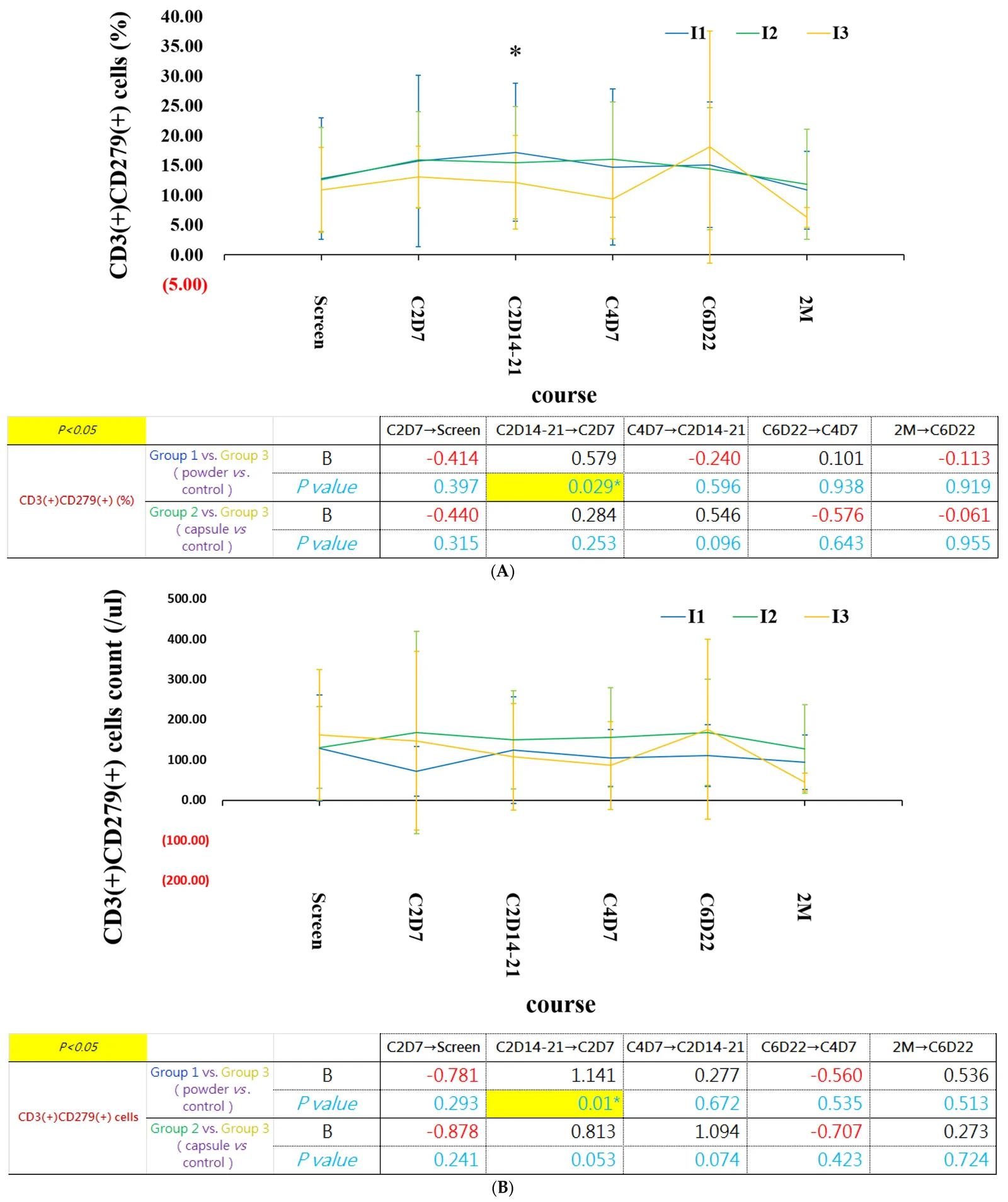

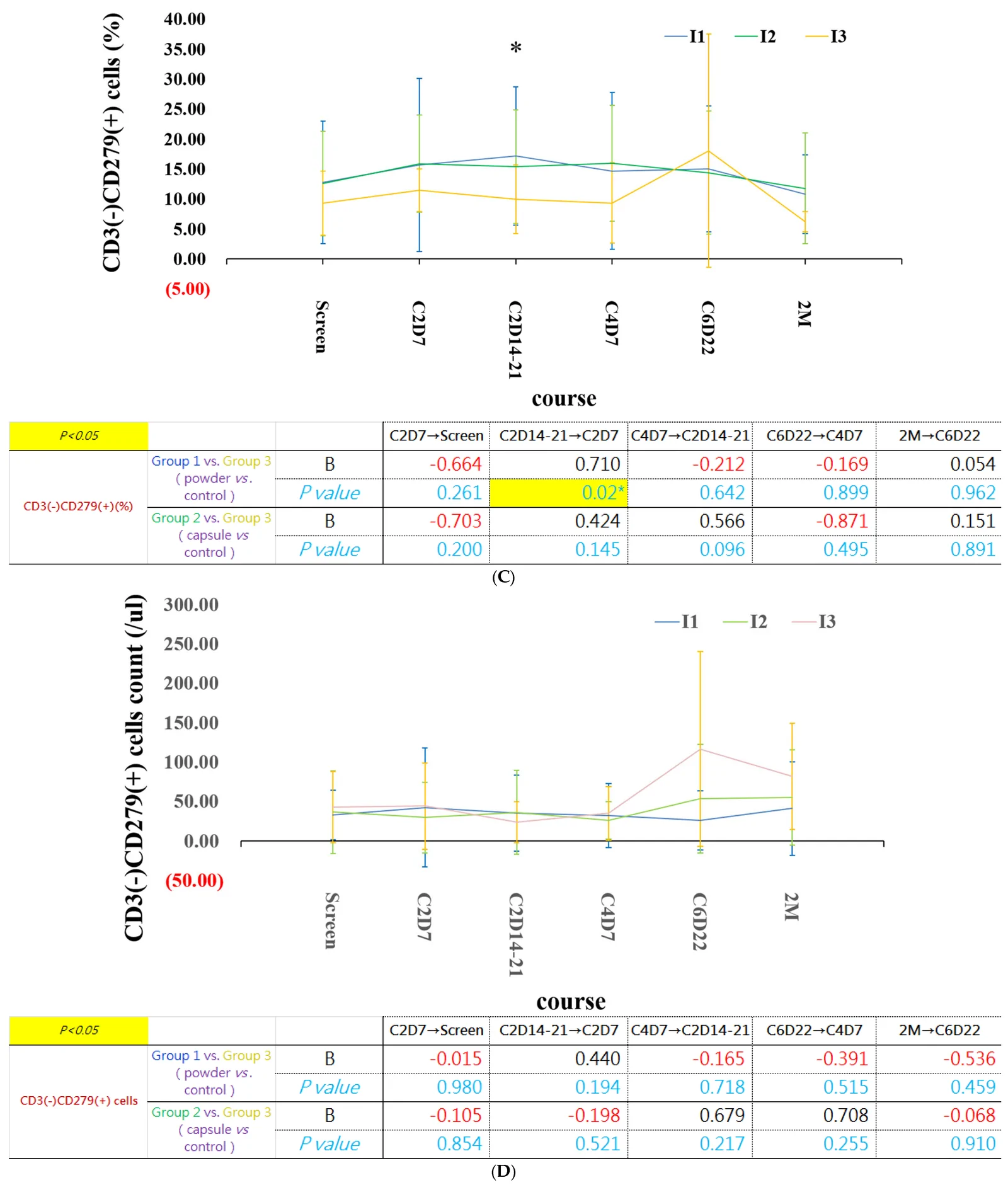
PD-1 (CD279)(+) cell populations were upregulated in the control group after four cycles of chemotherapy, whereas they remained more stable in the β-glucan intervention groups. (**A**) the percentage of CD3(+)CD279(+) cells; (**B**) CD3(+)CD279(+) cell counts; (**C**) the percentage of CD3(-)CD279(+) cells; (**D**) CD3(-)CD279(+) cell counts. Yeast-derived β-glucan supplementation was initiated on day 7 of cycle 2 of systemic chemotherapy. The blue, green and yellow lines represent group 1 (powder), 2 (capsule) and 3 (control). Data are expressed as mean ± SD. * *p* < 0.05.

The percentage of B lymphocytes remained more stable in patients receiving β-glucan supplementation (Supplementary Figure S8). With continued β-glucan supplementation, CD279 (PD-1)(+) cell populations remained more stable and gradually declined over time. Although no significant differences in CD3(+)PD-1 (+) T cells and CD3(-)PD-1 (+) cells among the 3 groups, the percentage of PD-1 (+) non-T cells increased significantly at the 6th cycle of chemotherapy in control group, which may reflect the immunomodulatory effects of β-glucan (Figure 6).

#### 2.5.3. Beta-Glucan Stabilized CD3(+)CD28(+) T Cells and CD3(−)CD28(+) Cells

Ligation of the CD28 receptor on T cells provides a critical second signal alongside T cell receptor (TCR) ligation for naïve T cell activation. CD28 is critical for regulatory T cell survival and the maintenance of immune homeostasis. The effects of CD28 ligation depend on the target T-cell population. Although CD28 ligation promotes the proliferation and effector functions of conventional T cells, it also enhances the anti-inflammatory functions of regulatory T (Treg) cells [13].

Although CD28(+) cells accounted for only a small proportion of lymphocytes (<5%), it provides the critical co-stimulatory signal (Signal 2) required for full T cell activation, survival, and proliferation [14]. CD3(+)CD28(+) T cells are central to the adaptive immune response, serving as fully capable, uninhibited T lymphocytes [15]. CD3(-)CD28(+) cells could be considered to represent NK cells [16]. These cells can receive specific immunomodulatory co-stimulatory signals through interactions with CD80- or CD86-expressing cells [13] and participate in innate immunity by targeting virus-infected cells and tumor cells without prior sensitization [17,18]. In our study, patients receiving β-glucan supplementation exhibited greater stability of both CD3(+)CD28(+) and CD3(-)CD28(+) cell populations throughout chemotherapy compared with the control group. Although CD3(-)CD28(+) cells showed a trend toward upregulation after several cycles of chemotherapy in the control group, this difference did not reach statistical significance (Supplementary Figure S9).

### 2.6. β-Glucan Preserved NK-Cell Homeostasis During Chemotherapy, Including NK-Cell Populations and the KIR Receptor Repertoire

#### 2.6.1. Beta-Glucan Stabilized NK and NK-T Cells

The percentages of NK-cell populations, including CD3(−)CD16(+) and CD3(-)CD56(+) cells, remained more stable in patients receiving β-glucan supplementation than in the control group (Supplementary Figure S10A–D). In the control group, CD3(+)CD56(+) NK-T cell populations showed progressive upregulation during chemotherapy. However, these cell populations accounted for only a small proportion of lymphocytes and remained more stable in the β-glucan supplementation groups. These differences did not reach statistical significance (Supplementary Figure S10E,F).

#### 2.6.2. β-Glucan Stabilized CD3(-)CD158(+) NK Cells and CD3(+)CD158(+) NK-T Cells

**Figure 7:**
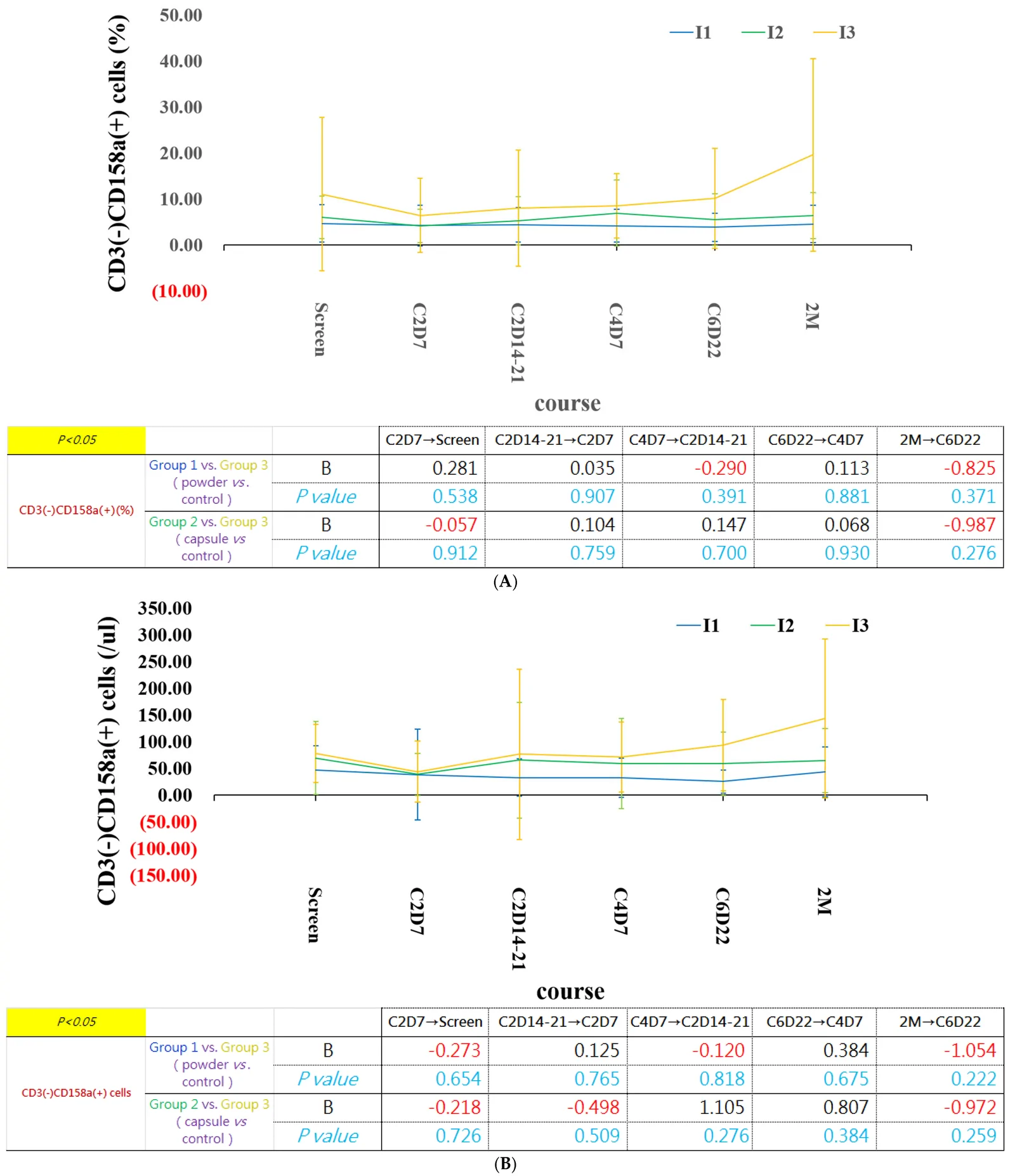

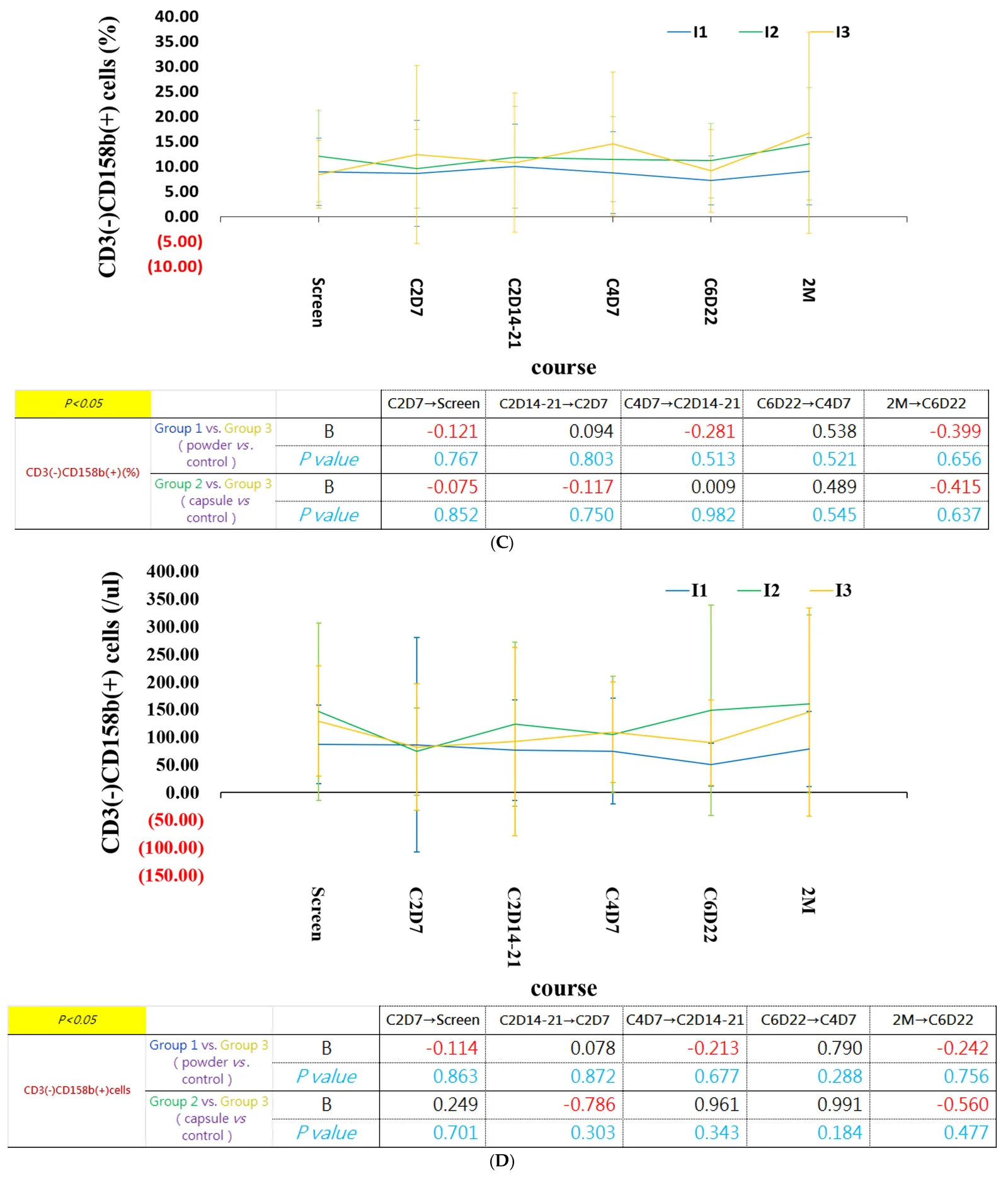

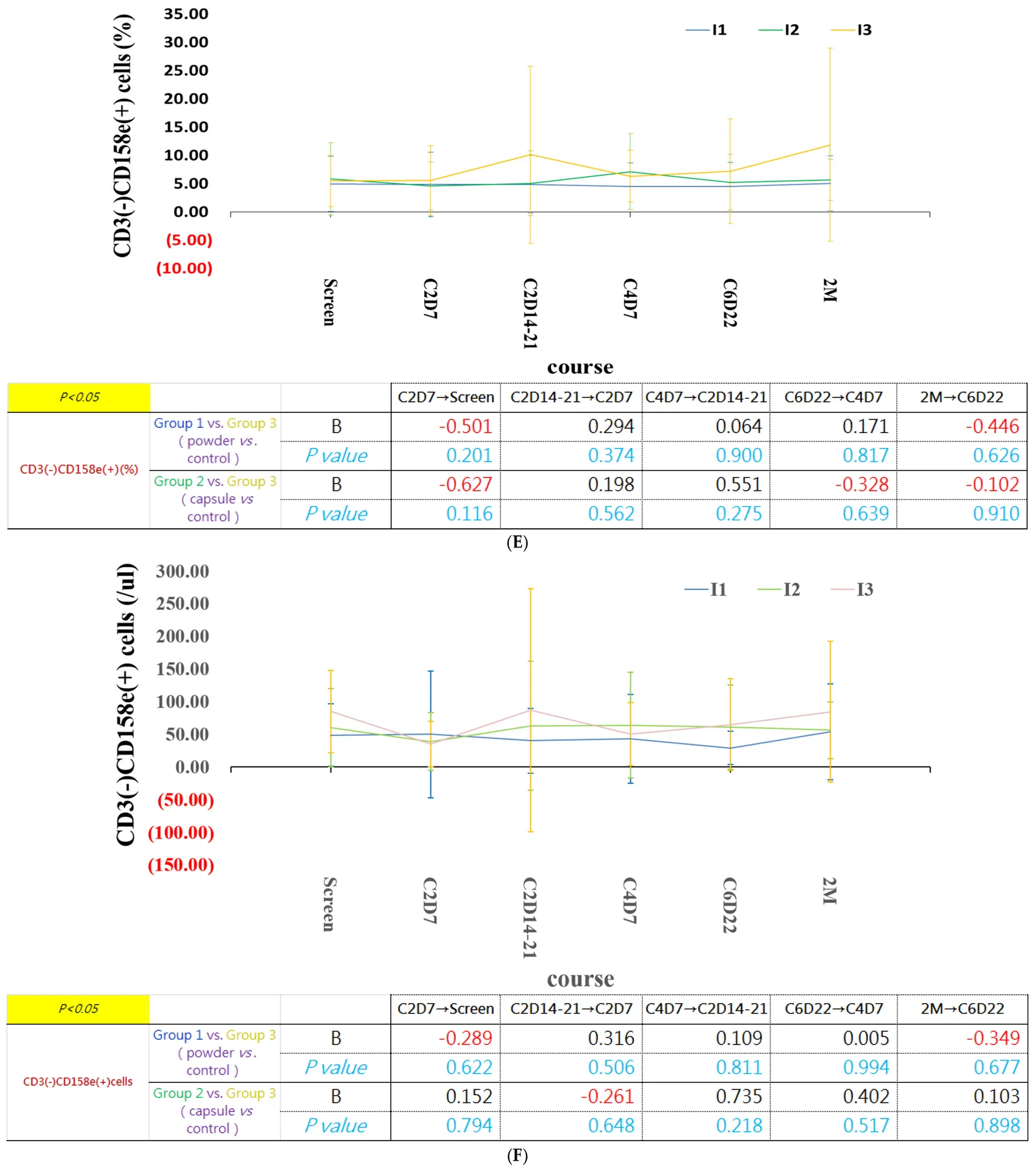

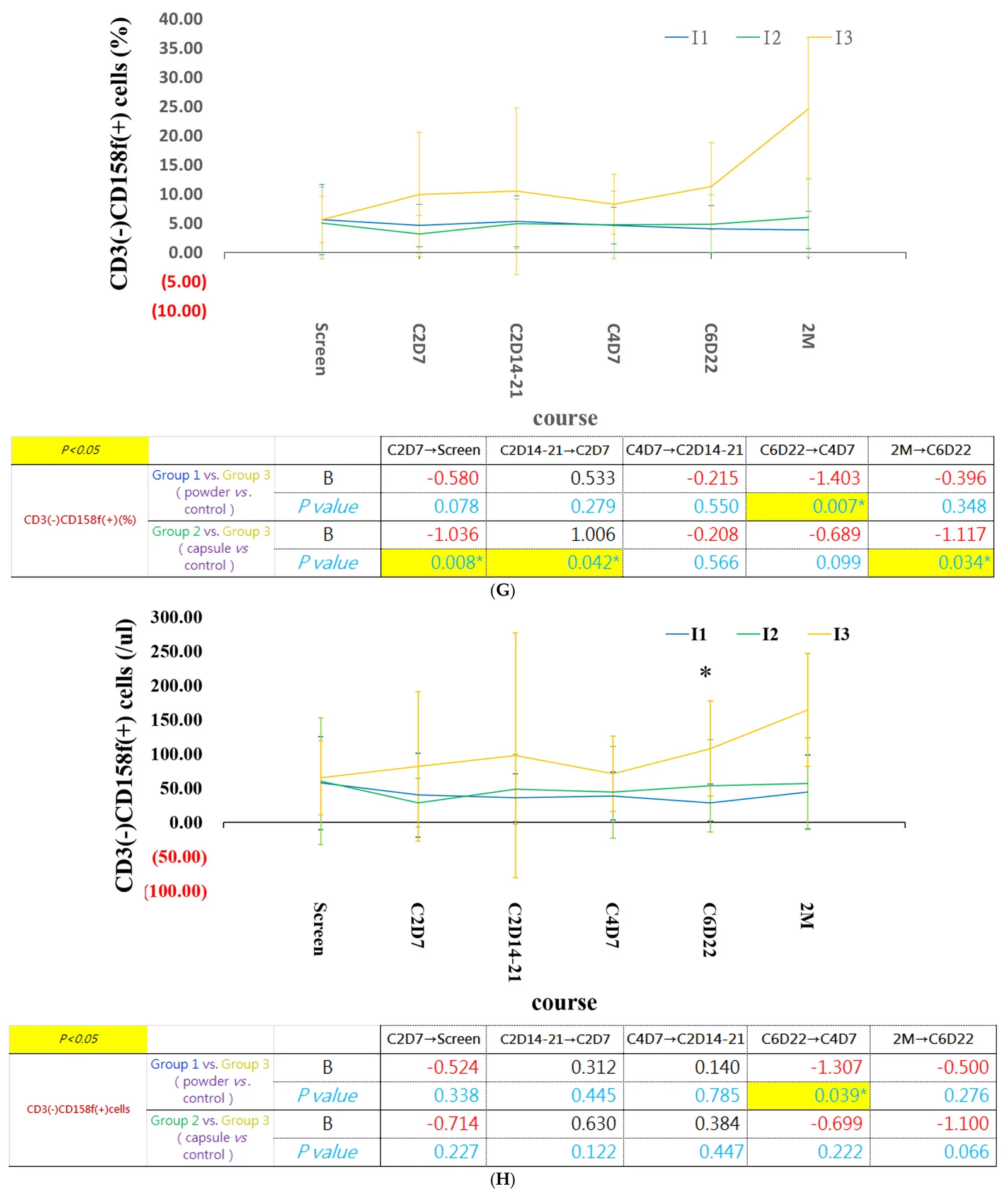

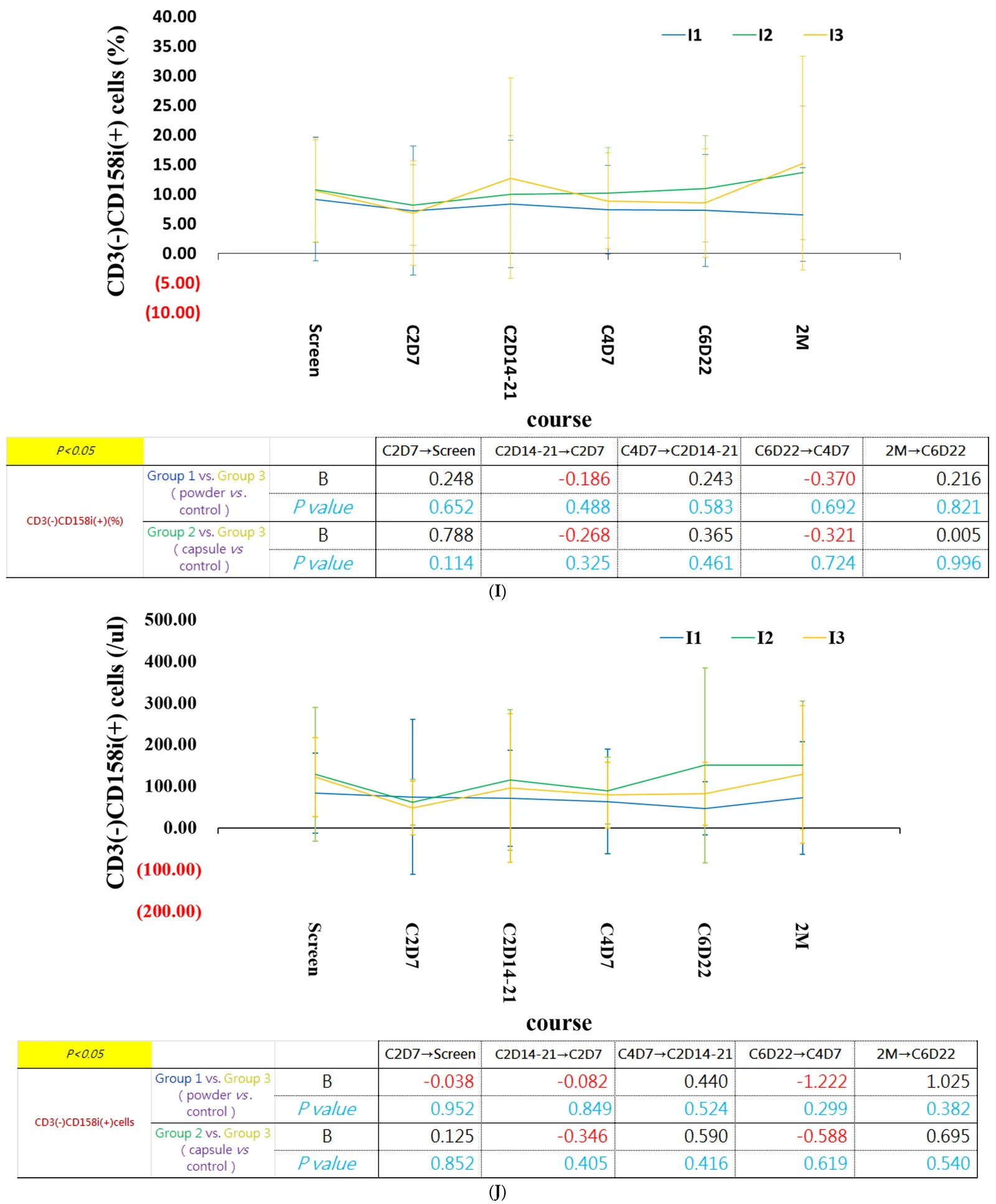
Longitudinal changes in CD3(-)CD158(+) NK-cell subsets in the three study groups. (**A**,**C**,**E**,**G**,**I**)—the percentage of CD3(-)CD158 (+) cells; (**B**,**D**,**F**,**H**,**J**)—the CD3(-)CD158(+) cell count. The percentages of CD3(-)CD158-positive NK-cell subsets remained more stable in the β-glucan supplementation groups throughout chemotherapy. Yeast-derived β-glucan supplementation was initiated on day 7 of cycle 2 of systemic chemotherapy. The blue, green and yellow lines represent group 1 (powder), 2 (capsule) and 3 (control). Data are expressed as mean ± SD. * *p* < 0.05.

The percentage of CD3(-)CD158a (+) and CD3(-)CD158f(+) NK cells increased gradually after six cycles of chemotherapy. In β-glucan intervention groups, the percentage of CD3(-)CD158(+) cell populations remained more stable. Interestingly, the percentages of CD3(-)CD158b(+) and CD3(-)CD158i(+) NK cells were higher in the capsule group than in the powder group beginning 7 days after supplementation, and the difference gradually became more apparent over time (Figure 7).

**Figure 8:**
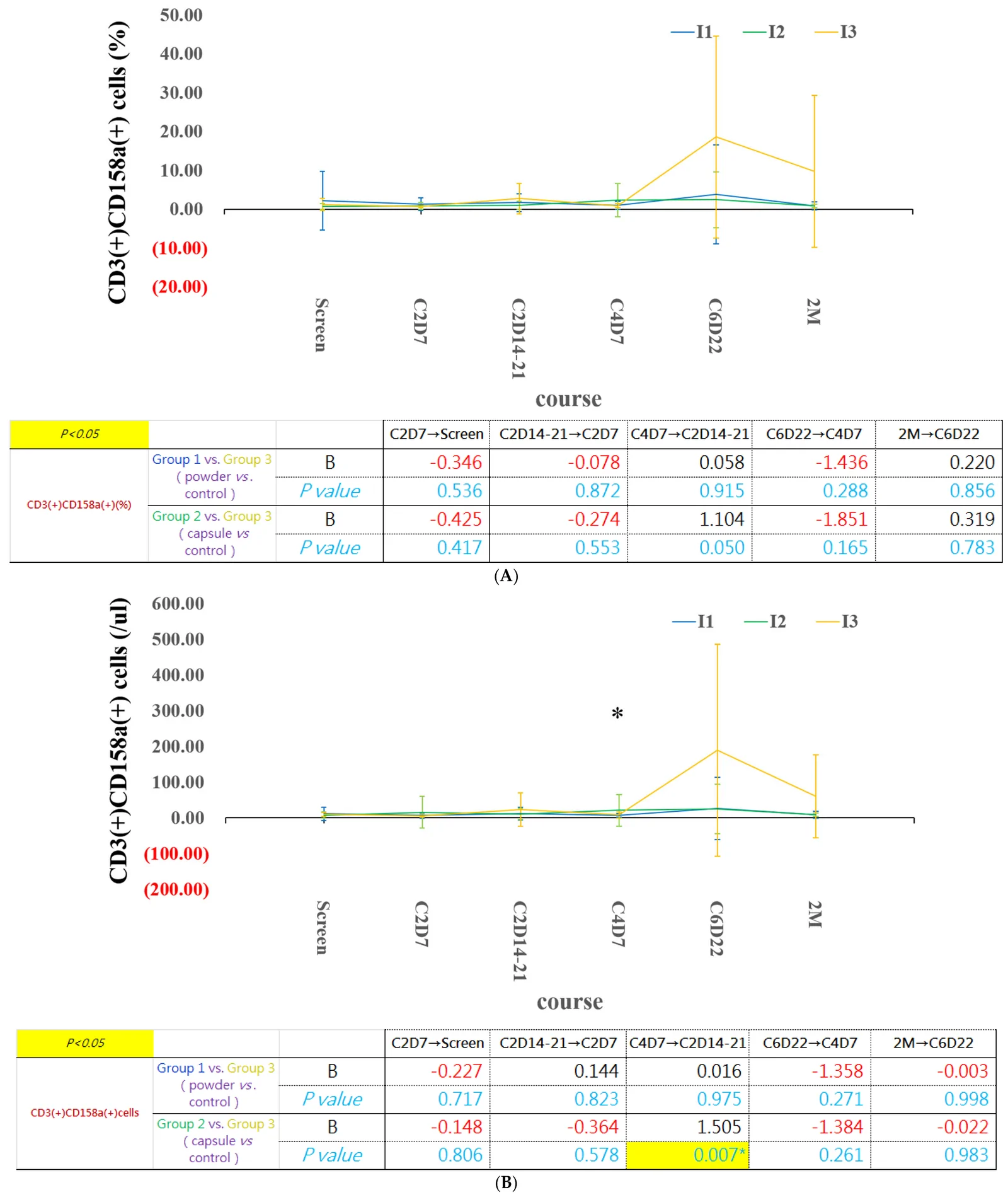

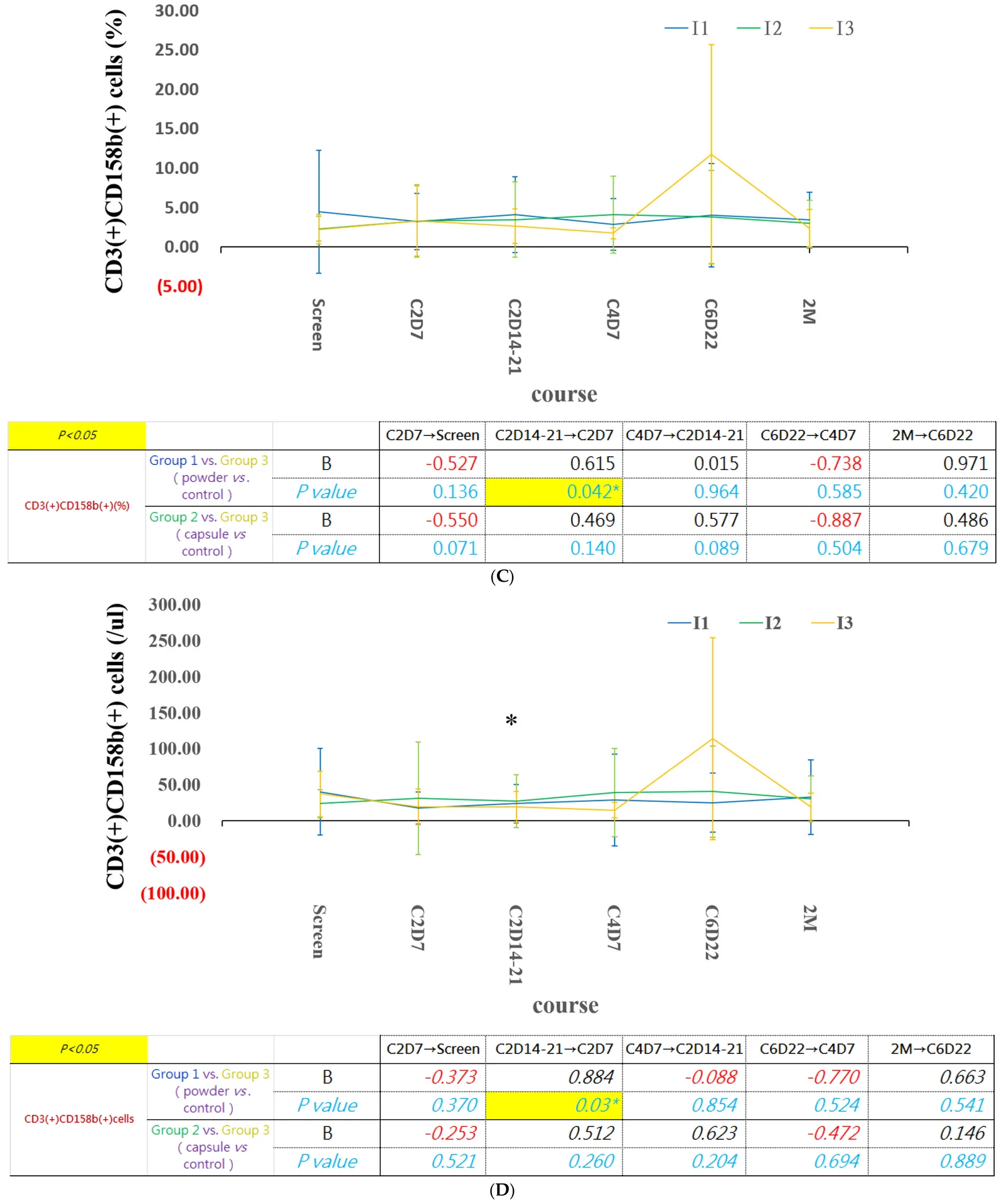

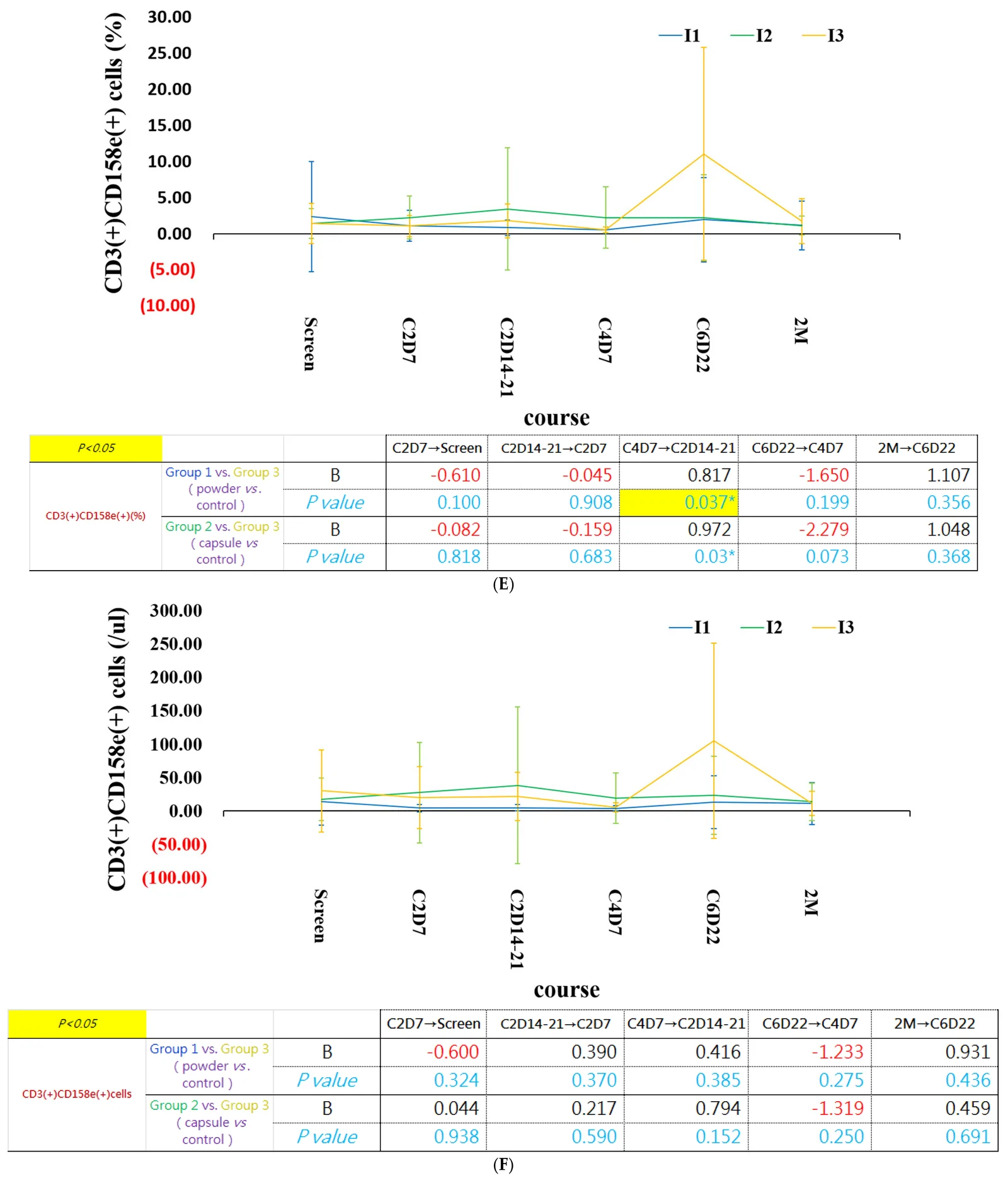

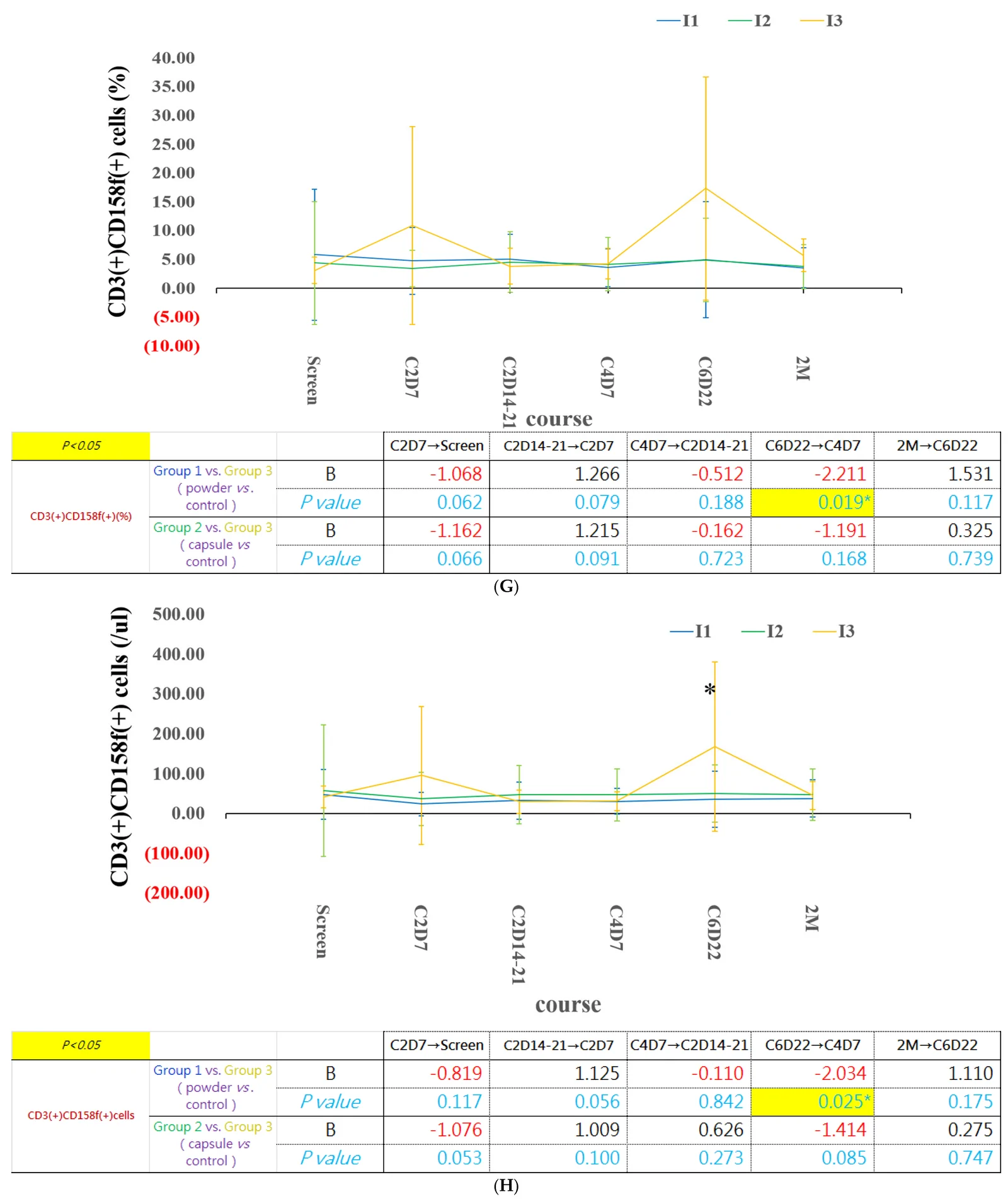

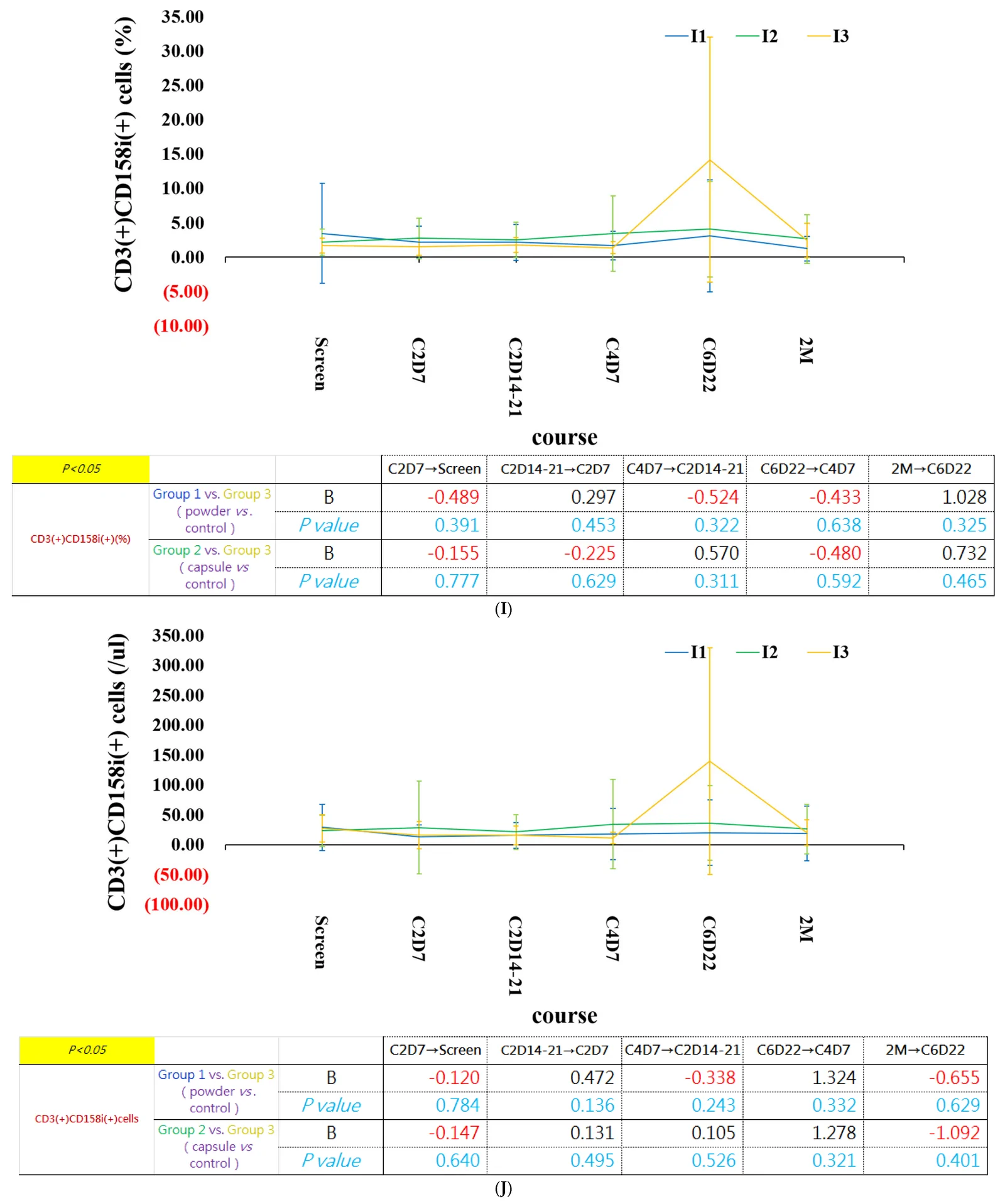
Longitudinal changes in CD3(+)CD158(+) NK-T-cell subsets in the three study groups. (**A**,**C**,**E**,**G**,**I**)—the percentage of CD3(+)CD158 (+) cells; (**B**,**D**,**F**,**H**,**J**)—the CD3(+)CD158(+) cell count. CD3(+)CD158 family-positive NK-T-cell subsets remained more stable in the β-glucan supplementation groups throughout chemotherapy. Yeast-derived β-glucan supplementation was initiated on day 7 of cycle 2 of systemic chemotherapy. The blue, green and yellow lines represent group 1 (powder), 2 (capsule) and 3 (control). Data are expressed as mean ± SD. * *p* < 0.05.

In our study, we also noticed that the percentage of CD3(+)CD158(+) NK-T cells were upregulated, including CD158a, CD158b, CD158e, CD158f, CD158i positive cells, after 6 cycles of chemotherapy, in the control group. In patients with β-glucan intervention groups, the expressions of CD158 family positive cells are stable, without significant change. (Figure 8) However, there was no statistics significant different due to some patients expiring before the latest follow-up time.

Sensitivity analyses using generalized estimating equations (GEEs) yielded findings generally consistent with those obtained from the primary linear mixed-effects models. Significant group-by-time interactions were observed for CD3(-)CD158i(+), CD3(-)CD56(+), CD3(-)CD279(+), and CD3(+)CD20(+) cell subsets, supporting the robustness of the longitudinal immune trajectories (Supplementary Tables S1–S3).

### 2.7. β-Glucan Decreased Jurkat T-Cell Proliferation and Viability

**Figure 9:**
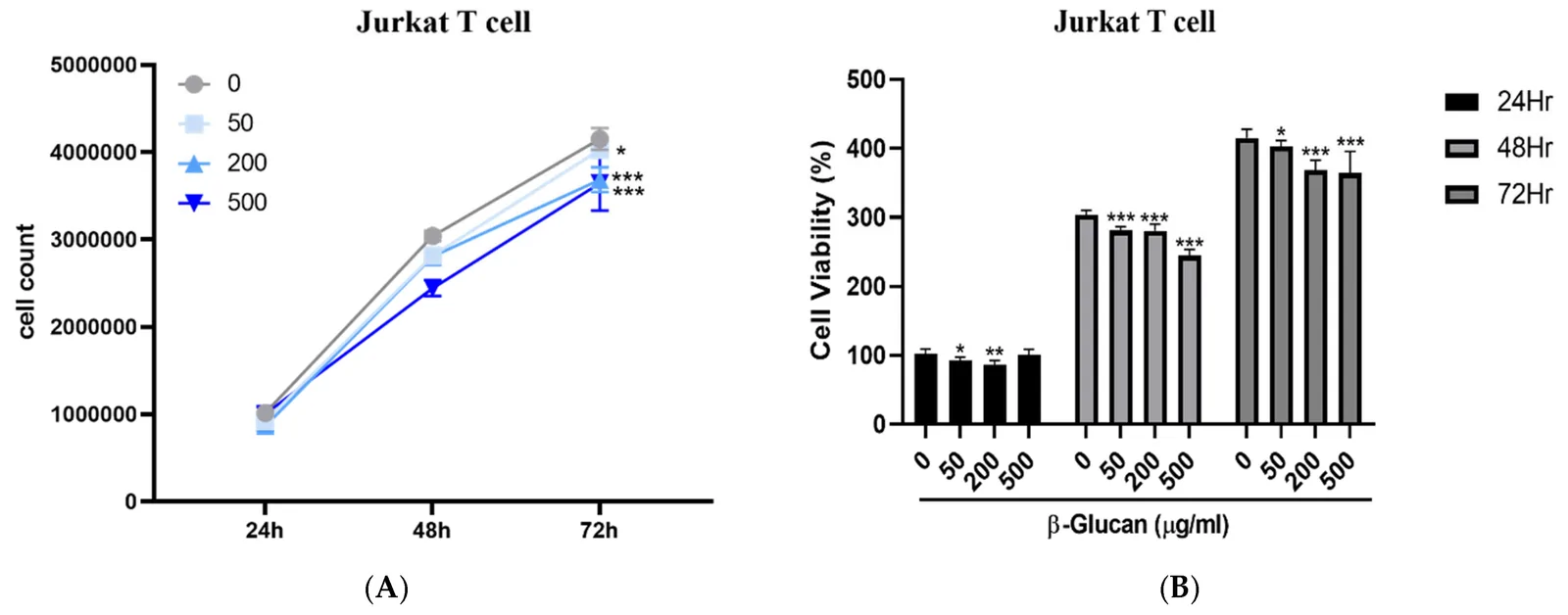
(**A**) Dose-dependent inhibitory effect of β-glucan on Jurkat T-cell proliferation. Jurkat cells were treated with β-glucan at concentrations of 0, 50, 200, and 500 μg/mL for 24, 48, and 72 h. (**B**) Cell viability was determined using the MTT assay. Data are presented as mean ± SD. * *p* < 0.05 ** *p* < 0.01 and *** *p* < 0.001 compared with the control group (0 μg/mL).

Jurkat T cells cultured in the absence or presence of β-glucan (50, 200, and 500 μg/mL) exhibited progressive proliferation throughout the 72 h observation period (Figure 9A). At 24 h, cell numbers were comparable among all treatment groups. After 48 h, β-glucan-treated cells showed a modest reduction in cell counts compared with the untreated control, with the greatest reduction observed at 500 μg/mL. Although cell numbers continued to increase at 72 h in all groups, those in the β-glucan-treated groups remained lower than those in the untreated control, indicating a modest inhibitory effect on Jurkat cell expansion during prolonged culture. Cell viability, as determined by the MTT assay, demonstrated a similar temporal pattern (Figure 9B). Cell viability increased over time in all groups, reflecting continuous cellular growth. However, compared with untreated cells, β-glucan treatment resulted in significantly lower metabolic activity at 48 and 72 h. The reduction was most evident at the highest concentration (500 μg/mL). These findings suggest that prolonged exposure to β-glucan modestly reduced the metabolic activity of Jurkat T cells in a concentration- and time-dependent manner.

## 3. Discussion

Systemic chemotherapy remains one of the most important treatments for metastatic cancer patients. Cytotoxic chemotherapy-induced leukopenia and neutropenia may be associated with a higher infection rate and delay in treatment. β-glucan has been shown to improve hematopoiesis, enhance neutrophil chemotaxis and adhesion, and partially ameliorate chemotherapy-induced cytopenia in patients with advanced malignancies receiving chemotherapy [19]. In our prospective study, patients receiving either β-glucan powder or capsules maintained higher leukocyte and neutrophil counts after 3–4 weeks of supplementation than those in the control group. Although no significant survival difference was observed, treatment response rates were higher in the β-glucan groups, particularly in the β-glucan powder group.

N/L ratio (NLR) has a well-known prognostic value and independently correlates with mortality in the general population and in several specific subsets of disease, including cancer [20]. Templeton et al. [21] demonstrated that NLR higher than 4 independently predicts a shorter overall survival across multiple tumor types (HR 1.81, 95% CI = 1.67 to 1.97; *p* < 0.001). NLR has also been reported as a marker of tumor progression in hepatocarcinoma [22]. In our study, the NLR decreased after 6–8 weeks of β-glucan supplementation and remained stable after the fifth treatment cycle in the β-glucan groups. Patients receiving β-glucan powder exhibited a lower and more stable NLR than those receiving capsules, which may predict more favorable clinical outcomes.

Cancer immune surveillance is an essential host defense mechanism that inhibits carcinogenesis and maintains cellular homeostasis. Cancer immunoediting consists of three sequential phases: elimination, equilibrium, and escape [1]. Over the past decade, immune checkpoint inhibitors have been widely used to treat a variety of cancers. In addition to cytotoxic effects, one of the mechanisms of chemotherapy is to provoke the immune system to recognize and destroy malignant cells, called immunogenic cell death (ICD) [23]. However, in mice study, only anthracyclines provide enhanced immunity [24]. β-Glucans interact with several immune receptors, including Dectin-1, complement receptor 3 (CR3), and TLR-2/6, thereby activating multiple immune-cell populations, including macrophages, neutrophils, monocytes, natural killer cells, and dendritic cells [25]. Woeste et al. reported that dietary β-glucan supplementation decreased PD-1(+)CD8(+) T cells in a murine pancreatic cancer model [26]. Consistent with these findings, we observed increased CD8(+) cytotoxic T-cell populations and reduced PD-1(+) cell populations following β-glucan supplementation. These findings suggest that β-glucan may enhance both the abundance and function of cytotoxic T cells, thereby contributing to antitumor immunity.

Our findings suggest that beta-glucan supplementation may not simply increase peripheral immune-cell numbers, but may help preserve immune homeostasis during chemotherapy by attenuating excessive fluctuations in NK-cell and T-cell phenotypes.

Interestingly, β-glucan supplementation preserved relatively stable levels of both CD3(+)CD28(+) and CD3(-)CD28(+) cells throughout chemotherapy, whereas the control group showed greater fluctuations, including a trend toward increased CD3(-)CD28(+) cells after repeated chemotherapy cycles, although this did not reach statistical significance. CD28 is a critical co-stimulatory receptor that regulates T-cell activation, proliferation, and survival through CD80/CD86 signaling, while CD28 expression on CD3(-) cells has also been reported in activated NK-cell subsets, where it may contribute to cytokine production and immune regulation [13]. Therefore, the preserved stability of CD28-positive cell populations in the β-glucan groups may indicate preservation of both adaptive and innate immune homeostasis during chemotherapy. One of the novel findings of this study was that β-glucan supplementation preserved the longitudinal stability of both NK/NKT-cell populations and the KIR (CD158) receptor repertoire throughout chemotherapy. Although these differences did not reach statistical significance, patients receiving β-glucan demonstrated relatively stable CD3(-)CD16(+) NK cells, CD3(-)CD56(+) NK cells, CD3(+)CD56(+) NKT cells, and CD158-positive NK/NKT subsets, whereas the control group exhibited gradual upregulation of CD3(-)CD158a(+), CD3(-)CD158f(+), and multiple CD3(+)CD158-positive NKT-cell populations after repeated chemotherapy cycles. Because inhibitory KIRs are essential for NK-cell education and functional competence rather than simply suppressing NK-cell activity [27,28,29], these findings may reflect chemotherapy-induced KIR repertoire remodeling and compensatory immune adaptation, while β-glucan appears to attenuate this process by preserving innate immune homeostasis [25]. Interestingly, although both β-glucan formulations maintained overall KIR homeostasis, the capsule formulation was associated with relatively higher proportions of CD158b(+) and CD158i(+) NK cells than the powder formulation during the later stages of treatment. Because CD158b and CD158i represent inhibitory and activating KIR receptors, respectively, these findings may reflect formulation-dependent differences in maintaining the balance of KIR receptor expression rather than simply enhancing or suppressing NK-cell activity.

Two potential mechanisms may explain these observations. First, β-glucan directly activates innate immune cells through Dectin-1 and complement receptor 3 (CR3), leading to Syk–CARD9–NF-κB signaling, trained immunity, enhanced dendritic cell maturation, and increased IL-12-, IL-15-, and IL-18-mediated NK-cell activation and survival [25,30]. These pathways may contribute to preserving NK-cell numbers and maintaining KIR receptor homeostasis during repeated chemotherapy. Second, β-glucan may indirectly regulate NK-cell immunity through modulation of the gut microbiota [31,32,33]. As a fermentable dietary polysaccharide, β-glucan promotes beneficial bacterial taxa and increases short-chain fatty acid production, thereby preserving intestinal barrier integrity, reducing systemic inflammation, and improving host immune homeostasis. This gut–immune axis may further contribute to the stabilization of NK/NKT-cell populations and their KIR receptor repertoire during chemotherapy. Interestingly, the capsule formulation (β-glucan plus L-glutamine) maintained relatively higher proportions of CD158b(+) and CD158i(+) NK cells than the powder formulation, suggesting that the two different formulations may differentially modulate inhibitory and activating KIR subsets. Further mechanistic studies integrating cytokine profiling, NK-cell functional assays, KIR genotyping, and gut microbiome analyses are warranted to validate these hypotheses.

In our in vitro study, β-glucan did not enhance Jurkat T-cell proliferation. Instead, prolonged exposure, particularly at higher concentrations, modestly reduced cell numbers and metabolic activity in a time-dependent manner. Since Jurkat cells represent leukemic T lymphoblasts rather than normal primary T cells, these findings suggest that β-glucan is unlikely to function as a nonspecific mitogen. Instead, β-glucan may exert selective immunomodulatory effects while modestly suppressing the proliferation of transformed T cells. This observation is consistent with our clinical findings showing preservation of peripheral immune cell populations without evidence of uncontrolled lymphocyte expansion, supporting the concept that β-glucan acts as an immune regulator rather than a direct proliferative stimulus.

This study has several limitations. First, the study population consisted of patients with advanced-stage solid tumors receiving systemic chemotherapy, many of whom had a limited life expectancy. Consequently, a proportion of participants, particularly in the control group, died before completing the scheduled serial immune assessments. This inevitable attrition reduced the availability of longitudinal immune profiling data and may have introduced survivor bias, particularly in the later time-point analyses. Second, this was a single-center prospective randomized phase II trial with a relatively modest sample size and heterogeneous cancer types, which may limit the generalizability of the findings. Third, although comprehensive longitudinal immune profiling and complementary in vitro T-cell experiments were performed, detailed mechanistic investigations, including cytokine profiling, functional immune assays, transcriptomic analyses, and gut microbiome characterization, were not included in the present study. Finally, the two β-glucan formulations differed not only in dosage form but also in accompanying nutritional components. Therefore, the individual contributions of β-glucan and the additional nutritional ingredients could not be completely distinguished. Future multicenter randomized studies with larger cohorts and integrated mechanistic investigations are warranted to validate these findings. Nevertheless, this limitation reflects the real-world clinical characteristics of patients with advanced malignancies undergoing chemotherapy. Despite unavoidable attrition, longitudinal immune profiling consistently demonstrated that β-glucan supplementation preserved immune homeostasis throughout chemotherapy, supporting the robustness of the observed immunomodulatory effects.

## 4. Materials and Methods

### 4.1. Patients

This study is an open-label, randomized prospective clinical trial. The inclusion criteria were as follows: stage IV adult cancer patients (age ≥ 20 years) who were scheduled to receive systemic cytotoxic chemotherapy, including cisplatin, carboplatin, oxaliplatin, irinotecan/topotecan, taxanes, fluorouracil, alkylating agents, with an Eastern Cooperative Oncology Group (ECOG) performance status of 0 or 1. Exclusion criteria included previous systemic chemotherapy or large-field radiotherapy within 6 months before enrollment, early-stage cancer, liver dysfunction unrelated to cancer (AST > 2 × ULN or ALT > 2 × ULN), renal dysfunction not correctable by hydration (serum creatinine > 2 mg/dL), known allergy to β-glucan, and age < 20 years.

**Figure 10:**
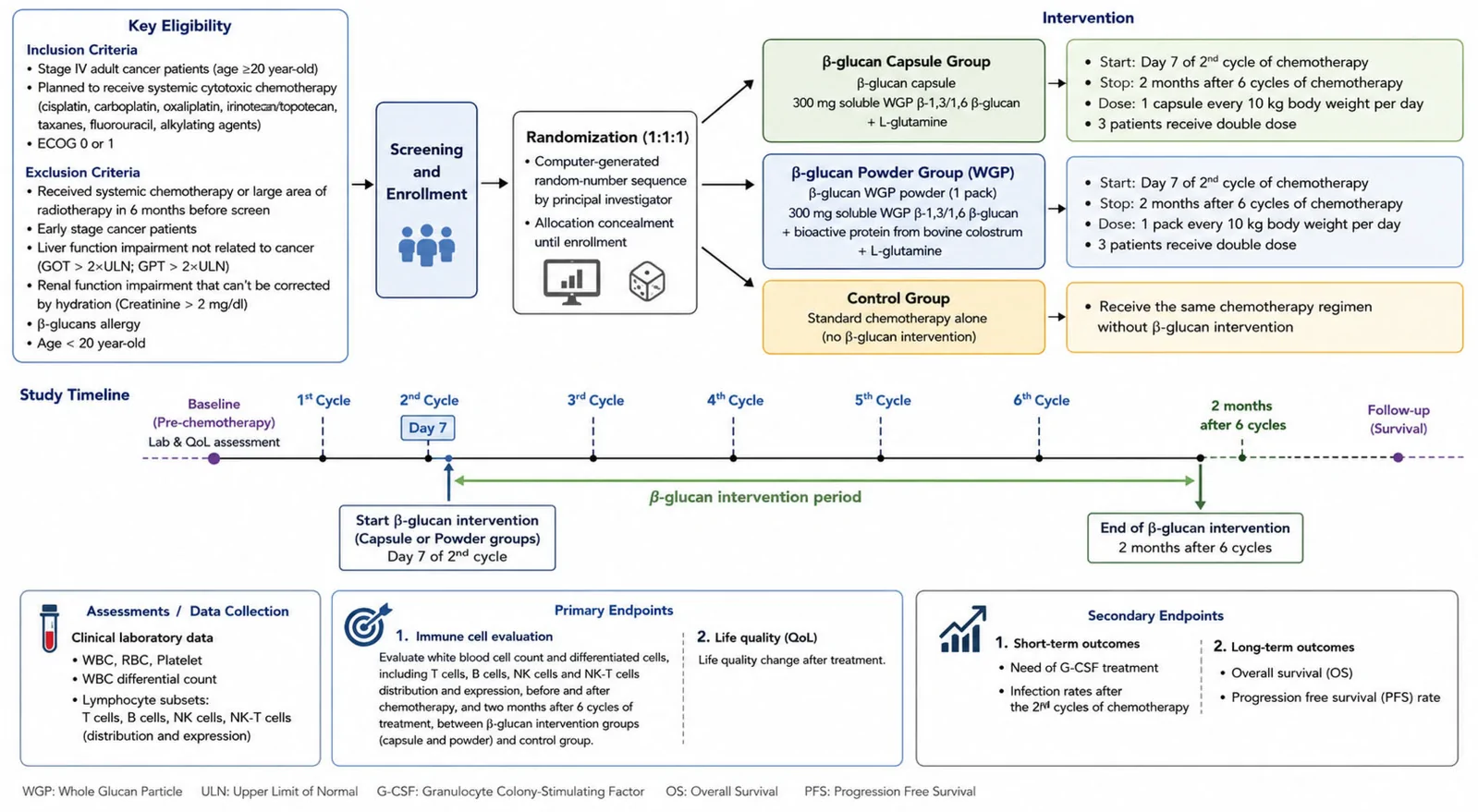
The diagram for the treatment schedule of our study.

The randomization sequence was generated using a computer-generated randomization sequence by the principal investigator. Eligible participants were enrolled by the study investigators and assigned to one of the three intervention groups, in a 1:1:1 ratio, according to the predefined randomization sequence. Allocation was concealed until participant enrollment according to the predefined computer-generated randomization sequence. Patients with yeast-derived β-glucans intervention were started on the 7th day of 2nd cycle of systemic chemotherapy and stopped at 2 months after 6 cycles of systemic chemotherapy. Participants received one capsule or one sachet per 10 kg of body weight daily. Three patients in each treatment group received a double dose of β-glucans. The β-glucan WGP powder contains 300 mg soluble WGP β-1,3/1,6 β-glucan, bioactive protein from bovine colostrum and L-glutamine. The β-glucan capsule contains 300 mg soluble WGP β-1,3/1,6 β-glucan and L-glutamine. Clinical laboratory parameters were collected, including white blood cell count (WBC), red blood cell (RBC), platelet, and WBC differential count. The primary endpoints of this clinical trial were (1) longitudinal changes in the white blood cells count and differentiated cells, including the distributions and expressions of T cells, B cells, NK cells, and NK-T cells, before and after chemotherapy, and two months after six cycles of treatment, between β-glucan intervention groups (capsule and powder) and control group; (2) changes in quality of life after treatment. The secondary endpoints included the need for G-CSF support, infection rate after the second chemotherapy cycle, overall survival, and progression-free survival. The diagram for the treatment schedule is shown in Figure 10.

This trial was approved by the Institutional Review Board of the E-Da Hospital with the number EMRP50106N. This study was registered at ClinicalTrials.gov with the number NCT04710290.

### 4.2. Flow Cytometry

Flow cytometry was performed to evaluate immune-cell subsets and CD markers including NK cells, helper and killer T cells, Treg cells, PD-1 expression T cells and CD158 family (BD Pharmingen™ PE Mouse Anti-Human CD279, FITC Mouse Anti-Human CD3, BD Pharmingen™ PE Mouse Anti-Human CD4, BD Pharmingen™ PerCP-Cy5.5 Mouse Anti-Human CD8, BD Pharmingen™ PE Mouse Anti-Human CD28, BD Pharmingen™ PE Mouse Anti-Human NKB1, BD Pharmingen™ Mouse Anti-Human CD20 PE-Cy™, BD Pharmingen™ PerCP-Cy™5.5 Mouse Anti-Human CD16, PerCP-Cy5.5 Mouse Anti-Human CD56, BD Pharmingen™ PE Mouse Anti-Human CD158b [BD Biosciences, San Jose, CA, USA], BECKMAN CD158a, h-PE, BECKMAN CD158F-RPE, BECKMAN CD158i-PE [Beckman Coulter, Marseille, France]) were used. Samples were analyzed using a BD FACSCalibur™ flow cytometer (San Jose, CA, USA).

### 4.3. Cell Culture and Reagent

The human T lymphocyte Jurkat cell line was obtained from the Bioresource Collection and Research Center (BCRC, Hsinchu, Taiwan). The cells were cultured in RPMI 1640 medium (Gibco, Waltham, MA, USA) supplemented with 10% fetal bovine serum (Gibco, Waltham, MA, USA). Cell density was maintained below $3\times 10^{6}$ cells/mL, with fresh medium replenished 2 to 3 times per week. For preparation of the beta-glucan and L-glutamine, the contents of the capsule were emptied and dissolved in sterile water. The solution was then filtered through a 0.22-μm membrane filter for sterilization and stored at −20 °C until use.

### 4.4. Cell Viability Analysis

The Jurkat cell line, derived from a case of T-cell lymphoblastic leukemia, was seeded in a 96-well plate in quadruplicate at 20,000 cells/well and cultured for 24 h before treatment. Cell viability was analyzed by Cell Counting Kit-8 (Sigma-Aldrich, St. Louis, MO, USA; Merck KGaA, Darmstadt, Germany) and measured the absorbance at 450 nm using a microplate reader.

### 4.5. Statistics

ANOVA was used to analyze age and G-CSF use between the three groups. Kaplan–Meier survival analysis was used for overall survival analysis. We used a chi-square test for analysis of sex distribution and cancer types between different groups. SPSS 18.0 (SPSS, Inc.; Chicago, IL, USA) was used to perform the statistical analyses. Repeated-measures linear mixed-effects models were used with generalized linear mixed models [34,35,36] to analysis the change between different time series of white blood cell (WBC), WBC differential count, including neutrophil, lymphocyte, monocyte, eosinophil, and basophil, hemoglobin (Hb), and platelet, in three groups. We also used the same model for analysis the change in different leukocyte surface marker levels in the three groups’ participates. The models included treatment group, time point, and group-by-time interaction as fixed effects, with participates identifier included as a random intercept. The group-by-time interaction term was used to determine whether longitudinal trajectories differed among the powder, capsule, and control groups. All available observations were included without imputation of missing values. Because not all patients had available 2-month follow-up data, analyses involving the 2-month time point were interpreted as available-case analyses.

As a sensitivity analysis, generalized estimating equations (GEEs) were additionally applied to evaluate population-averaged longitudinal effects, using an exchangeable working correlation structure.

A heatmap was generated to visualize the baseline distribution of selected hematologic parameters and N/L ratio among the three study groups. Marker values were standardized using z-score transformation to eliminate differences in measurement scales, and the normalized values were displayed as a color-coded matrix. The heatmap was used as an exploratory visualization to compare the overall baseline immune profiles among treatment groups and was not used for statistical inference.

## 5. Conclusions

β-Glucan supplementation may attenuate chemotherapy-induced immune remodeling by preserving immune homeostasis in patients with advanced solid tumors. β-Glucan supplementation was associated with accelerated neutrophil recovery; stabilization of the neutrophil-to-lymphocyte ratio; and preservation of T-cell, NK-cell, NKT-cell, and KIR (CD158) receptor homeostasis. It was also associated with an improved treatment response. These findings support further investigation of β-glucan as an adjunctive immunonutritional intervention during chemotherapy.

## Figures and Tables

**Table 1:** Patients’ characteristics.

Group ^#^	Group 1 (n = 32)		Group 2 (n = 30)			Group 3 (n = 34)	*p* Value
**Age (mean ± SD)**	60.53 ± 10.565		59.53 ± 8.693			58.47 ± 11.511	0.723
**Sex (M/F)**	17/15		23/7			26/4	0.002 *
**Cancer type** Ampula vactar cancer Appendix cancer Bladder cancer Breast cancer Cervical cancer Colon cancer Esophageal cancer Gastric cancer HCC Head & neck cancer Intrahepatic cholangiocarcinoma Neuroendocrine cancer NPC Ovarian cancer Pancreatic cancer Primary unknown Thymic carcinoma UCC	1 2 2 0 1 2 11 1 0 2 1 0 1 4 1 0 1 0		0 0 1 2 0 2 11 0 1 1 3 1 0 3 1 1 0 1			1 0 0 0 1 2 22 1 0 0 2 0 0 0 0 0 1 2	0.496
**Response rate** CR PR SD PD Expire ^&^ unknown	1 8 17 4 1 1		3 11 6 7 2 1			2 12 4 9 6 1	0.039 *
**G-CSF use after 2nd course (mean ± SD) ^$^**	1.10 ± 2.413 (n = 31)		1.41 ± 4.940 (n = 27)			1.06 ± 2.407 (n = 19)	0.931
**Infection after 2nd course (%)**	8 (25%)		10 (33.33%)			16 (47%)	0.166
**G-CSF use after 2nd course (mean ± SD) ^$^**	Normal dose (n = 28)	High dose (n = 3)	Normal dose (n = 24)	High dose (n = 3)	1.06 ± 2.407		0.954
	1.07 ± 2.463	1.33 ± 2.309	1.58 ± 5.225	0			

^#^ group 1: powder; group 2: capsule; group 3: control; ^&^ expire before follow response; ^$^ Exclude patients in study group who used compounds for less than 1 week; the dose of G-CSF count started since 2nd course of chemotherapy to complete chemotherapy courses. Exclude patients in control group who expired in 2 cycles of treatment. * *p* < 0.05. Abbreviation: NPC: nasopharyngeal cancer; UCC: urothelial cell carcinoma; CR: complete response; PR: partial response; SD: stable disease; PD: progression disease; G-CSF (Filgrastim).

## Data Availability

The datasets generated and analyzed during the current study are not publicly available because they contain potentially identifiable clinical information and are subject to institutional ethics regulations. De-identified data are available from the corresponding author upon reasonable request and with approval from the Institutional Review Board.

## Supplementary Materials

The following supporting information can be downloaded at: https://www.mdpi.com/article/10.3390/ijms27167189/s1. Reference [37] is cited in the supplementary materials.

## Abbreviations

The following abbreviations are used in this manuscript:

NK	Nature Killer cells
DC	Dendritic cell
PMN	Polymorphonuclear cells
KIR	Killer-cell Immunoglobulin-like Receptors
WBC	White blood cells
Hb	hemoglobin
PLT	platelet
PD-1	Programmed death-1
Treg	Regulatory T cell
NPC	Nasopharyngeal cancer
UCC	Urothelial cell carcinoma
CR	Complete response
PR	Partial response
SD	Stable disease
PD	Progression disease
