# Supplementary material for: Beta-Glucan Supplementation Improves Clinical Outcomes and Preserves Immune Homeostasis in Patients with Advanced Solid Tumors Receiving Chemotherapy: A Prospective Phase II Randomized Three-Arm Clinical Trial
Source: Int J Mol Sci. 2026 Aug 11;27(16):7189. doi: 10.3390/ijms27167189 (PMC13513407; doi:10.3390/ijms27167189)
Supplement: Supplementary file 1 [file ijms-27-07189-s001.zip › Supplementary Figure S8.pdf]

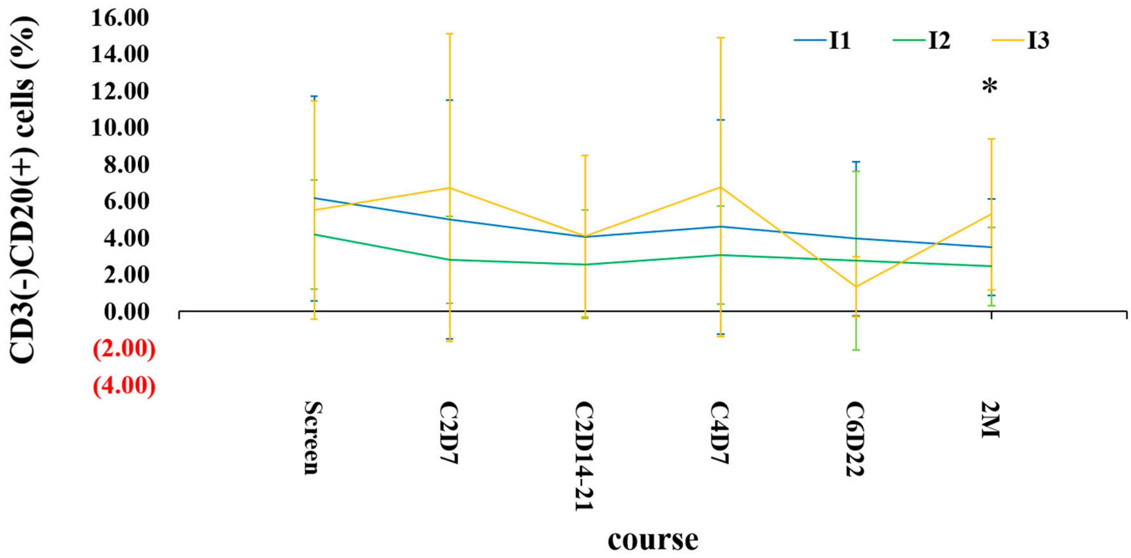

P<0.05			C2D7→Screen	C2D14-21→C2D7	C4D7→C2D14-21	C6D22→C4D7	2M→C6D22
CD3(-)CD20(+)(%)	Group 1 vs. Group 3 (powder *vs*. control)	B	-0.601	0.159	-0.476	0.493	-1.831
		P value	0.433	0.691	0.408	0.536	0.044*
	Group 2 vs. Group 3 (capsule *vs* control)	B	-0.523	0.067	-0.261	0.619	-1.465
		P value	0.491	0.841	0.633	0.434	0.105

Supplementary Figure S8 The percentage of CD3(-)CD20(+) mature B cells in 3 groups. The B cells are more stable in β-glucan intervention groups. * *P< 0.05*; error bar: 95% CI. Yeast derived β-glucan supplementation was initiated on day 7 of cycle 2 of systemic chemotherapy. * *P< 0.05.*
