# Supplementary material for: Beta-Glucan Supplementation Improves Clinical Outcomes and Preserves Immune Homeostasis in Patients with Advanced Solid Tumors Receiving Chemotherapy: A Prospective Phase II Randomized Three-Arm Clinical Trial
Source: Int J Mol Sci. 2026 Aug 11;27(16):7189. doi: 10.3390/ijms27167189 (PMC13513407; doi:10.3390/ijms27167189)
Supplement: Supplementary file 1 [file ijms-27-07189-s001.zip › Supplementary Figure S9 (CD28).pdf]

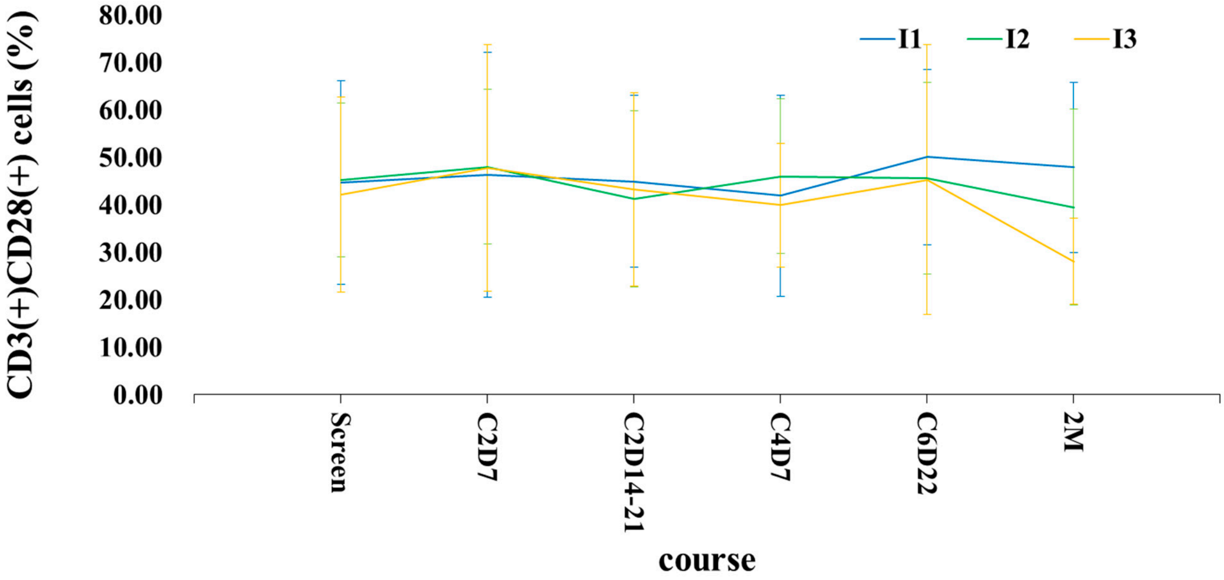

P<0.05			C2D7→Screen	C2D14-21→C2D7	C4D7→C2D14-21	C6D22→C4D7	2M→C6D22
CD3(+)CD28(+)(%)	Group 1 vs. Group 3 (powder *vs*. control)	B	-0.266	0.371	-0.479	1.588	-0.892
		P value	0.530	0.268	0.262	0.204	0.469
	Group 2 vs. Group 3 (capsule *vs* control)	B	-0.147	-0.095	0.250	0.797	-0.745
		P value	0.623	0.670	0.260	0.500	0.543

(A)

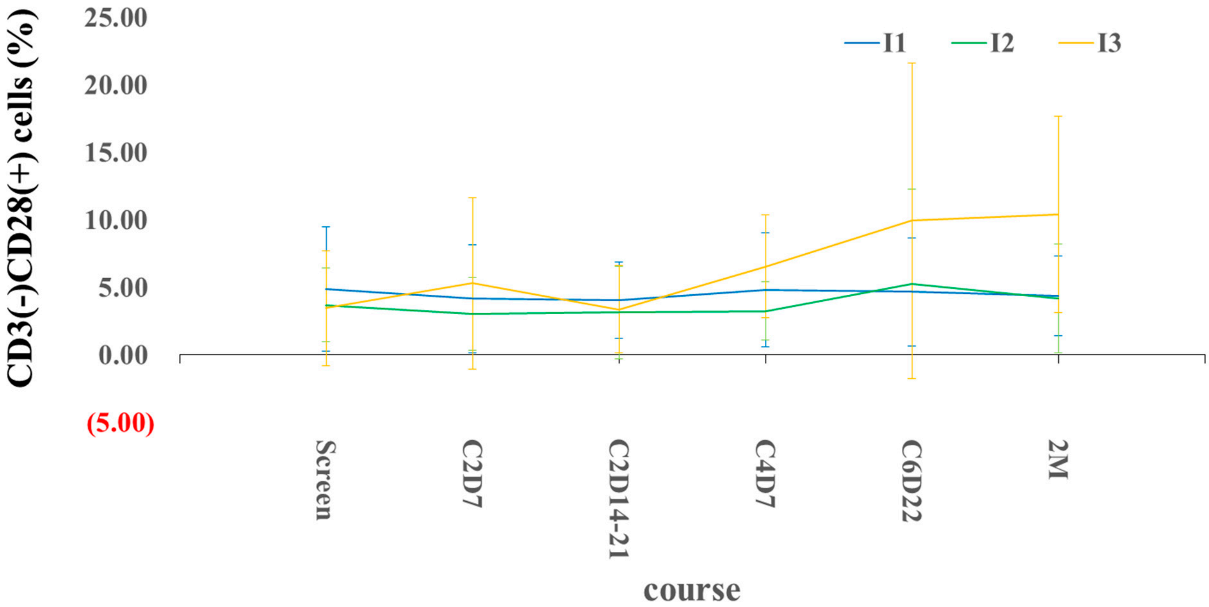

P<0.05			C2D7→Screen	C2D14-21→C2D7	C4D7→C2D14-21	C6D22→C4D7	2M→C6D22
CD3(-)CD28(+)(%)	Group 1 vs. Group 3 (powder *vs*. control)	B	-0.675	0.055	0.131	-0.933	-0.308
		P value	0.271	0.864	0.741	0.084	0.594
	Group 2 vs. Group 3 (capsule *vs* control)	B	-1.024	-0.107	0.230	0.342	-0.441
		P value	0.079	0.751	0.560	0.546	0.412

(B)

Supplementary Figure S9 The expression of (A) CD3(+)CD28(+) T cells and (B) CD3(-)CD28(+) NK cells

between 3 groups. There was a trend of increase of CD3(-)CD28(+) cells after chemotherapy in control group. The expressions were more stable in β-glucan intervention group. Yeast derived β-glucan supplementation was initiated on day 7 of cycle 2 of systemic chemotherapy.
