# Supplementary material for: Beta-Glucan Supplementation Improves Clinical Outcomes and Preserves Immune Homeostasis in Patients with Advanced Solid Tumors Receiving Chemotherapy: A Prospective Phase II Randomized Three-Arm Clinical Trial
Source: Int J Mol Sci. 2026 Aug 11;27(16):7189. doi: 10.3390/ijms27167189 (PMC13513407; doi:10.3390/ijms27167189)
Supplement: Supplementary file 1 [file ijms-27-07189-s001.zip › Supplementary Figure S10.pdf]

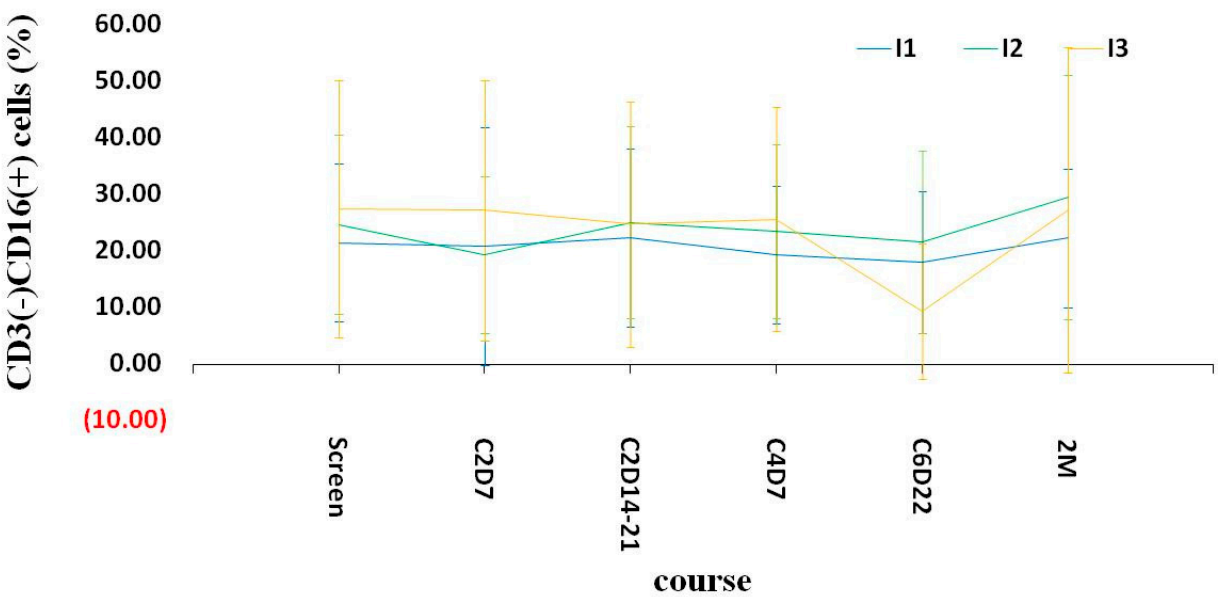

P<0.05			C2D7→Screen	C2D14-21→C2D7	C4D7→C2D14-21	C6D22→C4D7	2M→C6D22
CD3(-)CD16(+)(%)	Group 1 vs. Group 3 (powder *vs*. control)	B	-0.188	0.393	-0.321	1.191	-0.790
		P value	0.605	0.252	0.433	0.197	0.351
	Group 2 vs. Group 3 (capsule *vs* control)	B	-0.010	0.097	-0.081	1.108	-0.976
		P value	0.979	0.744	0.839	0.213	0.223

(A)

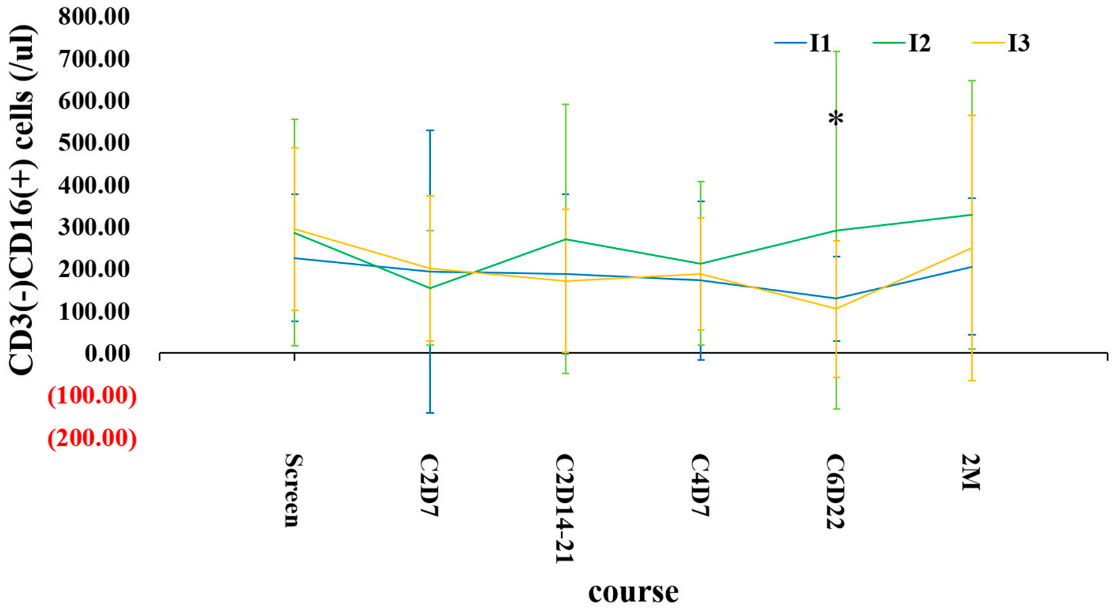

P<0.05			C2D7→Screen	C2D14-21→C2D7	C4D7→C2D14-21	C6D22→C4D7	2M→C6D22
CD3(-)CD16(+) cells	Group 1 vs. Group 3 (powder *vs*. control)	B	-0.433	0.429	-0.222	1.427	-0.552
		P value	0.511	0.356	0.664	0.091	0.420
	Group 2 vs. Group 3 (capsule *vs* control)	B	0.167	-0.331	0.442	1.805	-1.115
		P value	0.788	0.555	0.511	0.026*	0.077

(B)

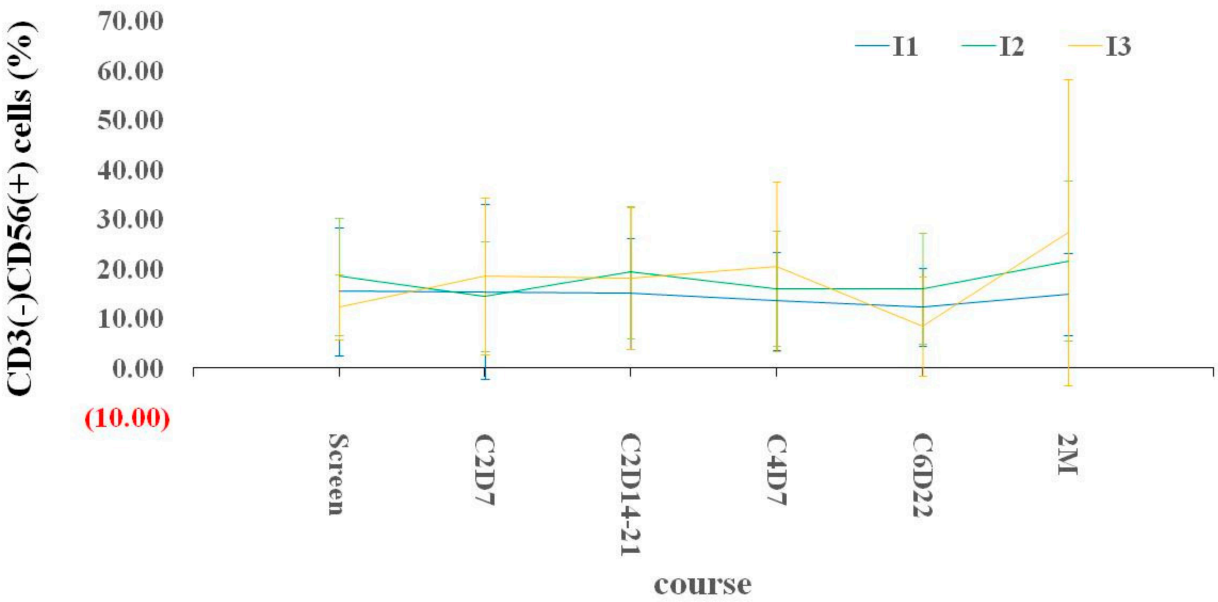

P<0.05			C2D7→Screen	C2D14-21→C2D7	C4D7→C2D14-21	C6D22→C4D7	2M→C6D22
CD3(-)CD56(+)(%)	Group 1 vs. Group 3 (powder *vs*. control)	B	-0.485	0.278	-0.080	1.148	-1.336
		P value	0.224	0.423	0.862	0.249	0.135
	Group 2 vs. Group 3 (capsule *vs* control)	B	-0.529	0.273	-0.050	1.471	-1.555
		P value	0.183	0.344	0.909	0.118	0.059

(C)

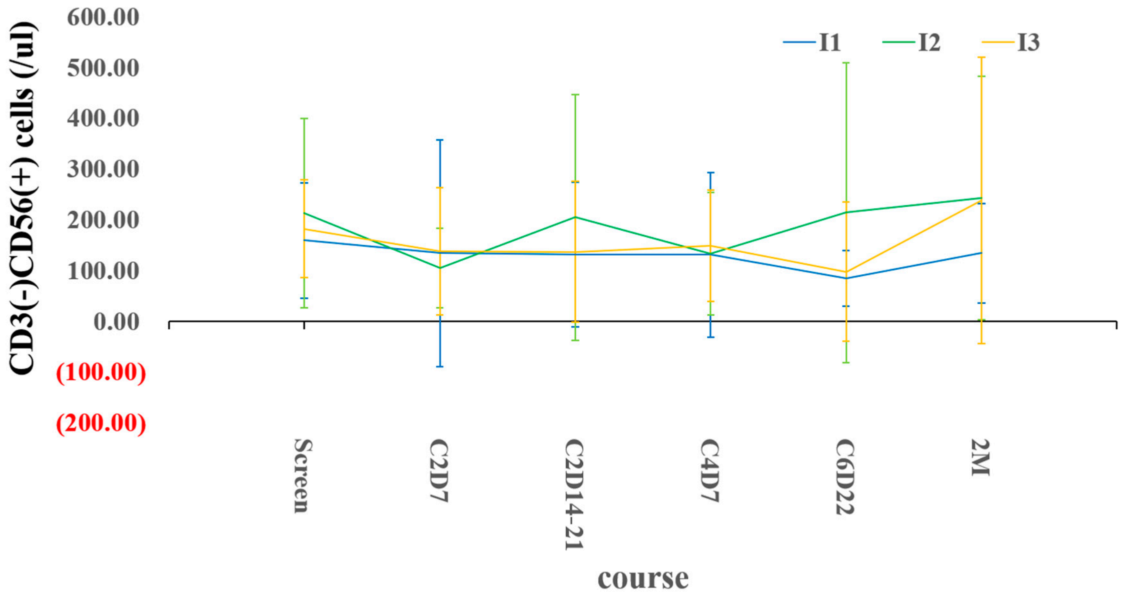

P<0.05			C2D7→Screen	C2D14-21→C2D7	C4D7→C2D14-21	C6D22→C4D7	2M→C6D22
CD3(-)CD56(+) cells	Group 1 vs. Group 3 (powder *vs*. control)	B	-0.227	0.325	-0.488	1.734	-1.153
		P value	0.721	0.447	0.389	0.076	0.121
	Group 2 vs. Group 3 (capsule *vs* control)	B	0.007	0.009	0.218	1.811	-1.239
		P value	0.991	0.983	0.700	0.052	0.074

(D)

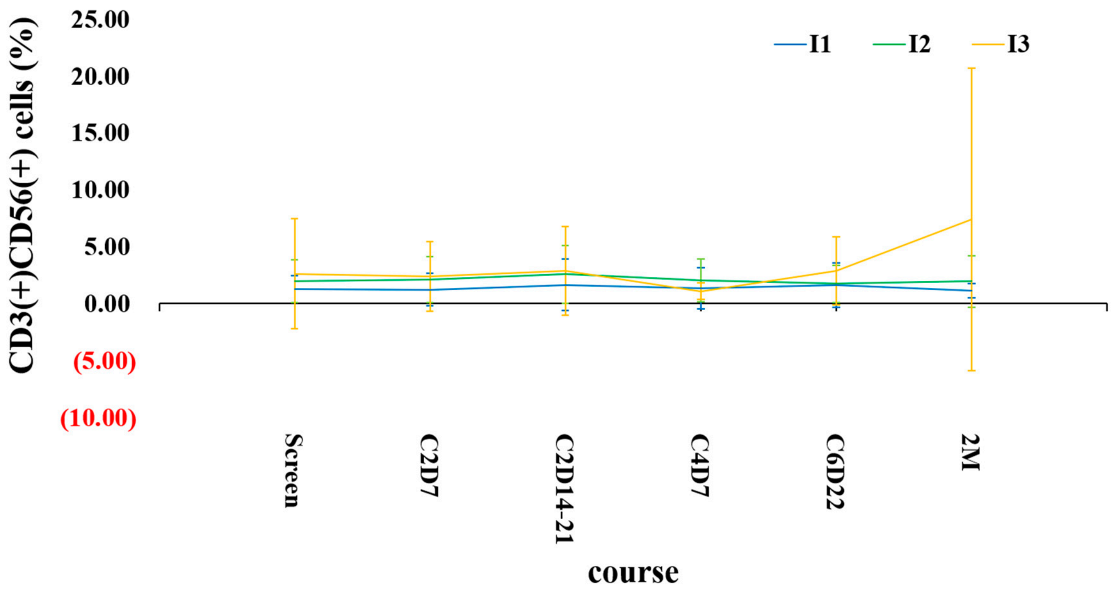

P<0.05			C2D7→Screen	C2D14-21→C2D7	C4D7→C2D14-21	C6D22→C4D7	2M→C6D22
CD3(+)CD56(+)(%)	Group 1 vs. Group 3 (powder *vs*. control)	B	-0.762	0.488	-0.443	0.592	-0.123
		P value	0.077	0.095	0.315	0.440	0.877
	Group 2 vs. Group 3 (capsule *vs* control)	B	-0.432	0.311	-0.081	0.382	-0.252
		P value	0.270	0.267	0.801	0.587	0.746

(E)

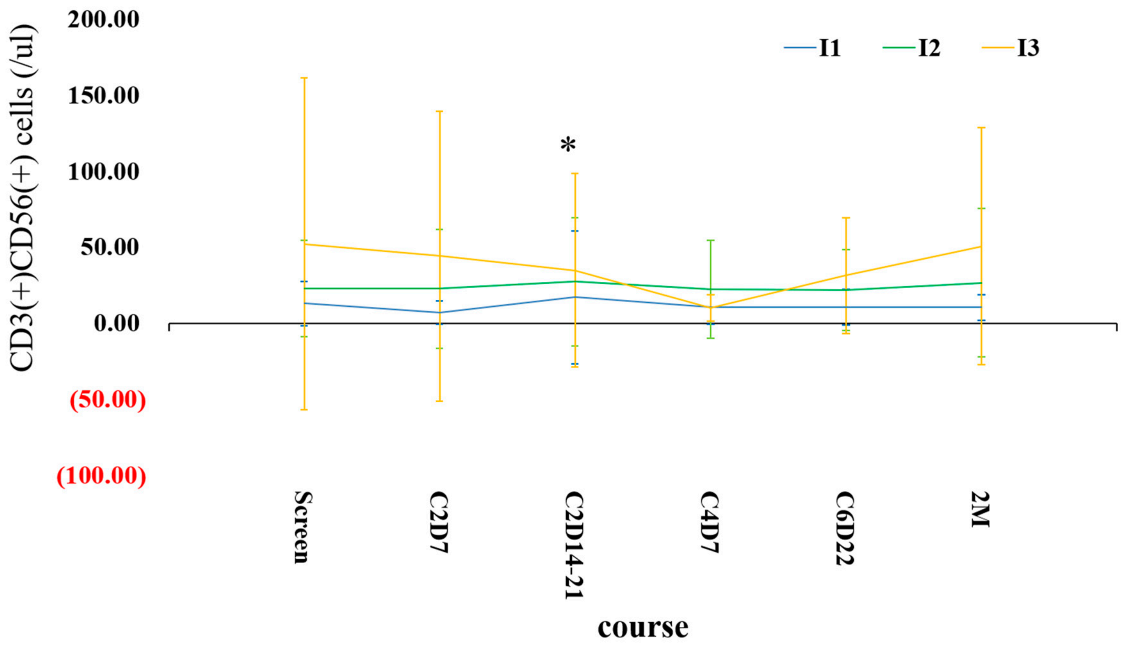

P<0.05			C2D7→Screen	C2D14-21→C2D7	C4D7→C2D14-21	C6D22→C4D7	2M→C6D22
CD3(+)CD56(+)cells	Group 1 vs. Group 3 (powder *vs*. control)	B	-0.272	0.887	-1.133	0.185	0.302
		P value	0.626	0.044*	0.068	0.758	0.675
	Group 2 vs. Group 3 (capsule *vs* control)	B	0.163	0.331	-0.039	0.300	0.384
		P value	0.756	0.446	0.943	0.605	0.591

(F)

Supplementary Figure S10 NK cells expressions in 3 groups. (A)(B) the percentage and total amount of CD16 (+) NK cells (C)(D) the percentage and total amount of CD3(-)CD56 (+) NK cells (E)(F) the percentage and total amount of CD3(+)CD56(+) NK-T cells. Yeast derived β-glucan supplementation was initiated on day 7 of cycle 2 of systemic chemotherapy. * $P < 0.05$.
