# Supplementary material for: Beta-Glucan Supplementation Improves Clinical Outcomes and Preserves Immune Homeostasis in Patients with Advanced Solid Tumors Receiving Chemotherapy: A Prospective Phase II Randomized Three-Arm Clinical Trial
Source: Int J Mol Sci. 2026 Aug 11;27(16):7189. doi: 10.3390/ijms27167189 (PMC13513407; doi:10.3390/ijms27167189)
Supplement: Supplementary file 1 [file ijms-27-07189-s001.zip › Supplementary Figures S2-7.pdf]

A

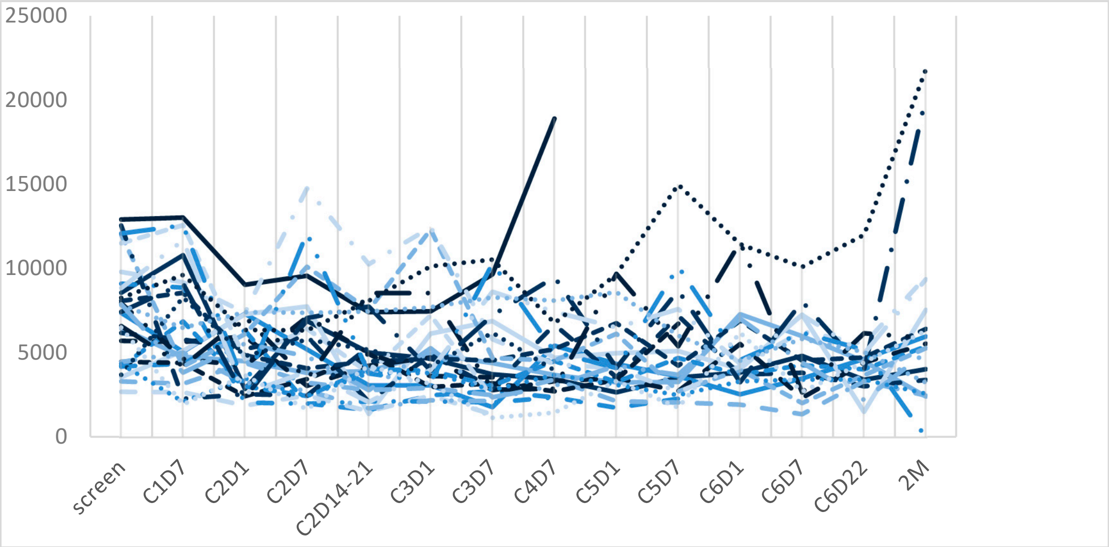
25000
20000
15000
10000
5000
0
screen
C1D7
C2D1
C2D7
C2D14-21
C3D1
C3D7
C4D7
C5D1
C5D7
C6D1
C6D7
C6D22
2M

B

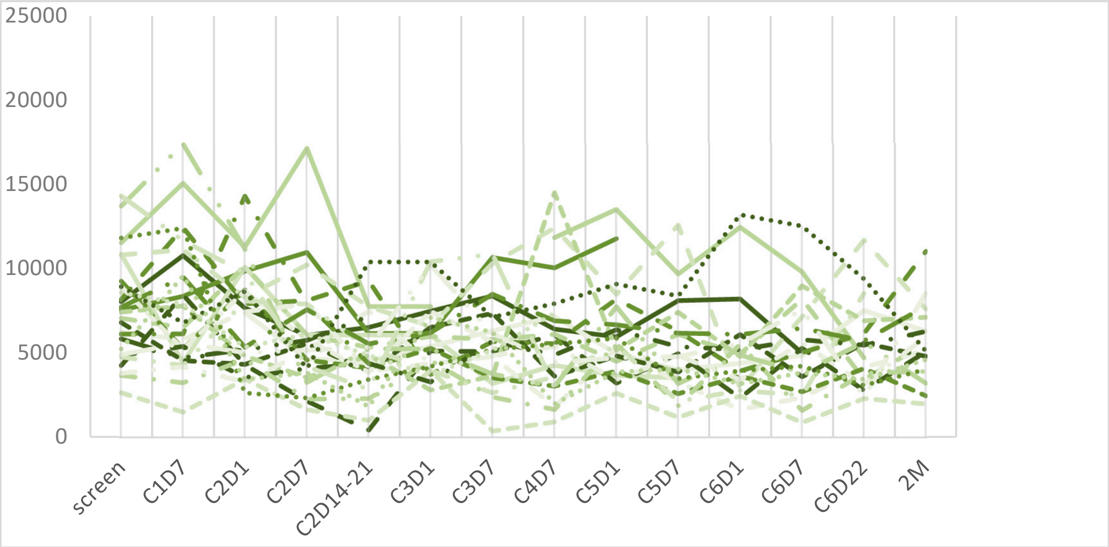
25000
20000
15000
10000
5000
0
screen
C1D7
C2D1
C2D7
C2D14-21
C3D1
C3D7
C4D7
C5D1
C5D7
C6D1
C6D7
C6D22
2M

C

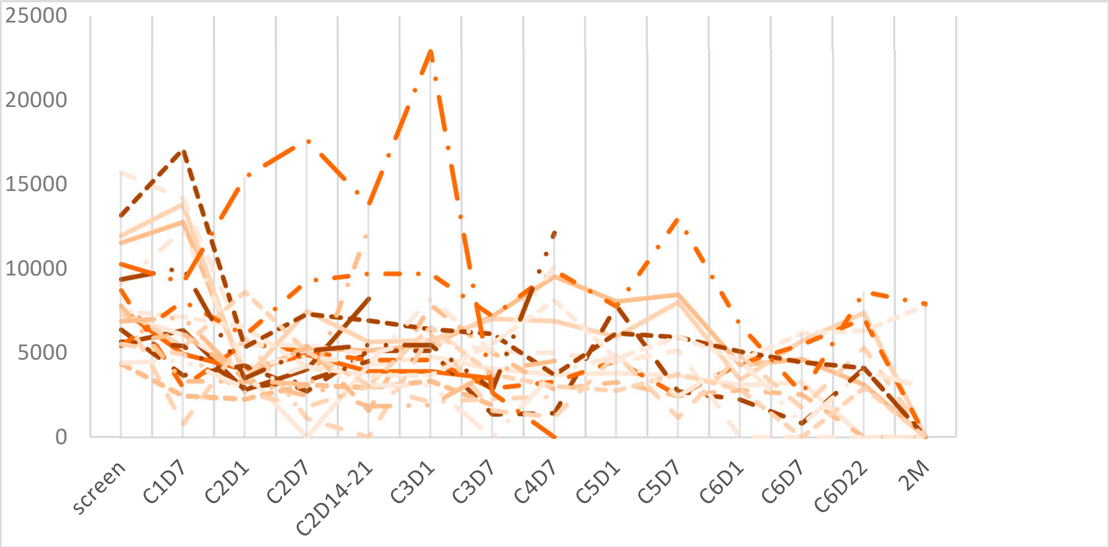
25000
20000
15000
10000
5000
0
screen
C1D7
C2D1
C2D7
C2D14-21
C3D1
C3D7
C4D7
C5D1
C5D7
C6D1
C6D7
C6D22
2M

D

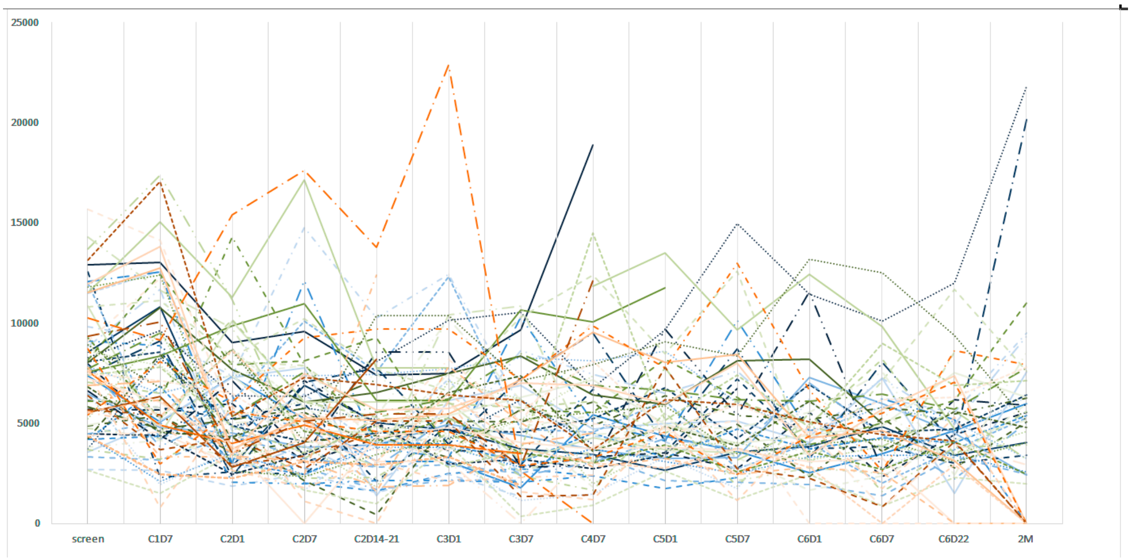

Supplementary Figure S2. The detail of patients' white blood cells. We only showed the detail data of patients who was still in the trial at cycle2day14-21. (A) patients in powder group, n=30. (B) patients in capsule group, n=29; (C) patients in control group, n=23; (D) merge 3 groups. Yeast derived β-glucan supplementation was initiated on day 7 of cycle 2 of systemic chemotherapy.

A

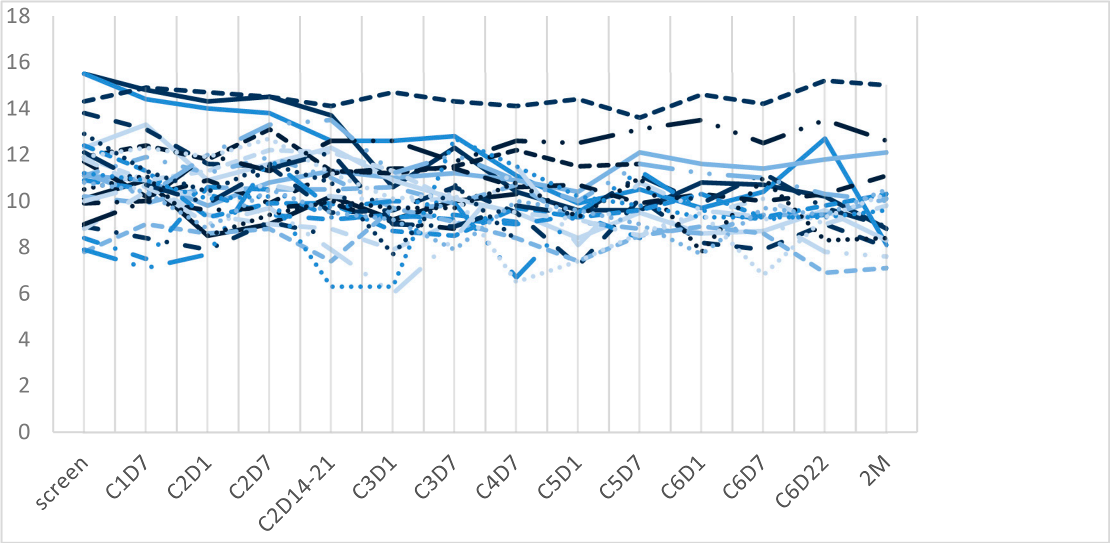
18
16
14
12
10
8
6
4
2
0
screen
C1D7
C2D1
C2D7
C2D14-21
C3D1
C3D7
C4D7
C5D1
C5D7
C6D1
C6D7
C6D22
2M

B

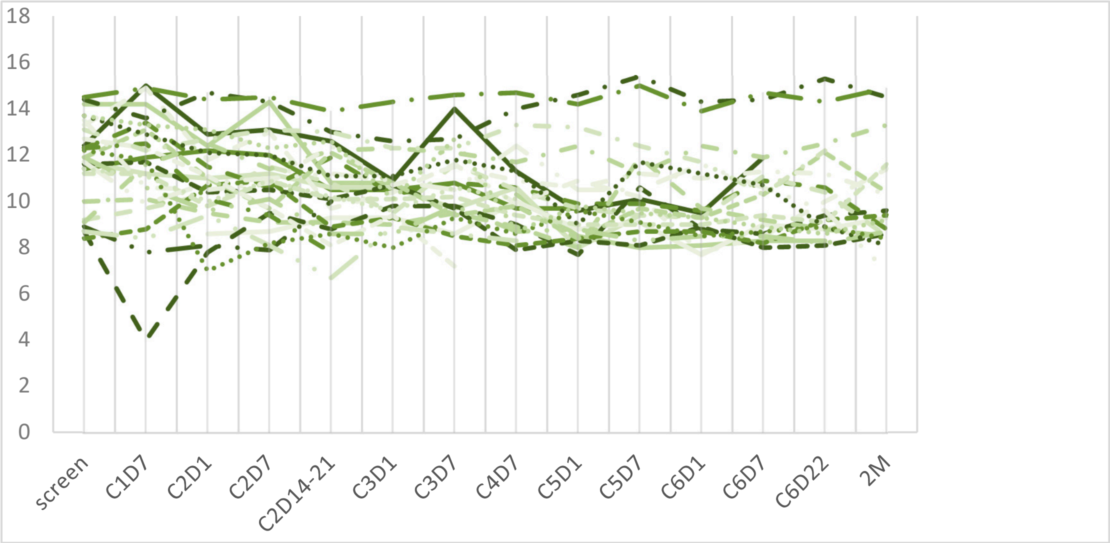
18
16
14
12
10
8
6
4
2
0
screen
C1D7
C2D1
C2D7
C2D14-21
C3D1
C3D7
C4D7
C5D1
C5D7
C6D1
C6D7
C6D22
2M

C

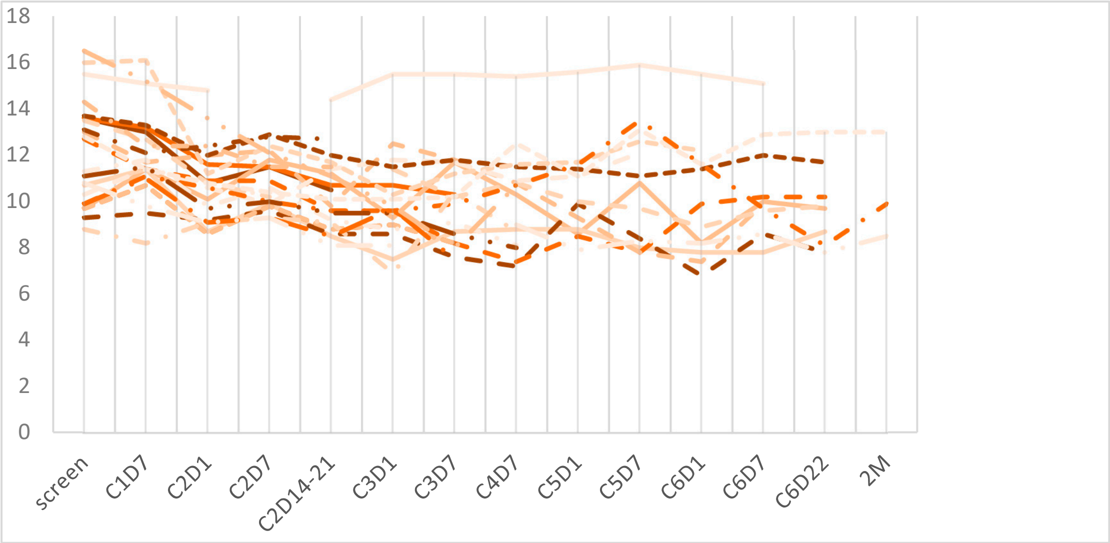
18
16
14
12
10
8
6
4
2
0
screen
C1D7
C2D1
C2D7
C2D14-21
C3D1
C3D7
C4D7
C5D1
C5D7
C6D1
C6D7
C6D22
2M

D

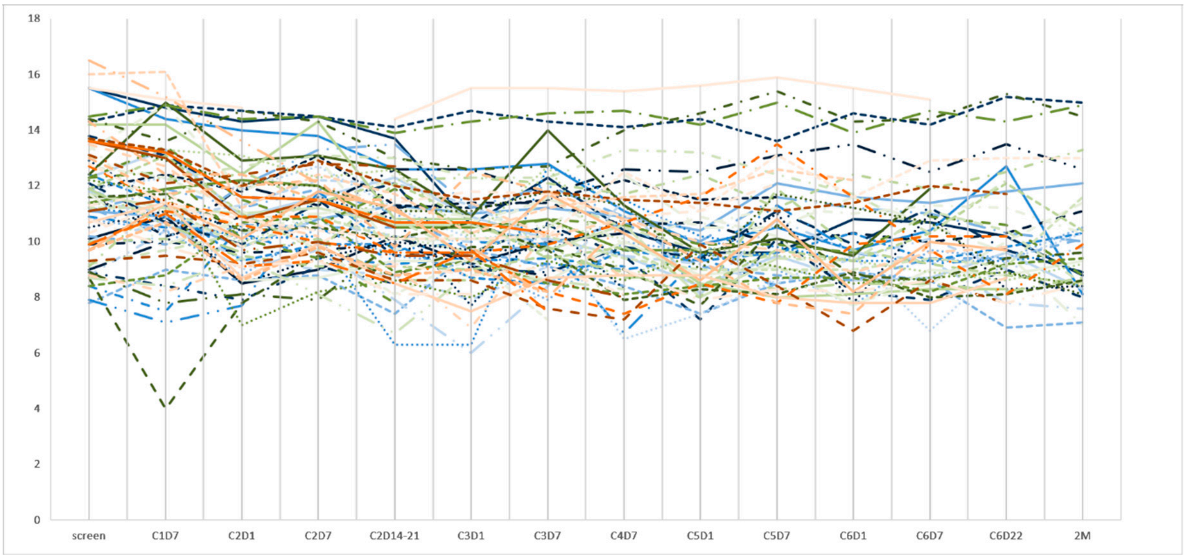

Supplementary Figure S3. The detail of patients' hemoglobin. We only showed the detail data of patients who was still in the trial at cycle2day14-21. (A) patients in powder group, n=30. (B) patients in capsule group, n=29; (C) patients in control group, n=23; (D) merge 3 groups. Yeast derived β-glucan supplementation was initiated on day 7 of cycle 2 of systemic chemotherapy.

A

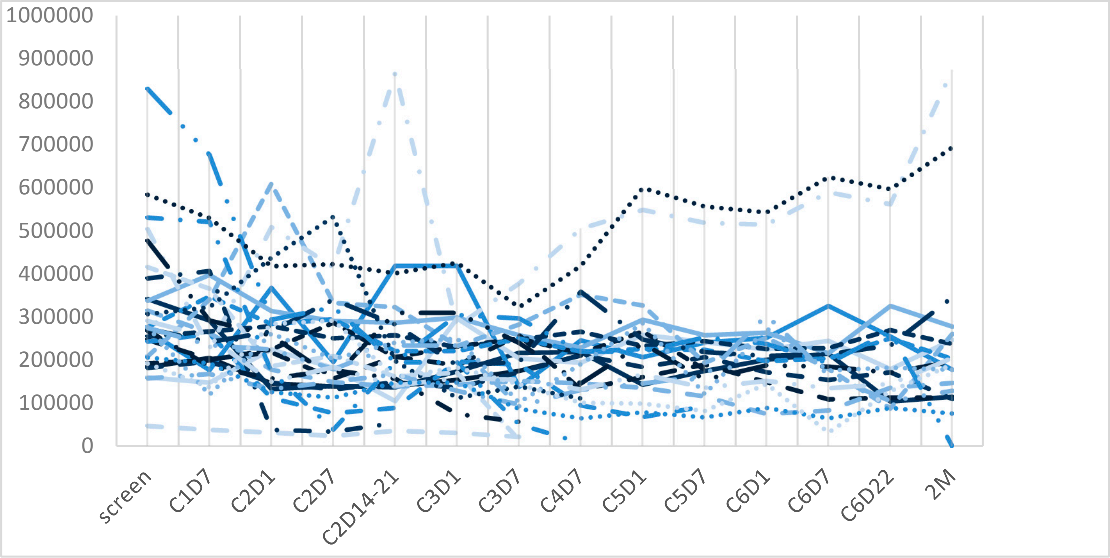
1000000
900000
800000
700000
600000
500000
400000
300000
200000
100000
0
screen
C1D7
C2D1
C2D7
C2D14-21
C3D1
C3D7
C4D7
C5D1
C5D7
C6D1
C6D7
C6D22
2M

B

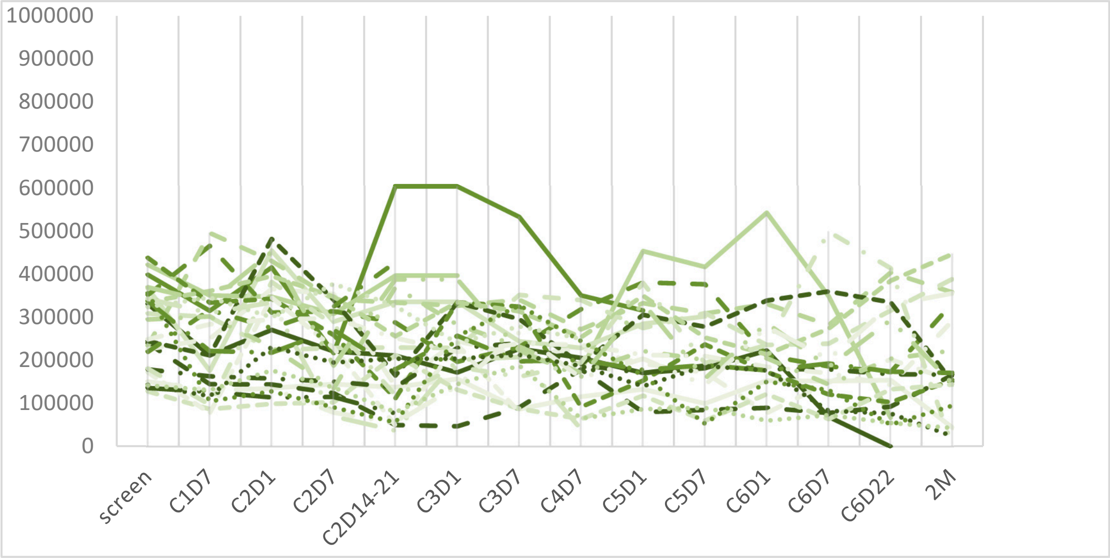
1000000
900000
800000
700000
600000
500000
400000
300000
200000
100000
0
screen
C1D7
C2D1
C2D7
C2D14-21
C3D1
C3D7
C4D7
C5D1
C5D7
C6D1
C6D7
C6D22
2M

C

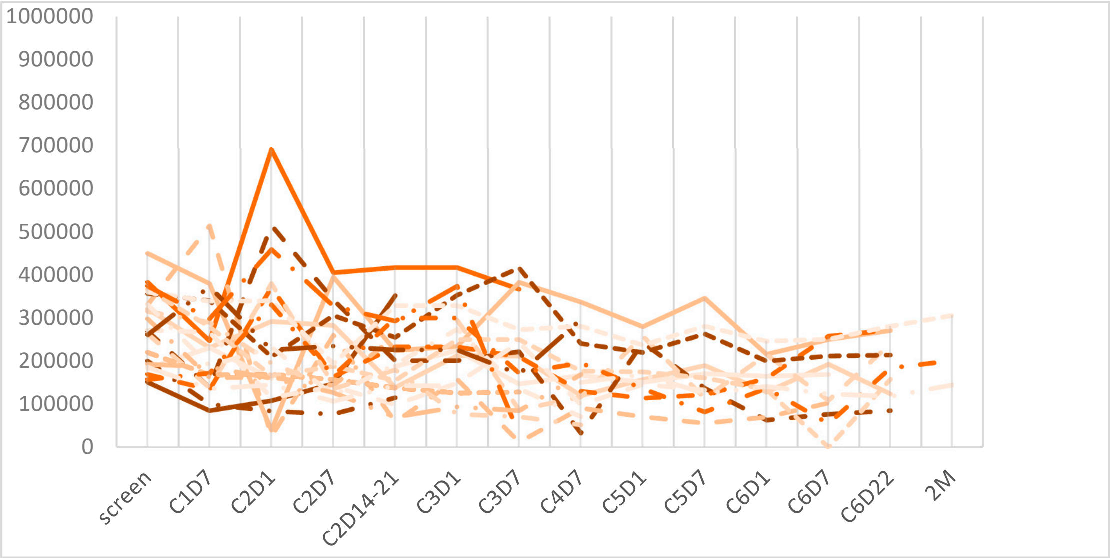

D

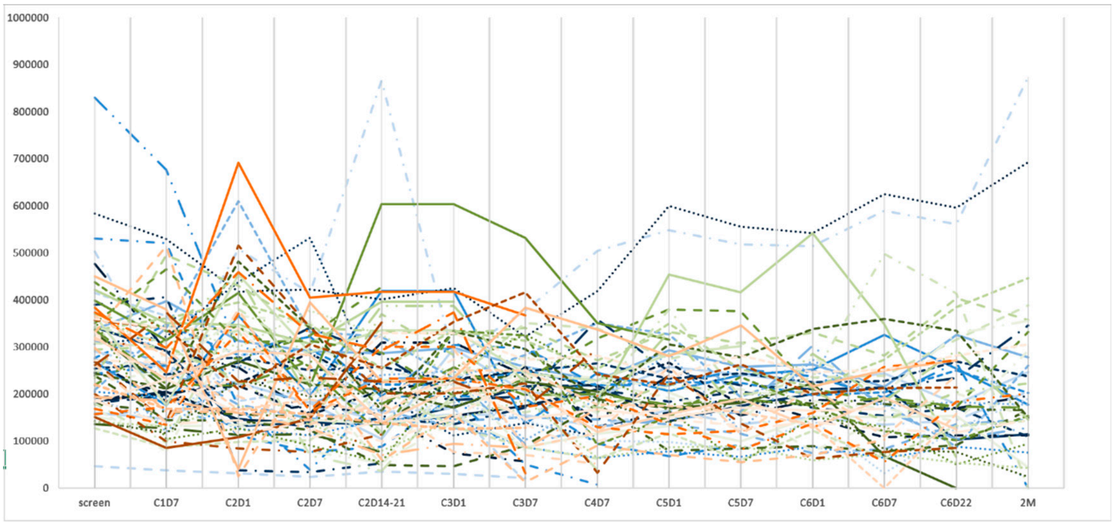

Supplementary Figure S4. The detail of patients' platelet. We only showed the detail data of patients who was still in the trial at cycle2day14-21. (A) patients in powder group, n=30. (B) patients in capsule group, n=29; (C) patients in control group, n=23; (D) merge 3 groups. Yeast derived β-glucan supplementation was initiated on day 7 of cycle 2 of systemic chemotherapy.

A

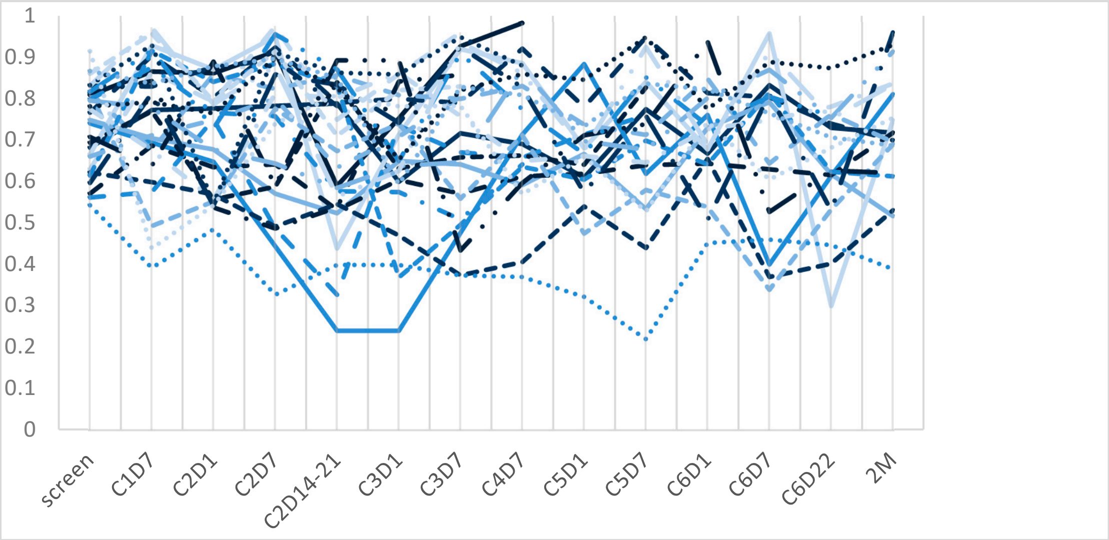
1
0.9
0.8
0.7
0.6
0.5
0.4
0.3
0.2
0.1
0
screen
C1D7
C2D1
C2D7
C2D14-21
C3D1
C3D7
C4D7
C5D1
C5D7
C6D1
C6D7
C6D22
2M

B

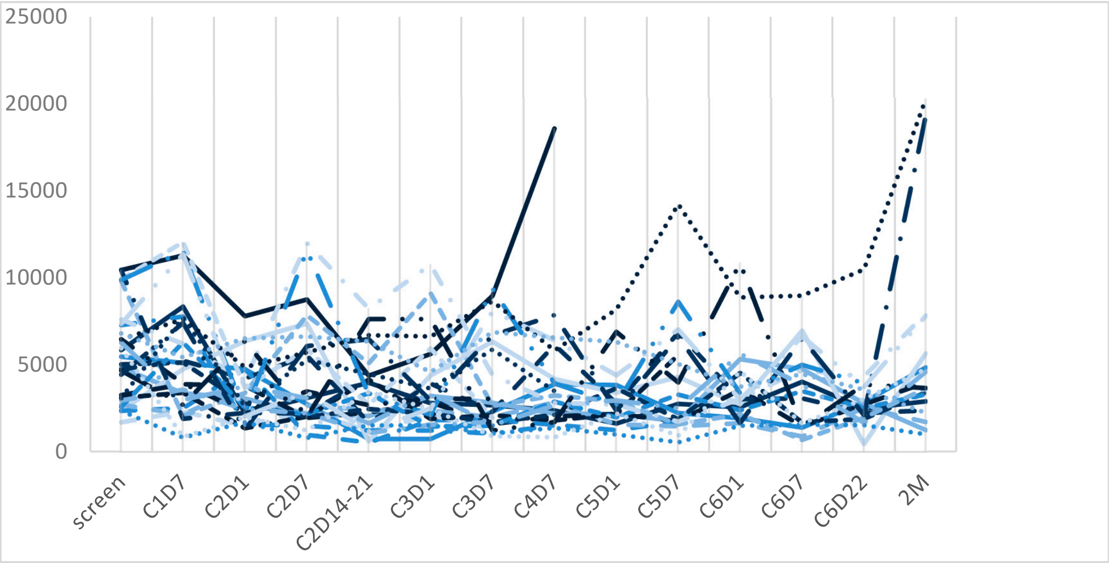
25000
20000
15000
10000
5000
0
screen
C1D7
C2D1
C2D7
C2D14-21
C3D1
C3D7
C4D7
C5D1
C5D7
C6D1
C6D7
C6D22
2M

C

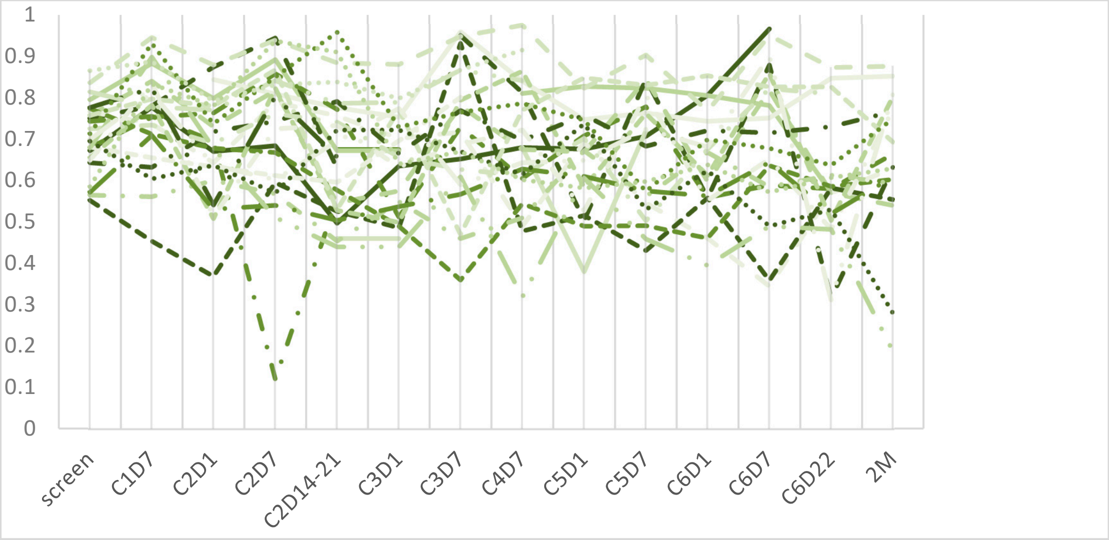
1
0.9
0.8
0.7
0.6
0.5
0.4
0.3
0.2
0.1
0
screen
C1D7
C2D1
C2D7
C2D14-21
C3D1
C3D7
C4D7
C5D1
C5D7
C6D1
C6D7
C6D22
2M

D

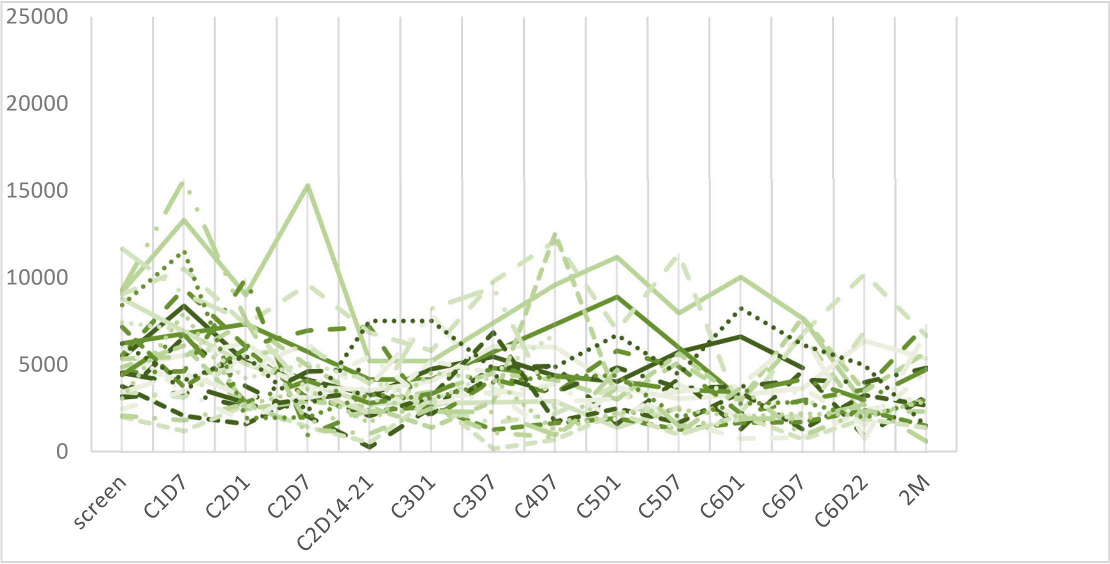
25000
20000
15000
10000
5000
0
screen
C1D7
C2D1
C2D7
C2D14-21
C3D1
C3D7
C4D7
C5D1
C5D7
C6D1
C6D7
C6D22
2M

E

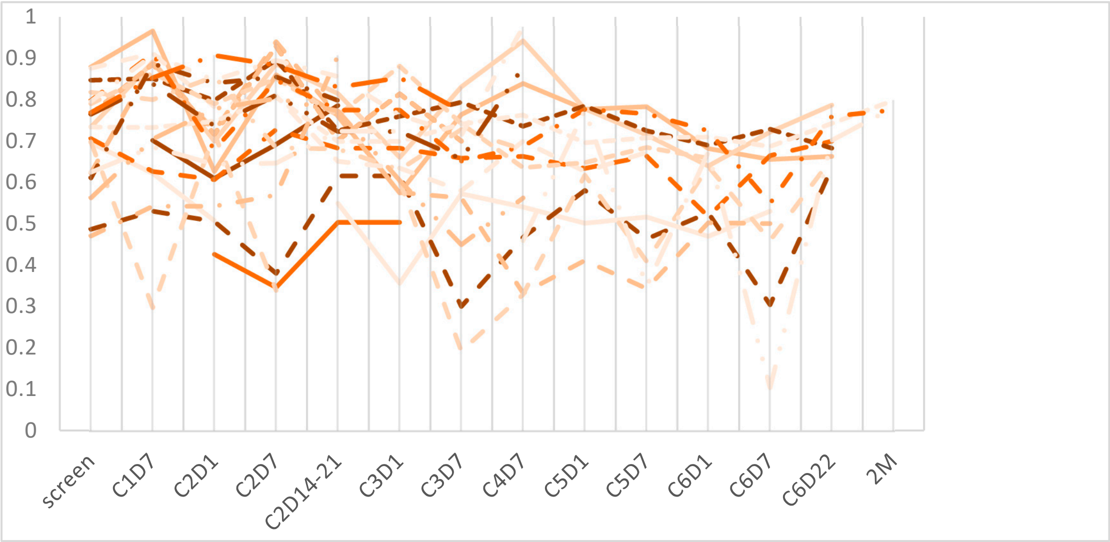
1
0.9
0.8
0.7
0.6
0.5
0.4
0.3
0.2
0.1
0
screen
C1D7
C2D1
C2D7
C2D14-21
C3D1
C3D7
C4D7
C5D1
C5D7
C6D1
C6D7
C6D22
2M

F

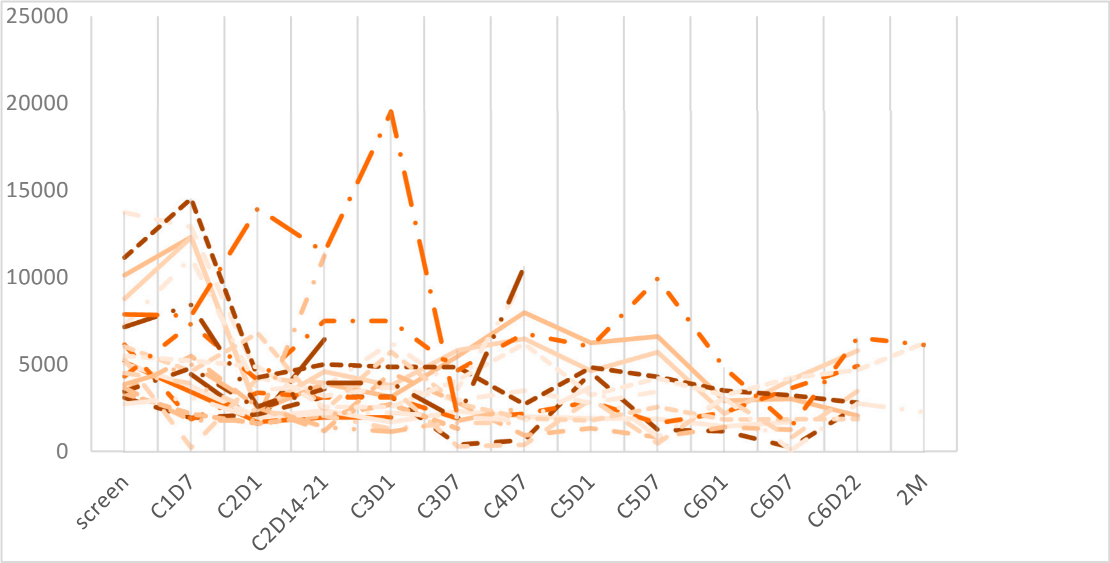

G

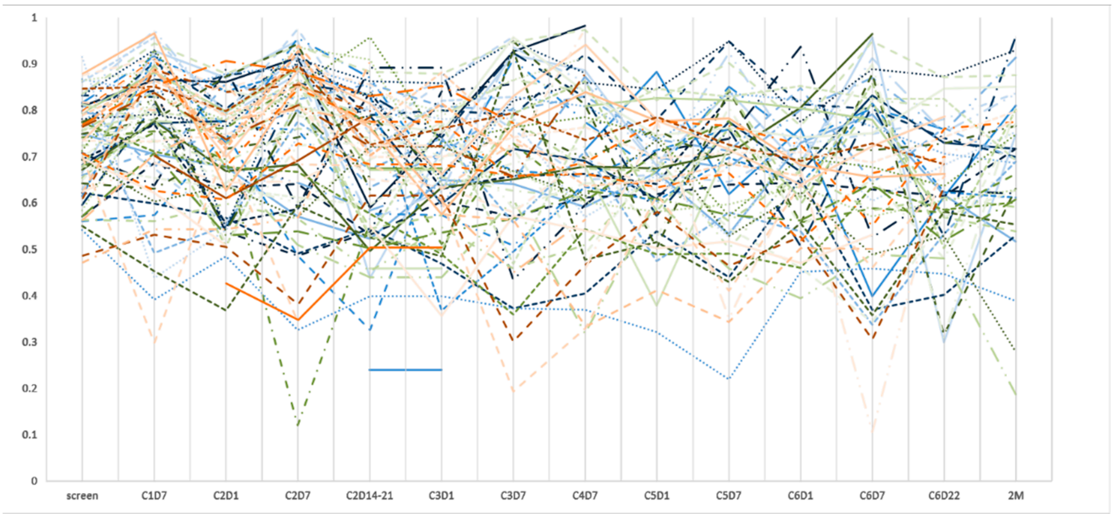

H

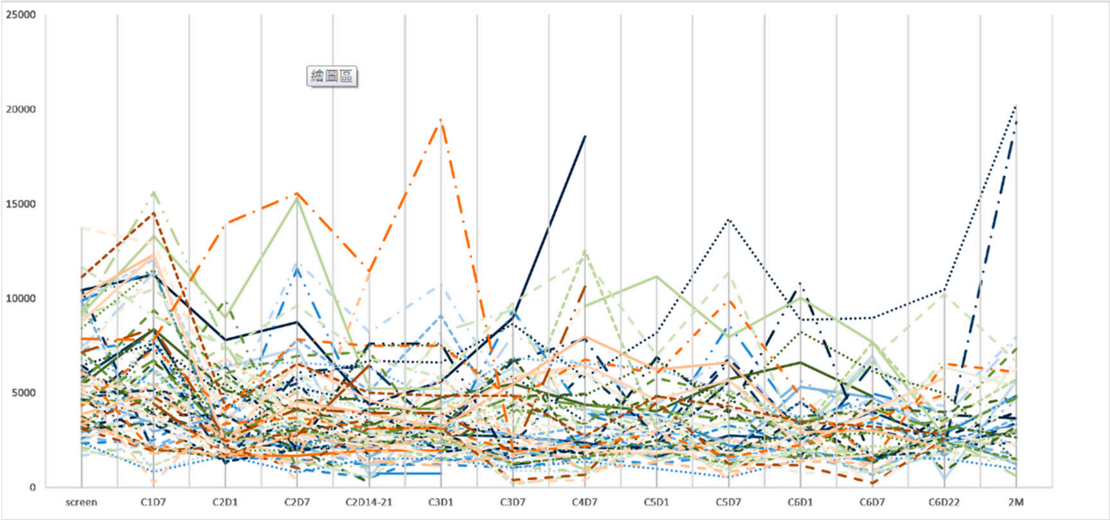

Supplementary Figure S5. The detail of patients' neutrophil percentage (A,C,E,G) and neutrophil count

(B,D,F,H). We only showed the detail data of patients who was still in the trial at cycle2day14-21. Blue color line: patients in powder group, n=30. Green color line: patients in capsule group, n=29; Orange color line: patients in control group, n=23; Yeast derived β-glucan supplementation was initiated on day 7 of cycle 2 of systemic chemotherapy.

A

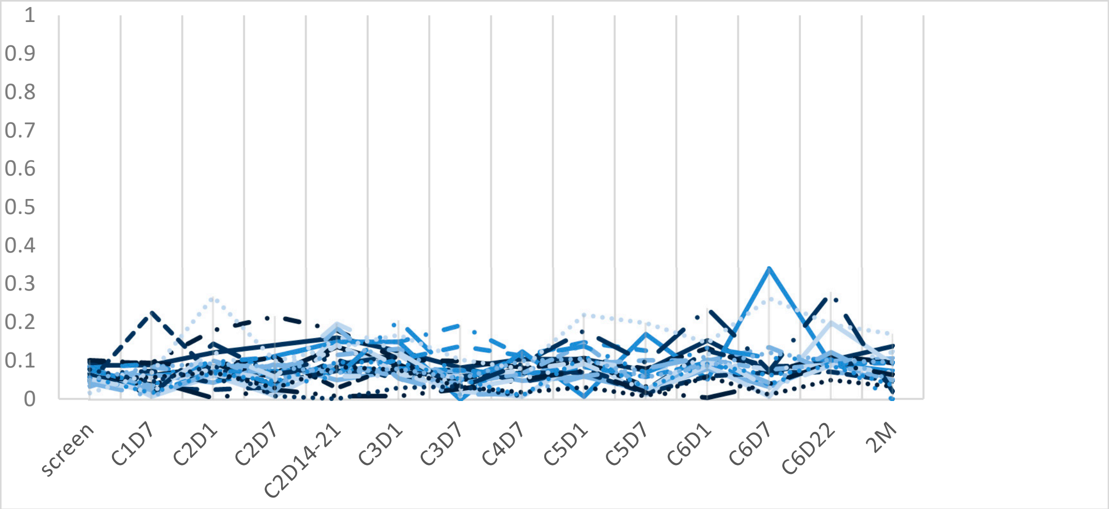
1
0.9
0.8
0.7
0.6
0.5
0.4
0.3
0.2
0.1
0
screen
C1D7
C2D1
C2D7
C2D14-21
C3D1
C3D7
C4D7
C5D1
C5D7
C6D1
C6D7
C6D22
2M

B

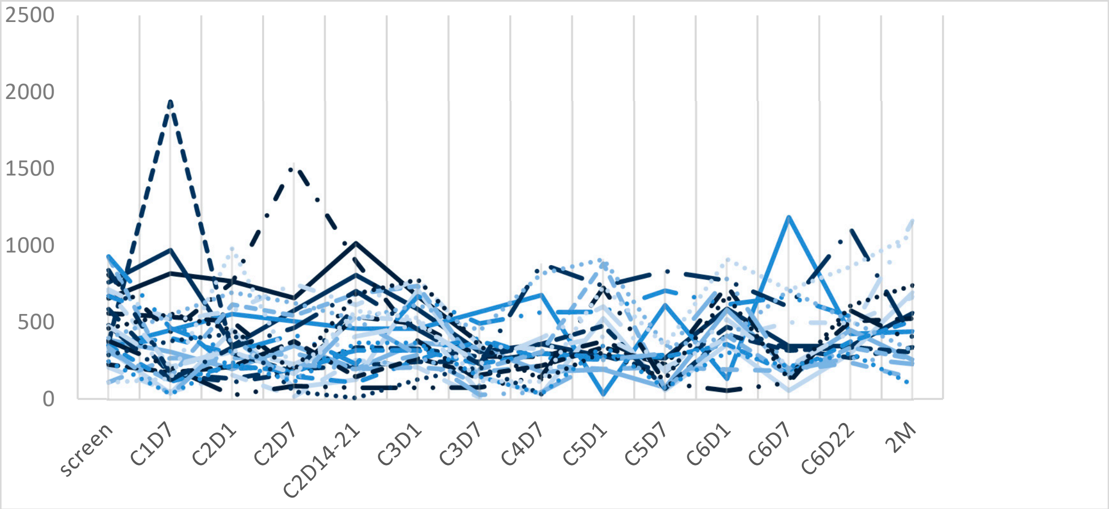
2500
2000
1500
1000
500
0
screen
C1D7
C2D1
C2D7
C2D14-21
C3D1
C3D7
C4D7
C5D1
C5D7
C6D1
C6D7
C6D22
2M

C

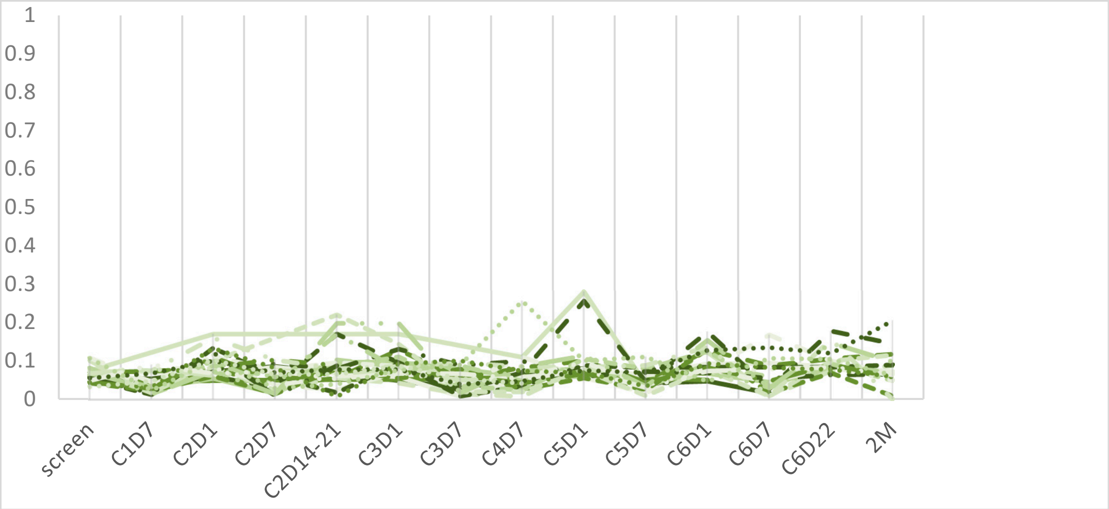
1
0.9
0.8
0.7
0.6
0.5
0.4
0.3
0.2
0.1
0
screen
C1D7
C2D1
C2D7
C2D14-21
C3D1
C3D7
C4D7
C5D1
C5D7
C6D1
C6D7
C6D22
2M

D

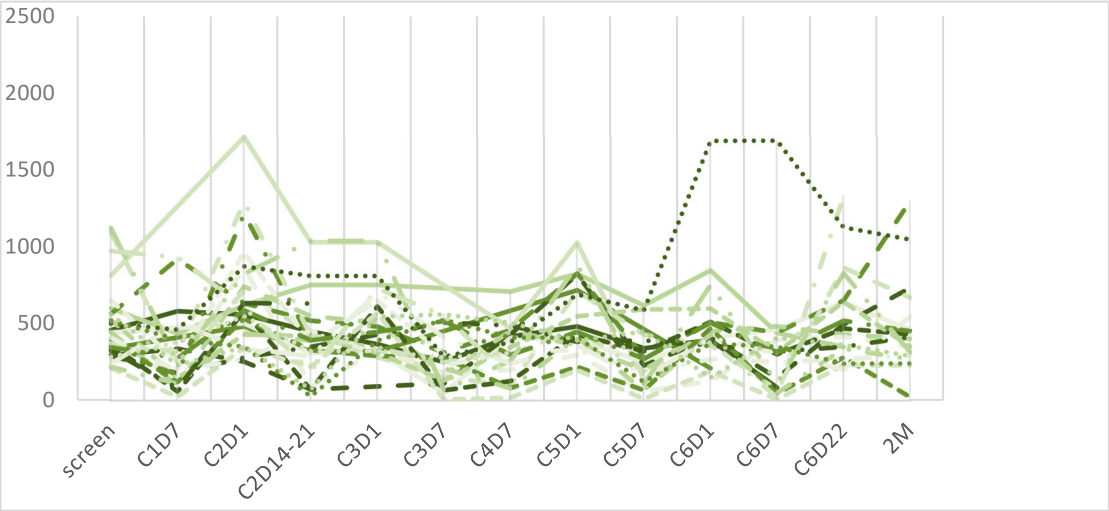
2500
2000
1500
1000
500
0
screen
C1D7
C2D1
C2D14-21
C3D1
C3D7
C4D7
C5D1
C5D7
C6D1
C6D7
C6D22
2M

E

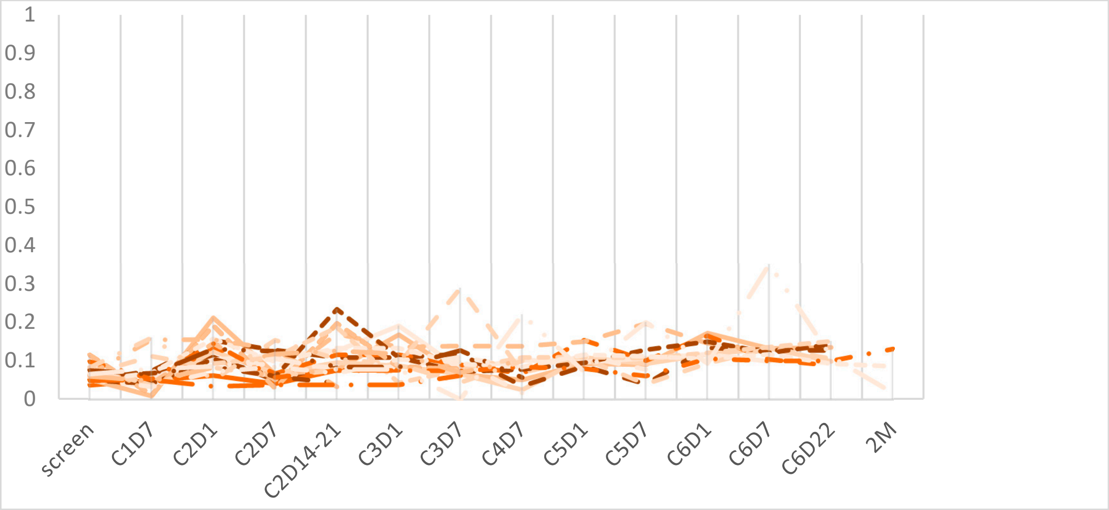
1
0.9
0.8
0.7
0.6
0.5
0.4
0.3
0.2
0.1
0
screen
C1D7
C2D1
C2D7
C2D14-21
C3D1
C3D7
C4D7
C5D1
C5D7
C6D1
C6D7
C6D22
2M

F

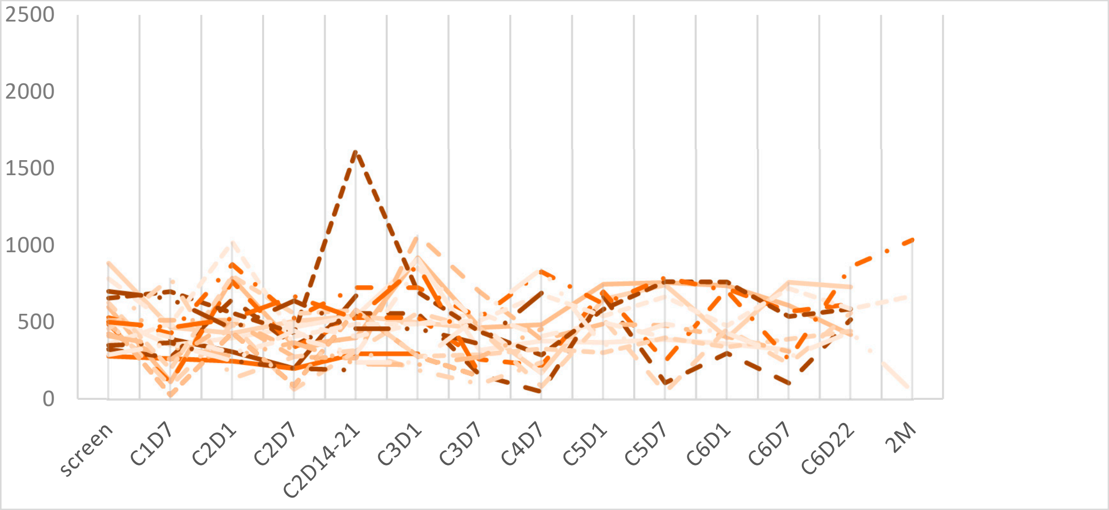
2500
2000
1500
1000
500
0
screen
C1D7
C2D1
C2D7
C2D14-21
C3D1
C3D7
C4D7
C5D1
C5D7
C6D1
C6D7
C6D22
2M

G

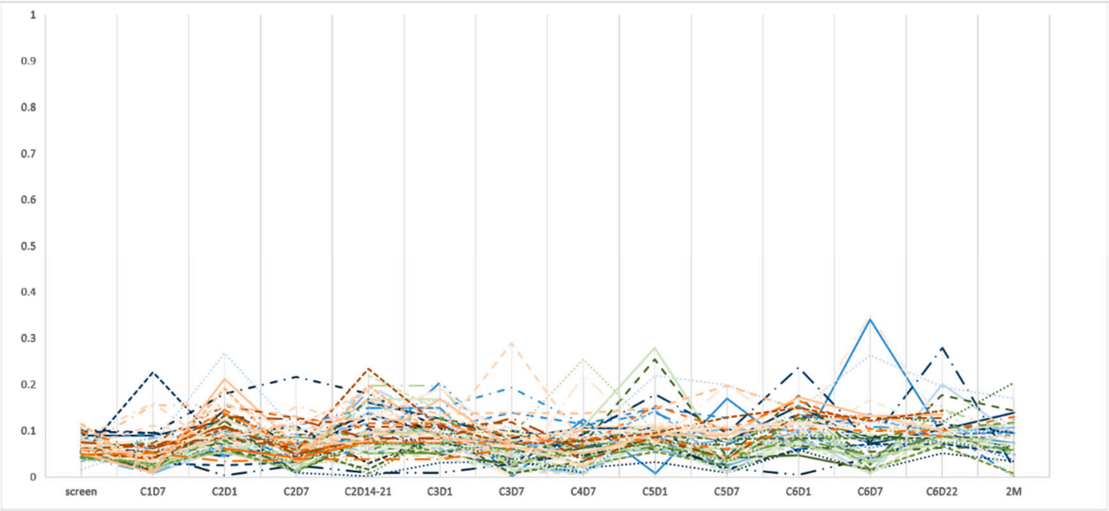

H

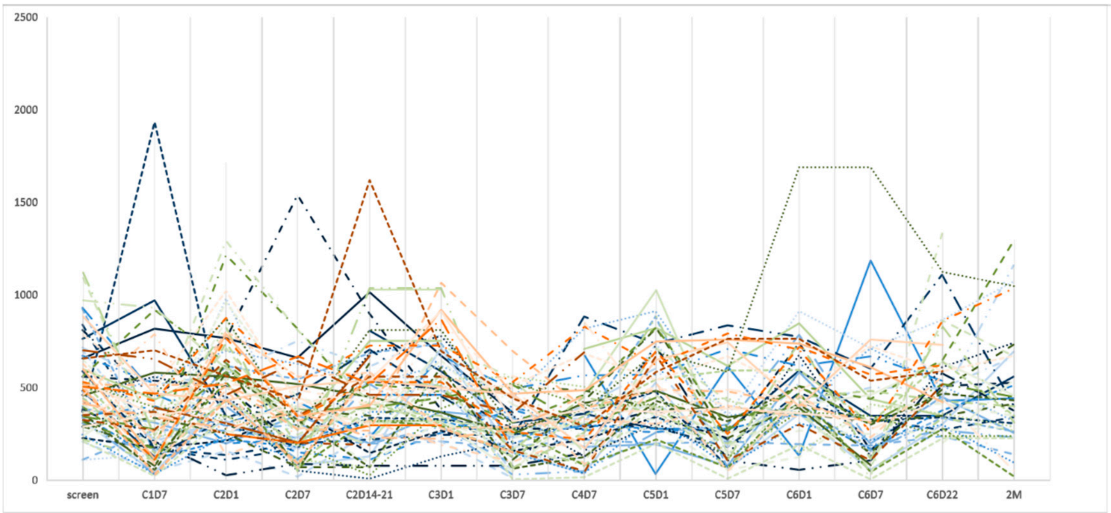

Supplementary Figure S6. The detail of patients' monocyte percentage (A,C,E,G) and monocyte count (B,D,F,H). We only showed the detail data of patients who was still in the trial at cycle2day14-21. Blue color line: patients in powder group, n=30. Green color line: patients in capsule group, n=29; Orange color line: patients in control group, n=23; (D) merge 3 groups. Yeast derived β-glucan supplementation was initiated on day 7 of cycle 2 of systemic chemotherapy.

A

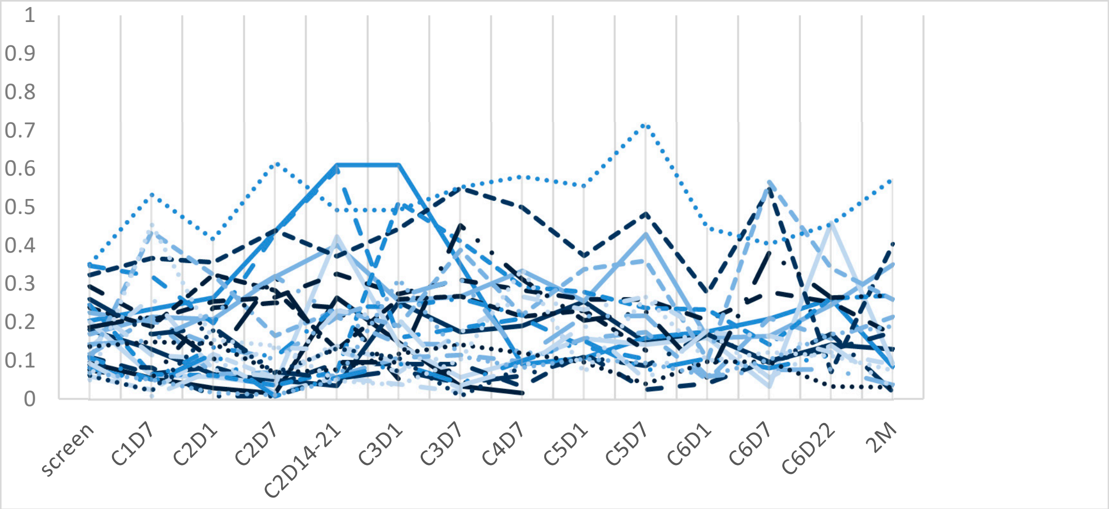
1
0.9
0.8
0.7
0.6
0.5
0.4
0.3
0.2
0.1
0
screen
C1D7
C2D1
C2D7
C2D14-21
C3D1
C3D7
C4D7
C5D1
C5D7
C6D1
C6D7
C6D22
2M

B

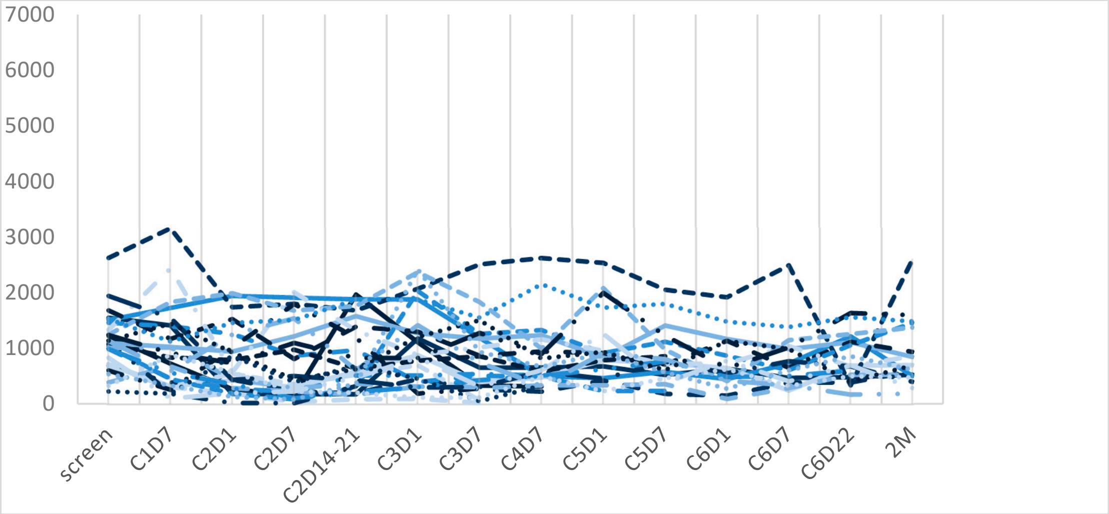
7000
6000
5000
4000
3000
2000
1000
0
screen
C1D7
C2D1
C2D7
C2D14-21
C3D1
C3D7
C4D7
C5D1
C5D7
C6D1
C6D7
C6D22
2M

C

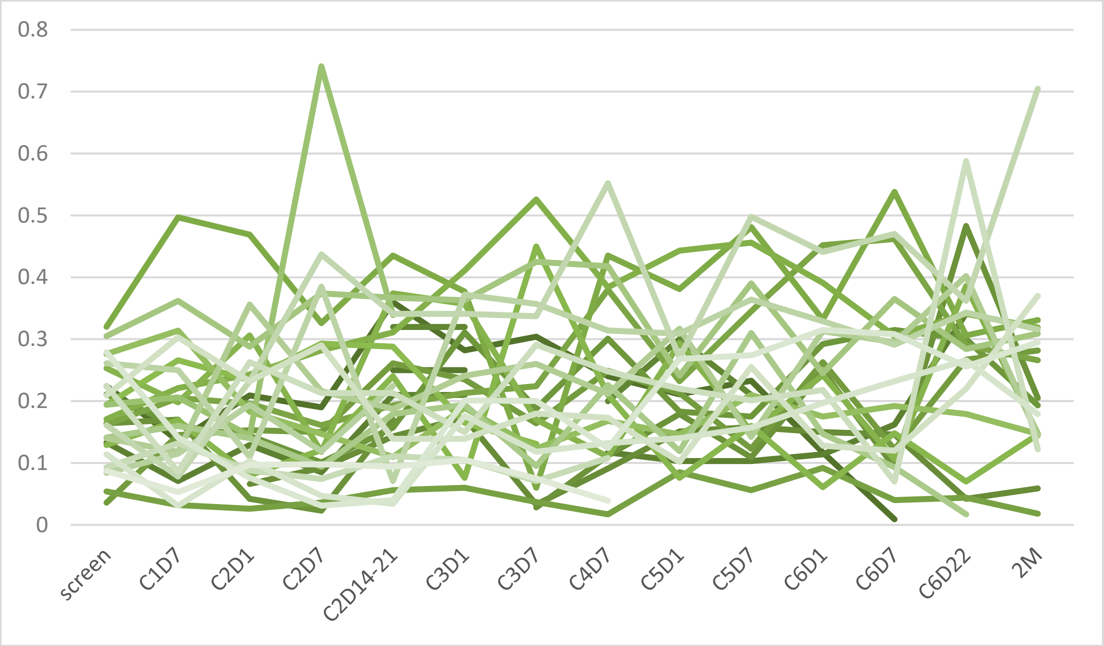
0.8
0.7
0.6
0.5
0.4
0.3
0.2
0.1
0
screen
C1D7
C2D1
C2D7
C2D14-21
C3D1
C3D7
C4D7
C5D1
C5D7
C6D1
C6D7
C6D22
2M

D

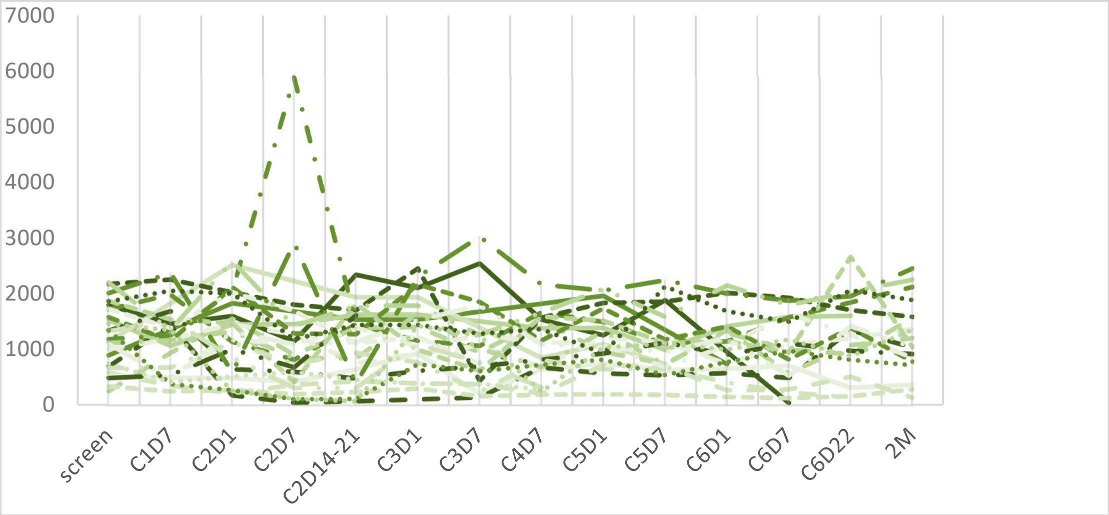
7000
6000
5000
4000
3000
2000
1000
0
screen
C1D7
C2D1
C2D7
C2D14-21
C3D1
C3D7
C4D7
C5D1
C5D7
C6D1
C6D7
C6D22
2M

E

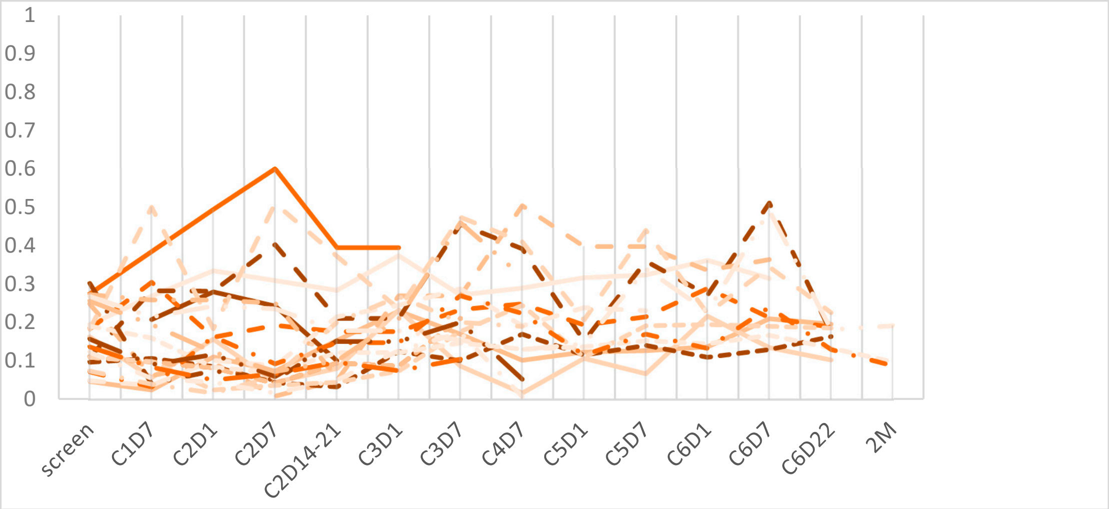
1
0.9
0.8
0.7
0.6
0.5
0.4
0.3
0.2
0.1
0
screen
C1D7
C2D1
C2D7
C2D14-21
C3D1
C3D7
C4D7
C5D1
C5D7
C6D1
C6D7
C6D22
2M

F

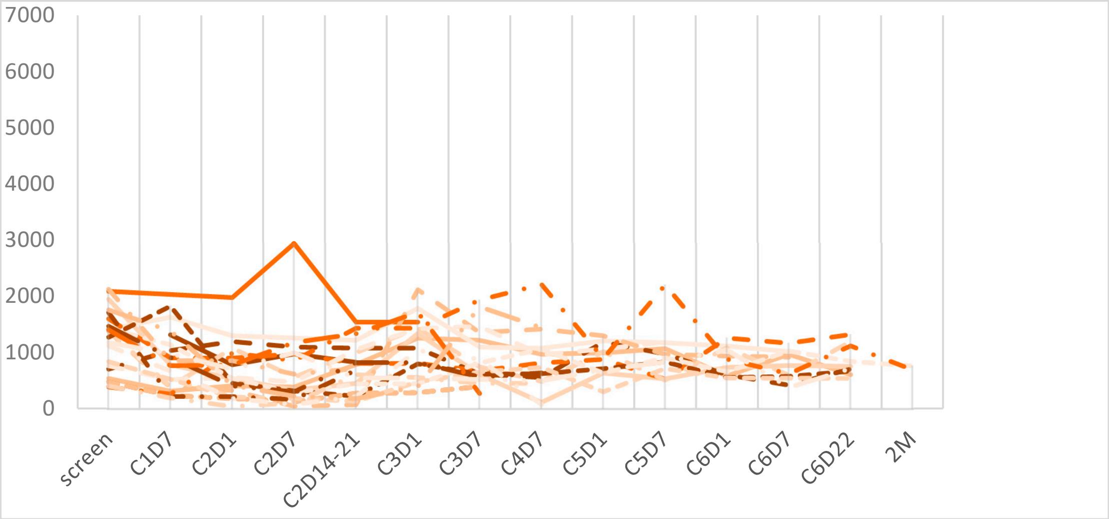
7000
6000
5000
4000
3000
2000
1000
0
screen
C1D7
C2D1
C2D7
C2D14-21
C3D1
C3D7
C4D7
C5D1
C5D7
C6D1
C6D7
C6D22
2M

G

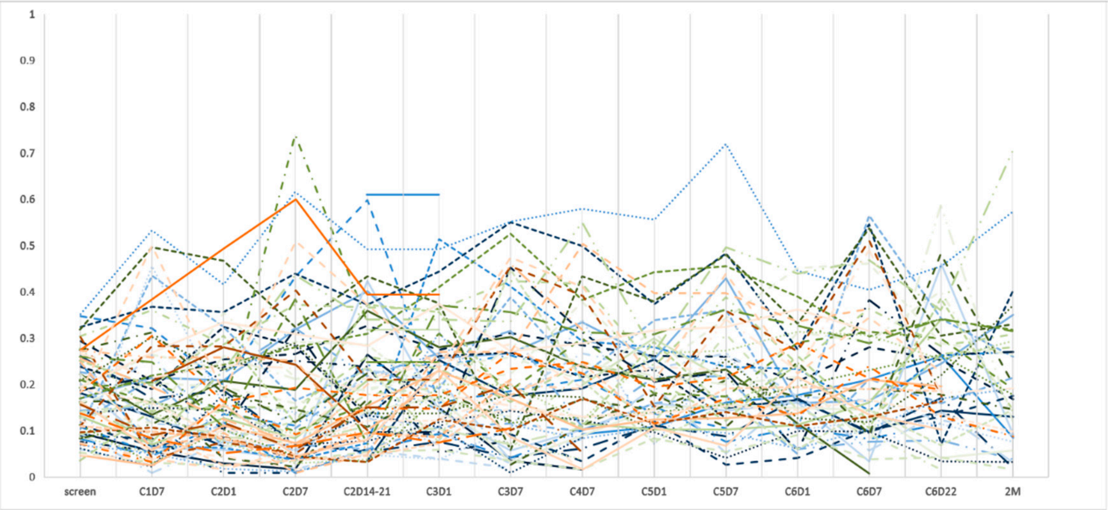

H

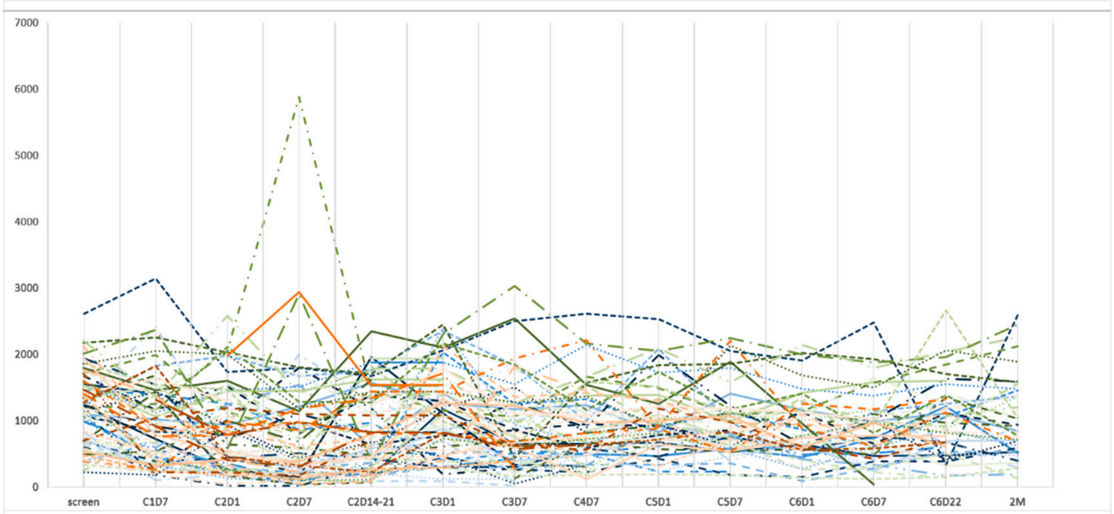

Suppementary Figure S7. The detail of patients' lymphocyte percentage (A,C,E,G) and lymphocyte count (B,D,F,H). We only showed the detail data of patients who was still in the trial at cycle2day14-21. Blue color line: patients in powder group, n=30. Green color line: patients in capsule group, n=29; Orange color line: patients in control group, n=23; (D) merge 3 groups. Yeast derived β-glucan supplementation was initiated on day 7 of cycle 2 of systemic chemotherapy.
