# Supplementary material for: Beta-Glucan Supplementation Improves Clinical Outcomes and Preserves Immune Homeostasis in Patients with Advanced Solid Tumors Receiving Chemotherapy: A Prospective Phase II Randomized Three-Arm Clinical Trial
Source: Int J Mol Sci. 2026 Aug 11;27(16):7189. doi: 10.3390/ijms27167189 (PMC13513407; doi:10.3390/ijms27167189)
Supplement: Supplementary file 1 [file ijms-27-07189-s001.zip › Supplementary Tables.pdf]

Supplementary Tables

Table S1. Sensitivity analysis using generalized estimating equations (GEE) for key flow cytometry markers

**Immune marker**	**Group effect P**	**Time effect P**	**Group × Time interaction P**	**Interpretation**
CD3(-)CD158i(+) (%)	0.728	0.919	**<0.001**	Significant longitudinal trajectory among groups
CD3(+)CD20(+) (%)	**<0.001**	0.013	**<0.001**	Significant group, time, and interaction effects
CD3(-)CD56(+) (%)	0.136	0.554	**<0.001**	Different longitudinal NK-cell trajectory
CD3(-)CD279(+) (%)	0.089	0.175	**<0.001**	Significant interaction across chemotherapy
CD3(-)CD16(+) (%)	0.175	0.408	0.140	No significant interaction
CD3(+)CD279(+) (%)	0.930	**0.003**	0.151	Significant time effect only
CD3(-)CD158b(+) (%)	0.116	0.239	0.585	No significant interaction
CD3(-)CD20(+) (%)	0.357	0.083	0.949	No significant longitudinal difference

Data were analyzed using generalized estimating equations (GEE) with an exchangeable working correlation structure. The model included treatment group, time, and group × time interaction. P values shown are from Wald tests. GEE was performed as a sensitivity analysis to confirm the robustness of the primary linear mixed-effects model.

Table S2. Estimated marginal means (EMMs) of key immune-cell subsets estimated by generalized estimating equations (GEE).

Values are presented as EMM (95% CI); observed n. Group 1, powder formulation; Group 2, capsule formulation; Group 3, control.

Immune marker	Group	Screen	C2D14-21	C6D22	2M
CD3(+)CD20(+) (%)	G1/Powder	1.12 (0.25-2.00); n=31	0.70 (0.28-1.11); n=30	0.51 (0.04-0.97); n=20	0.30 (0.19-0.40); n=19
	G2/Capsule	0.81 (0.45-1.16); n=29	1.05 (0.14-1.97); n=29	0.45 (0.22-0.67); n=21	0.39 (0.19-0.60); n=19
	G3/Control	0.14 (0.07-0.20); n=9	3.31 (3.30-3.31); n=1	-0.48 (-1.42-0.46); n=0	-0.69 (-1.51-0.12); n=0
CD3(-)CD20(+) (%)	G1/Powder	6.07 (4.07-8.07); n=31	4.04 (2.47-5.61); n=30	3.89 (2.17-5.60); n=20	3.35 (2.21-4.48); n=19
	G2/Capsule	4.55 (3.44-5.66); n=29	2.84 (1.76-3.91); n=29	2.88 (0.87-4.88); n=21	2.74 (1.77-3.71); n=19
	G3/Control	6.89 (0.59-13.18); n=9	4.96 (3.54-6.37); n=1	4.70 (-1.97-11.37); n=0	4.16 (-2.47-10.79); n=0
CD3(+)CD279(+) (%)	G1/Powder	13.01 (9.42-16.60); n=31	18.93 (14.67-23.20); n=31	15.78 (11.32-20.24); n=20	11.55 (8.78-14.32); n=19
	G2/Capsule	12.79 (9.83-15.75); n=30	14.05 (11.35-16.75); n=29	13.70 (9.52-17.87); n=21	10.94 (7.04-14.84); n=19
	G3/Control	12.02 (8.06-15.98); n=9	10.91 (9.79-12.03); n=1	14.79 (7.70-21.89); n=0	10.56 (4.52-16.60); n=0
CD3(-)CD279(+) (%)	G1/Powder	3.50 (2.14-4.86); n=31	4.76 (3.17-6.35); n=31	4.46 (2.07-6.85); n=20	4.27 (2.76-5.78); n=19
	G2/Capsule	3.05 (1.76-4.33); n=30	3.37 (2.19-4.55); n=29	4.28 (1.90-6.66); n=21	5.71 (3.35-8.07); n=19
	G3/Control	7.45 (3.74-11.16); n=9	-0.44 (-1.67-0.78); n=1	8.41 (3.91-12.91); n=0	8.22 (4.17-12.27); n=0
CD3(-)CD16(+) (%)	G1/Powder	20.41 (15.53-25.29); n=31	22.11 (16.74-27.48); n=31	18.30 (12.94-23.67); n=20	22.21 (17.05-27.38); n=19
	G2/Capsule	25.47 (20.16-30.79); n=30	24.97 (19.01-30.92); n=29	21.01 (14.89-27.12); n=21	28.63 (20.26-37.00); n=19
	G3/Control	32.42 (17.79-47.05); n=9	24.94 (16.41-33.47); n=1	30.32 (14.76-45.87); n=0	34.22 (18.93-49.52); n=0
CD3(-)CD56(+) (%)	G1/Powder	14.62 (10.15-19.10); n=31	15.00 (11.18-18.82); n=31	12.50 (8.88-16.11); n=20	15.09 (11.47-18.72); n=19
	G2/Capsule	19.51 (15.39-23.63); n=30	19.66 (14.93-24.38); n=29	16.14 (11.69-20.59); n=21	21.47 (14.78-28.16); n=19
	G3/Control	11.94 (3.98-19.90); n=9	40.32 (36.50-44.15); n=1	9.81 (1.01-18.61); n=0	12.41 (3.69-21.13); n=0
CD3(-)CD158b(+) (%)	G1/Powder	8.20 (5.85-10.54); n=31	9.86 (6.99-12.73); n=31	7.41 (5.42-9.40); n=20	8.95 (6.55-11.35); n=19
	G2/Capsule	12.01 (8.93-15.09); n=30	11.74 (8.20-15.27); n=29	10.60 (7.61-13.59); n=21	14.01 (9.62-18.39); n=19
	G3/Control	12.28 (5.58-18.99); n=9	12.22 (8.08-16.36); n=1	11.49 (4.36-18.62); n=0	13.03 (6.05-20.02); n=0
CD3(-)CD158i(+) (%)	G1/Powder	8.32 (4.71-11.93); n=31	8.28 (4.68-11.88); n=31	9.15 (5.20-13.11); n=20	8.66 (5.17-12.14); n=19
	G2/Capsule	10.02 (6.89-13.15); n=29	9.50 (5.99-13.01); n=29	9.31 (5.62-12.99); n=21	11.75 (7.19-16.30); n=19
	G3/Control	10.34 (5.54-15.13); n=9	-6.13 (-9.32--2.94); n=1	11.17 (5.63-16.71); n=0	10.67 (5.40-15.95); n=0

Table S3. Top group-by-time interactions identified by generalized estimating equations (GEE), ranked by interaction P value.

The group-by-time interaction term evaluates whether longitudinal immune-cell trajectories differed among treatment groups. FDR q values were calculated using the Benjamini-Hochberg method. Predicted changes represent C6D22 minus Screen based on GEE-derived EMMs.

Rank	Immune marker	Interaction P value	FDR q value	Predicted change: G1	Predicted change: G2	Predicted change: G3
1	CD3(+)CD16(+) (%)	2.28e-104	6.15e-103	-4.95	-3.74	-4.95
2	CD3(-)CD158i(+) (%)	2.67e-40	3.61e-39	0.84	-0.71	0.84
3	CD3(+)CD20(+) (%)	1.95e-39	1.75e-38	-0.62	-0.36	-0.62
4	CD3(-)CD56(+) (%)	1.15e-26	7.73e-26	-2.13	-3.37	-2.13
5	CD3(+)CD16(-) (%)	2.93e-10	1.58e-09	9.47	6.71	9.47
6	CD3(-)CD279(+) (%)	2.76e-09	1.24e-08	0.96	1.23	0.96
7	CD3(+)CD56(+) (%)	3.51e-07	1.36e-06	0.28	-0.10	0.28
8	CD3(-)CD158f(+) (%)	0.001	0.005	-1.02	-0.18	-1.02
9	CD3(-)CD158a(+) (%)	0.003	0.008	-0.52	-0.79	-0.52
10	CD3(+)CD158i(+) (%)	0.030	0.081	0.01	1.92	0.01
11	CD3(+)CD158b(+) (%)	0.037	0.091	0.16	1.39	0.16
12	CD3(+)CD158a(+) (%)	0.077	0.173	1.74	1.65	1.74

Footnote. GEE models were fitted using a Gaussian family and an exchangeable working correlation structure, with patient identifier specified as the clustering variable. Models included treatment group, time point, and the group-by-time interaction. Four time points were included: Screen, C2D14-21, C6D22, and 2 months. EMM, estimated marginal mean; CI, confidence interval; FDR, false discovery rate.
